# A new route to N-aromatic heterocycles from the hydrogenation of diesters in the presence of anilines†
†Electronic supplementary information (ESI) available. See DOI: 10.1039/c7sc01718a


**DOI:** 10.1039/c7sc01718a

**Published:** 2017-08-08

**Authors:** Yiping Shi, Paul C. J. Kamer, David J. Cole-Hamilton, Michelle Harvie, Emma F. Baxter, Kate J. C. Lim, Peter Pogorzelec

**Affiliations:** a EaStCHEM , School of Chemistry , University of St. Andrews , St. Andrews , KY16 9ST , Scotland , UK . Email: djc@st-andrews.ac.uk

## Abstract

The hydrogenation of dicarboxylic acids and their esters in the presence of anilines provides a new synthesis of heterocycles. [Ru(acac)_3_] and 1,1,1-tris(diphenylphosphinomethyl)ethane (triphos) gave good to excellent yields of the cyclic amines at 220 °C. When aqueous ammonia was used with dimethyl 1,6-hexadienoic acid, ε-caprolactam was obtained in good yield. A side reaction involving alkylation of the amine by methanol was suppressed by using diesters derived from longer chain and branched alcohols. Hydrogenation of optically pure diesters (dimethyl (*R*)-2-methylbutanedioate and dimethyl (*S*)-2-methylbutanedioate) with aniline afforded racemic 3-methyl-1-phenylpyrrolidine in 78% yield.

## Abstract 2

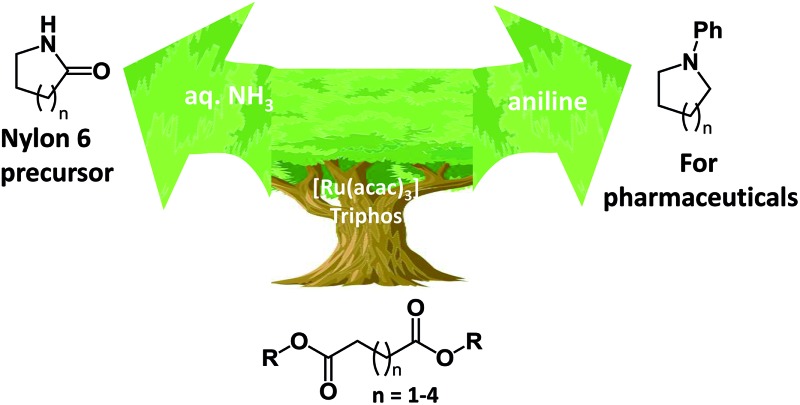
The hydrogenation of dicarboxylic acids and their esters in the presence of anilines provides a new synthesis of heterocycles.

## Introduction

Catalytic hydrogenation of amides has emerged as an environmentally attractive route to amines. The groups of Crabtree,^1^ Cole-Hamilton,^2^–^5^ Leitner^6^ and Beller^7^ have reported that amides can be successfully hydrogenated to amines using Ru/triphos catalysts and that, in contrast to reactions using heterogeneous catalysts,^8^ aromatic rings in the substrates remain untouched. Other catalysts for amide hydrogenation generally lead to C–N rather than C–O cleavage products giving alcohols from the carboxylate reside^9^–^14^ or to partial reduction.^15^,^16^

**Scheme 1:**
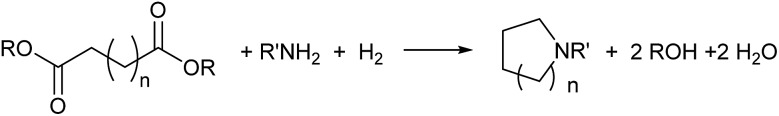
Proposed route to heterocycles from hydrogenation of dicarboxylic acid derivatives in the presence of amines.

Following this successful development, we now report the use of diesters as substrates, since they have the potential to produce saturated N-heterocycles, which are highly important building blocks for pharmaceuticals and notoriously difficult to synthesise.^17^ Common routes involve constructing an amine with a chain terminated by a group with which the amine can cyclise such as a halide or double bond (hydroamination), transformations of previously formed rings or C–H aminations using azides.^17^ Recently, partial hydrogenation of quinolines has been shown to give tetrahydroquinolines.^18^ Hydrogenation of NH lactams can give N-heterocycles,^14^,^19^ with alkylation of the N atom if the reactions are carried out in alcohols.^20^ A simple route involving the hydrogenation of dicarboxylic acid derivatives in the presence of an amine (Scheme 1) would represent a step change in the synthesis of N-heterocycles.

To our knowledge the only report of a reaction of this kind gives lactams rather than saturated N-heterocycles. Thus hydrogenation of maleic or 1,6-hexanedioic acids in the presence of methylamine and Ru/triphos gives respectively *N*-methylpyrrolidone or *N*-methyl-ε-caprolactam with >80% selectivity. The related N-heterocycles were obtained as side products in small amounts (<10%).^21^ Using dimethyl 1,6-hexanedioate and liquid ammonia in a fluorinated solvent gave 1,6-hexanediamide as the major product, but ε-caprolactam was also obtained (17%).^22^ Pyrrolidones can be produced from levulinic acid and primary amines in the presence of hydrogen^23^,^24^ or silanes,^25^,^26^ but pyrrolidines can only be formed if silanes are the reducing agents.^25^,^26^ These reactions introduce the amine *via* a Schiff base condensation with the ketone function in levulinic acid.

## Results

Our initial studies were centred around 1,6-hexanedioate esters for two reasons. Firstly, 1,6-hexanedioate esters can be easily and sustainably obtained, either from biomass, such as glucose by catalytic or enzymatic methods,^14^,^15^ or from 1,3-butadiene by palladium catalysed alkoxycarbonylation reactions.^27^–^29^ The second reason for us to use 1,6-hexanedioate esters is because our initial reactions focussed on ε-caprolactam, which can be used as a precursor for the synthesis of Nylon-6. ε-Caprolactam is industrially generated by the Beckmann rearrangement from cyclohexanone.^30^ However, cyclohexanone is produced by oxidation of cyclohexane, with high selectivity only being obtained at 10–12% conversion per pass.^31^ Concentrated sulfuric acid is involved in the reaction and ammonium sulfate is produced as the major waste product, making this route costly and environmentally unattractive.^32^

The previous report^21^ of the attempted ε-caprolactam synthesis from dimethyl 1,6-hexanedioate used liquid ammonia and only produced low yields of the desired product (<18%). Crabtree *et al.* showed that these types of hydrogenation reactions proceed better in the presence of water^1^ and we have shown^2^ that water and small amounts of acid are essential for high conversions in amide hydrogenation to amines. We, therefore, reacted dimethyl 1,6-hexanedioate with aqueous ammonia in the presence of Ru/triphos and acid (Table 1) using the conditions that had been optimised for the hydrogenation of amides to amines in our previous studies,^2^ where we also reported details of the complexes present in solution during and after the reactions. We used aqueous ammonia for these reactions because our previous studies,^4^ which we have since confirmed, gave much better conversions in simple amide hydrogenations when using aqueous ammonia rather than liquid ammonia.

**Table 1:** Cyclisation of dimethyl 1,6-hexanedioate in the presence of aqueous ammonia^*a*^

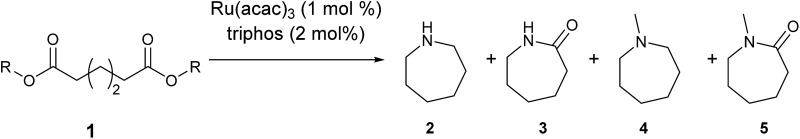							
Entry	R	*t* (h)	Conv. (%)	Sel. 2 (%)	Sel. 3 (%)	Sel. 4 (%)	Sel. 5 (%)
1	Me	20	100	1	60	16	7.2
2	Me	70	100	5.2	46	19.8	28.5
3	H	88	100	33	45	—	—

^*a*^Reagents and conditions: [Ru(acac)_3_] (1 mol%), triphos (2 mol%), MSA (1 mol%), 35% aq. NH_3_ (5 mL), dioxane (15 mL), H_2_ (10 bar), 220 °C; yield by calibrated GC-FID.

After 20 hours, ε-caprolactam **3** was obtained in 60% yield (Table 1, entry 1), a considerable improvement on the previous report.^22^ Azepane **2**, *N*-methyl azepane **4** and *N*-methyl caprolactam **5** were also observed. Methylated products **4** and **5** were formed by methylation of **2** or **3**, the methyl groups being derived from methanol. Methanol is the side product from the hydrogenation of dimethyl 1,6-hexanedioate **1**. Longer reaction time (Table 1, entry 2) led to an increased amount of methylated products, **4** and **5**. Using the diacid in place of the diester, rather similar results were obtained, but with increased amounts of **2**. No *N*-methylated products were observed (Table 1, entry 3).

We next turned our attention to using aniline instead of aqueous ammonia. Interestingly, when reacting **1** with aniline, the corresponding lactam was not formed. Instead, *N*-phenyl azepane, **6**, was predominately produced. Using one equivalent of aniline (Table 2, entry 1), **6** was obtained in 59% selectivity, and no *N*-phenyl ε-caprolactam was observed after the reaction. However, the conversion was only 82%. This low conversion could be explained by a side reaction between the aniline and the methanol formed during the hydrogenation of methyl esters as mentioned earlier reducing the availability of aniline. Mono- and di-substituted *N*-methyl anilines were both produced, the importance of this known^33^–^35^ side reaction will be discussed later. When 1.5 equivalents of aniline were used, the selectivity to compound **6** increased to 69% and the conversion was increased to 90.6% (Table 2, entry 2). However, further increasing the amount of aniline did not improve the yield of **6**, rather leading to a significant loss of selectivity (Table 2, entry 5). When 5 equivalents of aniline were used, the formation of linear difunctionalised products dominated. The linear diamine, **7**, was further methylated to give a mixture of monomethylated diamine **8** and dimethylated diamine **9** (see Scheme 3 in the Discussion section).

**Table 2:** Hydrogenation of dimethyl 1,6-hexanedioate with aniline^*a*^

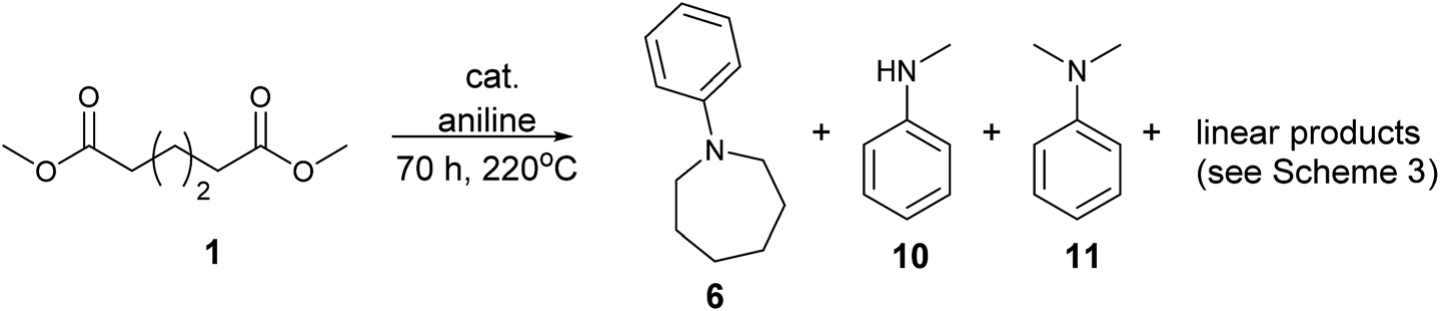			
Entry	Equiv. aniline	Conv. (%)	Sel. 6 (%)
1	1	81.5	59.0
2	1.5	90.6	69.0
3	2	94.1	51.8
4	3	91.9	54.0
5	5	86.7	12.5

^*a*^Reagents and conditions: [Ru(acac)_3_] (1 mol%), triphos (2 mol%), MSA (1 mol%), aniline (1–5 equiv.), dimethyl adipate (2.5 mmol), dioxane (15 mL), H_2_ (10 bar), 70 h, 220 °C; yields by calibrated GC-FID.

**Scheme 2:**
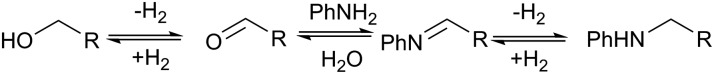
Proposed hydrogen borrowing mechanism for the alkylation between alcohol and amine.^7^

**Scheme 3:**
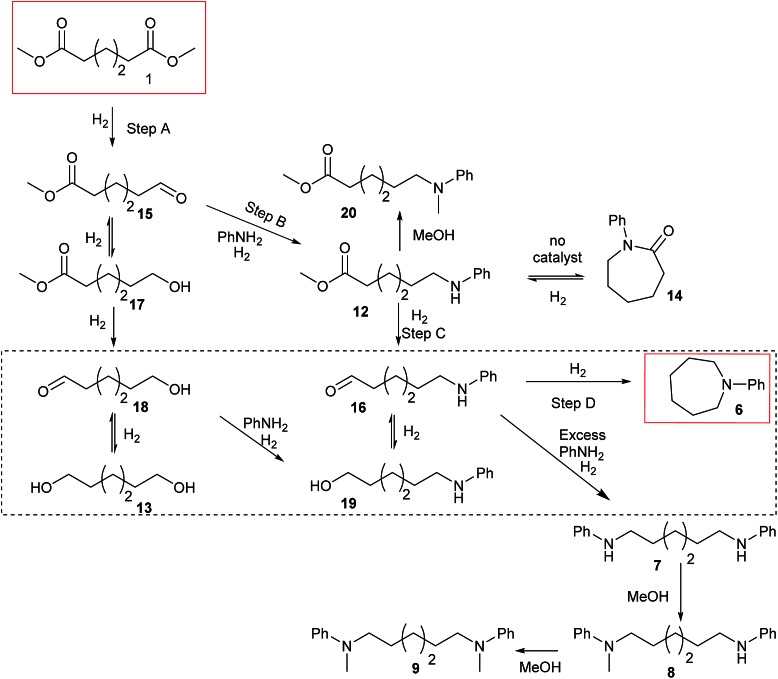
Proposed reaction pathway for diester, **1**, hydrogenation in the presence of aniline to give **6**. The main mechanism is proposed to proceed by Steps 1–4. The dotted box shows the hydrogenation of **13** to **6**. The origin of side products is also shown.

**Fig. 1:**
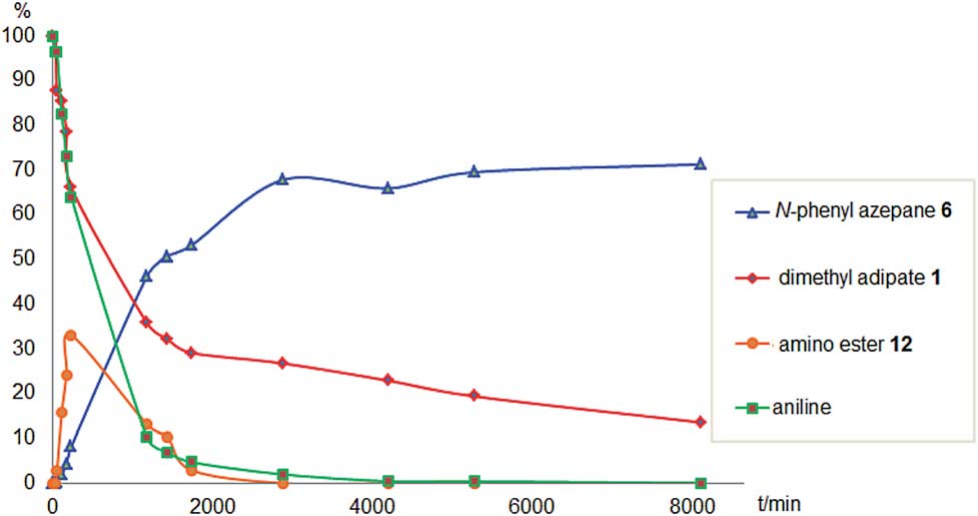
Monitoring of the hydrogenation of dimethyl 1,6-hexanedioate in the presence of aniline against time. Conditions: [Ru(acac)_3_] (1 mol%), triphos (2 mol%), MSA (1 mol%), dioxane (45 mL), dimethyl 1,6-hexanedioate (1 equiv. 7.5 mmol), aniline (1.5 equiv.), H_2_ (10 bar), 220 °C.

To gain a better understanding of the reaction, it was monitored over time (Fig. 1) using the optimised conditions (1.5 equivalent of aniline, 220 °C). Dimethyl 1,6-hexanedioate, **1**, and aniline are quickly consumed to give the monoester monoamine **12**, which slowly produces the N-heterocycle, **6**, without any further detectible intermediates. After about 66 h, no more aniline is left so **6** is no longer formed. The incomplete conversion for this cyclisation was mainly because of the side reactions occurring between produced methanol and aniline.

In an attempt to improve the selectivity, other 1,6-hexanedioate esters were studied to avoid the introduction of methyl groups. Initially, a sterically bulky ester, diisobutyl 1,6-hexanedioate, was studied (Table 3, entry 2). Pleasingly, the yield (defined as conversion × selectivity) of **6** was improved to 94% (95% conversion) with 1.5 equivalent of aniline. An increased amount of aniline in this case did not significantly influence either the conversion or the yield (Table 3, entry 3). The rate of the reaction could be increased by increasing the catalyst loading to 2 mol%. The yield of the desired cyclic amine reached 80% after 24 h, and improved to 93% after 42 h using diisobutyl 1,6-hexanedioate as the substrate (Table 3, entry 8). With these optimised conditions, the effect of different 1,6-hexanedioate esters was studied in more detail (Table 3).

**Table 3:** Study of different 1,6-hexanedioate ester substrates^*a*^

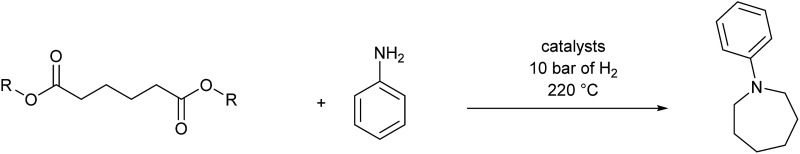			
Entry	R	Conv. (%)	Yield (%)
1^*b*^	Me	91	62
2^*b*^	2-Methylpropyl	95	94
3^*c*^	2-Methylpropyl	96	95
4	Et	98	95
5	Pr^*n*^	99	93
6	Pr^i^	92	88
7	Bu^*n*^	97	63
8	2-Methylpropyl	99	93
9	Bu^*t*^	100	80
10	2-Ethylhexyl	100	80
11	8-Methylnonyl	100	59
12	Ph	100	92
13	PhCH_2_	100	71
14	H	100	13

^*a*^[Ru(acac)_3_] (2 mol%), triphos (4 mol%), MSA (2 mol%), dioxane (15 mL), substrate (2.5 mmol), aniline (1.5 equiv.), H_2_ (10 bar), 220 °C, 42 h.

^*b*^[Ru(acac)_3_] (1 mol%), triphos (2 mol%), MSA (1 mol%), dioxane (15 mL), substrate (1 equiv., 2.5 mmol), aniline (1.5 equiv.), H_2_ (10 bar), 220 °C, 70 h.

^*c*^[Ru(acac)_3_] (1 mol%), triphos (2 mol%), MSA (1 mol%), dioxane (15 mL), substrate (1 equiv., 2.5 mmol), aniline (2 equiv.), H_2_ (10 bar), 220 °C, 70 h. Yields by calibrated GC-FID.

A wide range of diesters, both aliphatic and aromatic, was successfully cyclised to *N*-phenyl azepane **6** in good to excellent yields. Short linear or branched alkyl chains increased the yield (Table 3, entries 2–4 and 8), but longer chains (Table 3, entries 7, 10 and 11) or further branching (Table 3, entry 9) led to a slight decrease in selectivity. When di-*tert*-butyl 1,6-hexanedioate was used as the substrate (Table 3, entry 9), *N*-phenylcaprolactam was also obtained in 10% yield which was not observed in other cases. Diphenyl 1,6-hexanedioate gave excellent yields of **6** (Table 3, entry 12), but the selectivity was reduced (71%) when using the dibenzyl diester.

No amination between aniline and the corresponding alcohol was observed when using *tert*-butyl or phenyl esters, which could be explained if the hydrogen borrowing mechanism proposed by Beller's group^7^ for the amination between alcohols and amines (Scheme 2) operates rather than direct attack of the amine on the protonated alcohol. Tertiary alcohols and phenols cannot form the corresponding aldehyde; therefore, no amination reaction occurred. The alkylation of aniline proved only to be severely detrimental to the yield of **6** when using the dimethyl ester, presumably because methanol is more effective in the hydrogen borrowing reactions than the other alcohols.

Although in principle, the problem of competing amine alkylation, should be entirely eliminated by using 1,6-hexanedioic acid as the substrate, reactions under the same conditions as for the diesters gave poor selectivity to **6** (13%), with the *N*-phenyl-ε-caprolactam being the major side product (30%, Table 3, entry 14).

Five, six and eight membered N-heterocycles could also be prepared in good to excellent yields (Table 4, entries 1–3). Chiral dimethyl 2-methylbutanedioate esters were studied, the corresponding cyclic products being obtained in good yield (78%, Table 4, entries 4–6). However, the optical purity was lost after the reaction. Carrying out the reaction over only 3 h, *N*-phenyl 3-methylpyrrolidine was again the major product and the two ester amides were produced only in trace amounts, suggesting that the second hydrogenation and ring closing are much faster for the 5 than for the 7 membered ring.

**Table 4:** Cyclisation with various substrates^*a*^

Entry	Substrate	Conv. (%)	Product	Yield^*b*^ (%)
1	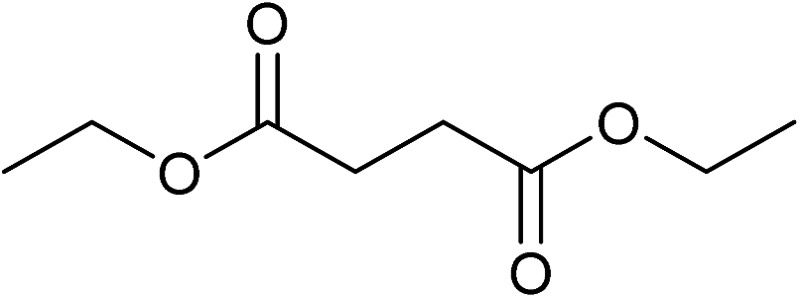	89	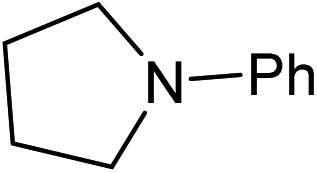	66
2	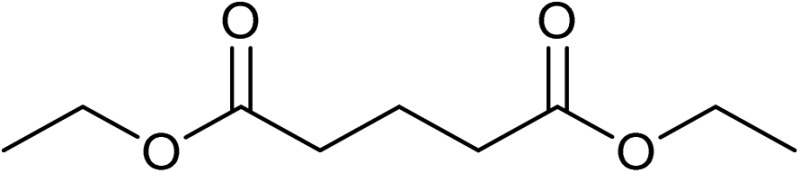	100	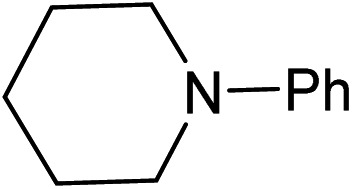	92
3	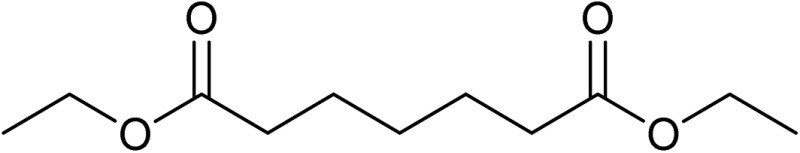	96	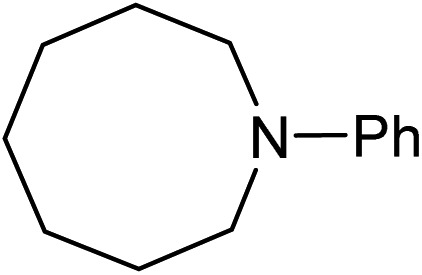	66
4	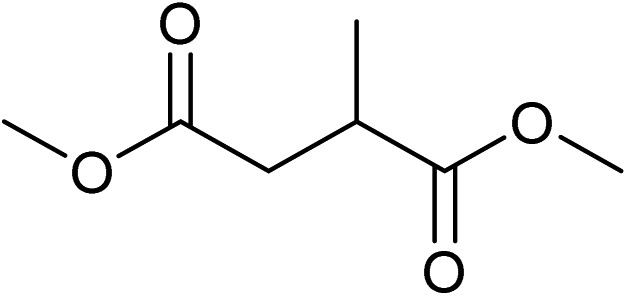	100	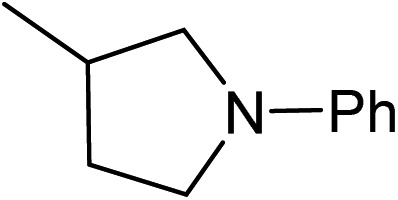	78 (75)^*c*^
5	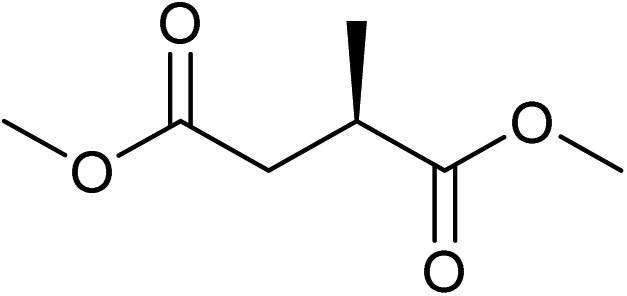	100	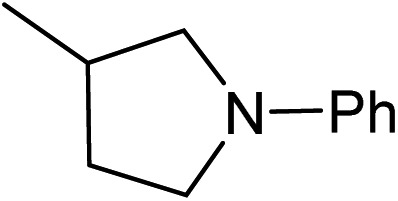	78
6	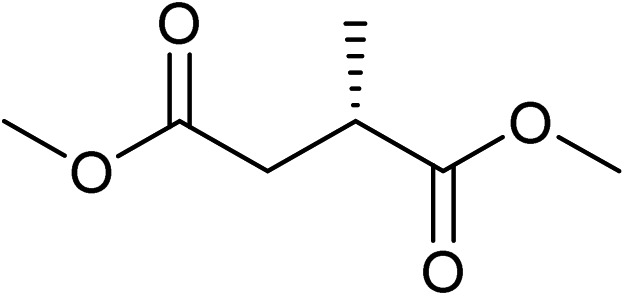	100	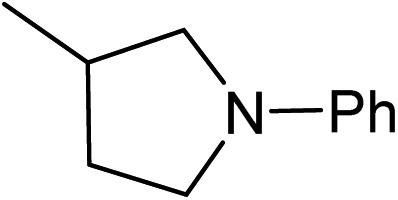	79
7	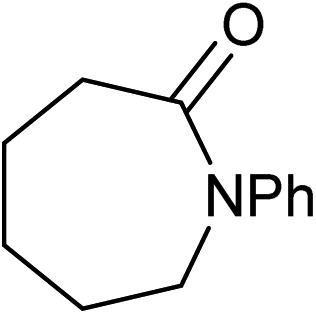	39	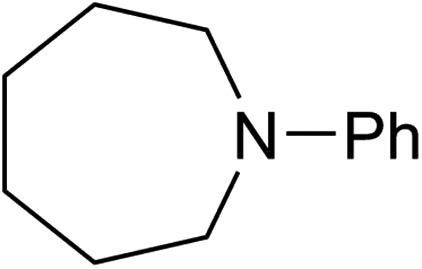	35

^*a*^Conditions as in Table 3, footnote *a*.

^*b*^NMR yield.

^*c*^Isolated yield.

Beller has shown by sampling a reaction over time that amide hydrogenations using Ru/triphos and Lewis acids, preferably Yb(OTf)_3_·H_2_O, occur *via* initial formation of the free alcohol followed by amination by a hydrogen borrowing mechanism.^7^ The amination of alcohols by this system has been reported separately.^36^

To test if a mechanism involving alcohol intermediates could also be viable under our conditions, 1,6-hexane-diol, **13**, was reacted with aniline under the same conditions as used for the carboxylic acid eaters. Hydrogen was included, although it may not be necessary, because we wished to use identical conditions to those used in the heterocycle forming reactions. When one equivalent of aniline was used, 92% yield of *N*-phenyl azepane **6** was obtained without formation of the lactam (Table 5, entry 1). With 5 equivalents of aniline, the diol, **13**, was mainly converted to the corresponding diamine, **7** (88%), with less than 10% yield of the azepane, **6** (Table 5, entry 2). This result is consistent with the lower selectivity to **6** and the formation of linear products when larger amounts of aniline are used (Table 2, entry 5) in reactions starting from dimethyl 1,6-hexanedioate and suggests that 1,6-diaminohexane does not cyclise to **6** under the reaction conditions.

**Table 5:** Hydrogenation 1,6-hexane-diol **13** with aniline^*a*^

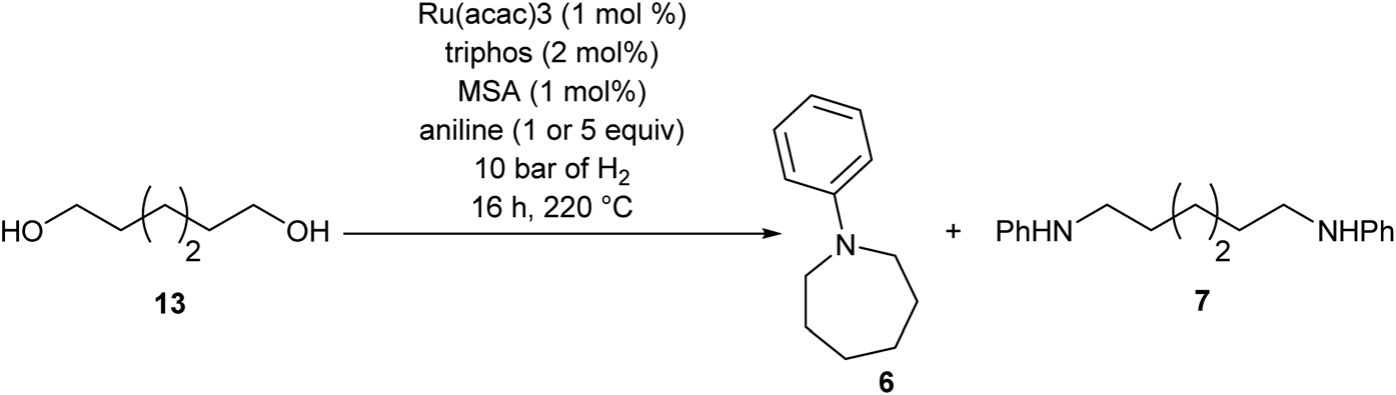				
Entry	Equiv. aniline	Conv. (%)	Yield. **6** (%)	Yield. **7**(%)
1	1	100	92	0
2^*b*^	5	100	9.5	88

^*a*^[Ru(acac)_3_] (1 mol%), triphos (2 mol%), MSA (1 mol%), dioxane (15 mL), H_2_ (10 bar), 220 °C, 16 h; yields by calibrated GC-FID.

^*b*^NMR yield.

When ester amine **12** was used as the starting material, in the absence of aniline, 62% yield of **6** was obtained after 20 hours under the normal catalytic conditions. When heating the ester amine in the absence of catalysts at 220 °C, *N*-phenyl lactam, **14**, was observed. **14** could also be hydrogenated to **6** under similar conditions, but the conversion (39%) and yield (35%) of the hydrogenation were lower, suggesting that **14** is not an intermediate since it does not build up during the hydrogenation of hexanedioate esters.

As shown in ESI Table S1† the reaction works well with 2 or 4-fluoroaniline. More strongly electron withdrawing nitro groups in the 4 or the 2,6-positions completely inhibit the reaction leading to catalyst decomposition in the case of 2,6-dinitroaniline. 2,6-Dimethyl aniline gives 54% yield at 100% conversion whilst 1,4-dibenzodioxan-6-amine performs well (100% conversion, 96% yield). The reaction is less successful with alkyl and allyl amines (ESI Table S1†), the best result being obtained with benzylamine, which gives 53% yield of *N*-benzylazepane at 92.4% conversion when starting from bis(2-methylpropyl) 1,6-hexanedioate.

## Discussion

Summarising the results described above, *N*-phenyl heterocycles such as **6** can be formed in high yield by the Ru/triphos catalysed hydrogenation of diesters in the presence of aniline. The only observable intermediate is the amino ester, **12**. In the absence of catalyst, **12** converts to *N*-phenyl ε-caprolactam, **14**, which can also be hydrogenated to **6**, but more slowly than **1**. Diol, **13**, is converted smoothly to **6** but gives linear diamine, **7**, in the presence of excess aniline. A number of the products (**8**, **9** and **20**) are formed by *N*-methylation if the substrate is a dimethyl diester.

Beller and co-workers have shown by sampling the reaction over time that amide hydrogenations using Ru/triphos and a Lewis acid in place of a Brønsted acid proceed *via* the formation of an alcohol which then undergoes amination by a hydrogen borrowing mechanism.^7^ In our experiments using methane sulfonic acid, we do not see the formation of free alcohol during the reaction. DFT calculations on the hydrogenation of methyl benzoate to benzyl alcohol suggest that free benzaldehyde is an intermediate.^6^ The hydrogen borrowing mechanism proposed for the transformation of an intermediate alcohol into an amide involves the formation of the same aldehyde as is intermediate in the amide hydrogenation reaction. We cannot, therefore, distinguish in our system whether ester alcohol, **17**, is intermediate or whether aldehyde **15** is trapped by the amine before hydrogenation. Which occurs will depend on the relative rates of hydrogenation and reaction with aniline of **15**. We do know that diol, **13**, transforms to **6** under the reaction conditions so it cannot be ruled out as an intermediate on that basis.

The simplest mechanism that is consistent with our observations is that shown in Scheme 3, Steps A–D. It involves hydrogenation of the diester, **1**, to the ester aldehyde, **15** (Scheme 3, Step A), which is trapped by aniline to give an imine (not shown), which in turn is hydrogenated in to the observed ester amine, **12** (Scheme 3, Step B). **12** is then hydrogenated to the amino aldehyde, **16**, (Scheme 3, Step C) which ring closes and is hydrogenated to **6** (Scheme 3, Step D). Also shown in Scheme 3 are the possible side reactions leading to the diol, **13**, which also gives **6** under the reaction conditions (dotted box); the formation of *N*-phenyl ε-caprolactam, **14**, from **12** in the absence of catalyst and its hydrogenation to **6**, when catalyst is present, which is rather inefficient; the formation of diamine, **7** from **13** when excess aniline is used and the methylation of various products and intermediates to give **8**, **9** and **20**.

**Scheme 4:**
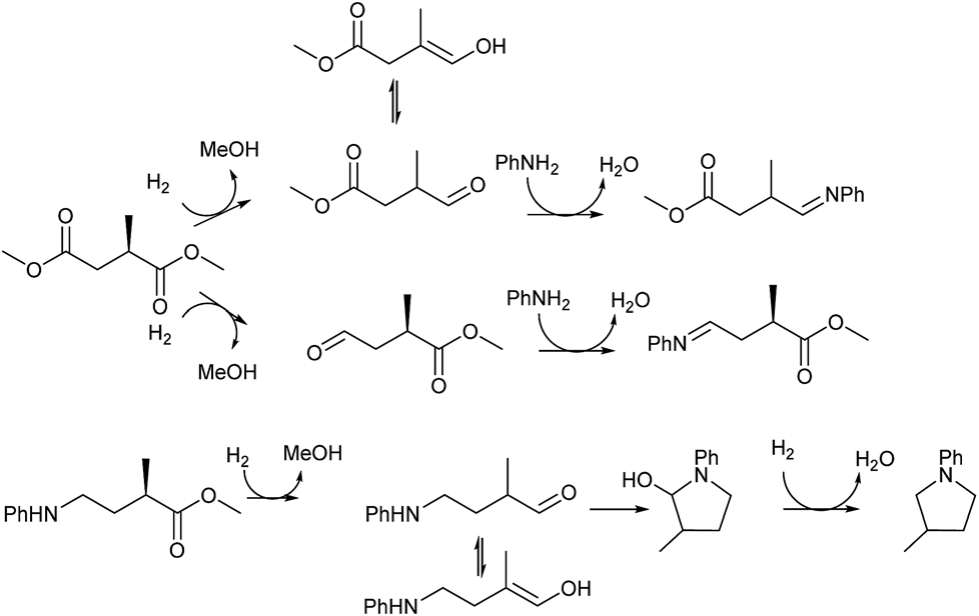
Proposed steps in which racemisation of the chiral centre originally in 2-methylsuccinic acid occurs during hydrogen borrowing steps in the formation of 2-methyltetrahydropyrrole.

The intermediacy of aldehydes in the formation of **12** from **1** and of **6** from **12** is consistent with the results of reactions using (*S*) or (*R*)-dimethyl 1,4-butanedioate (Table 4, entries 5 and 6), which both give racemic *N*-phenyl-3-methylpyrolidine. This result implies that the chiral carbon atom becomes planar during the reaction and this is most likely to be as a result of enolisation of the intermediate aldehydes, which is highly likely at 220 °C in the presence of amine and added acid. Dimethyl 1,4-butanedioate can form two amino esters (Scheme 4). In one the chiral carbon is α to the carbonyl in the aldehyde so will racemise in this step, whilst in the other it is β to the carbonyl so should be configurationally stable. The observation that the final product is racemic strongly suggests that the final ring closing step also involves an enolisable aldehyde since this will racemise the chiral centre that remained stable during the first step (Scheme 4). Unfortunately, the ester amides in this case are only formed in trace amounts even after short reaction times so it is not possible to measure their optical purity nor absolute configuration.

## Conclusions

We have developed a new simple and selective route to *N*-phenyl heterocycles from the hydrogenation of diesters in the presence of aniline and a Ru/triphos catalyst. When using methyl esters, a competing methylation of the aniline compromises the conversion, but other esters give excellent conversion and selectivity. The reaction is believed to undergo hydrogenation of the diester to the corresponding ester aldehyde, followed by reductive amination, hydrogenation of the second ester again to an aldehyde and cyclisation to the corresponding heterocycle. The presence of enolisable aldehyde intermediates in both steps explains the racemisation at the chiral C atom when starting from enantiopure dimethyl 2-methyl-1,4-butanedioate. In the presence of aqueous ammonia, ε-caprolactam was obtained in 60% yield from dimethyl 1,6-hexanedioate.

## Conflicts of interest

There are no conflicts of interest to declare.

## Supplementary Material

Supplementary information
