# Supplementary material for: A new route to N-aromatic heterocycles from the hydrogenation of diesters in the presence of anilines
Source: Chem Sci. 2017 Aug 8;8(10):6911–7. doi: 10.1039/c7sc01718a (PMC5636942; doi:10.1039/c7sc01718a)
Supplement: Supplementary file 1 [file SC-008-C7SC01718A-s001.pdf]

Electronic Supplementary Material (ESI) for Chemical Science.
This journal is © The Royal Society of Chemistry 2017

A new route to *N*-heterocycles from the hydrogenation of diesters in the presence of amines

Yiping Shi[1], David Cole-Hamilton[1]*, Paul C. J. Kamer[1] and (in part) Michelle Harvie, Emma F. Baxter, Kate J. C. Lim and Peter Pogorzelec.

[1]*EaStCHEM, School of Chemistry, University of St. Andrews, UK*

**Corresponding author:djc@st-andrews.ac.uk*

1. General Method

All the commercially available reagents were used without further purification unless specified. Diethyl adipate, diethyl succinate, diethyl glutarate, bis(2-ethylhexyl) adipate, *tert*-butyl carbamate, 1,4-dinitrobenzene, 4-nitroaniline, 2,6-dinitroaniline, 2-fluoroaniline, 1,4-benzodioxan-6-amine, isobutylaniline, *N*,*N*-dimethylaniline, adipate acid, 1-butylamine, benzylamine and 1,4-dioxane were purchased from Alfa Aesar; diisobutyl adipate, dibutyl adipate, dimethyl methyl succinate, dimethyl (*R*)-2-methylsuccinate, dodecane, ruthenium(III) acetylacetonate, 1,1,1-tris(diphenylphosphinomethyl)ethane (triphos), 2,6-dimethylaniline and *N*-methylaniline were purchased from Sigma Aldrich; diisopropyl adipate, diisodecyl adipate, dimethyl (*S*)-2-methylsuccinate, diethyl heptanedioate, 4-fluoroaniline and methyl 6-bromohexanoate were purchased from Fluorochem. Aniline was distilled over zinc powder and KOH under vacuum. Air sensitive or moisture sensitive reactions were carried out under argon in a fume hood using standard Schlenk techniques with oven-dried glassware. Flash column chromatography was performed manually using silica gel (pore size 60 Å, 70-230 mesh particle size, 40-63 μm particle size). Analytical TLC was performed on pre-coated polyester sheets of silica (60 F254 nm) and visualised by short-wave UV light at 254nm. Permanganate TLC stains was used for compounds with no UV visible chromophore. Ninhydrin stain was also used for primary and secondary amines, which gave a dark purple spot for primary amine, and a yellow/orange spot for secondary amines. Mass spectra were recorded on a Micromass LCT with a TOF mass spectrometer coupled to a Waters 2795 HPLC and a Waters 2996 detector. NMR spectra were recorded on BrukerAvance II 400 and Bruker Avance II 500 spectrometers, ^{13}C spectra were measured with ^{1}H decoupling. Residual protio peaks from deuterated solvents were used as reference with TMS at

0 ppm. GC was run with a Thermo Scientific Trace 1300 Gas Chromatography (Rtx®-35ms, 30 m × 0.25 mm (ID) × 0.5 μm (df), Crossbond® 35 % diphenyl/ 65 % dimethyl polysiloxane); Data was analysed using a Chromeleon data system. Method: 0-50 °C, ramp rate 20 °C/min, hold for 4 mins; 50-130 °C, ramp rate 20 °C/min, hold for 2 mins; 130-220 °C, ramp rate 20 °C/min, hold for 15.5 mins.

GC for chiral compounds was performed on a Thermo Trace GC Ultra (Beta DEX™ 225, 30 m × 0.25 mm (ID) × 0.25 μm (df), Fused silica capillary column). Method: 90-150 °C, ramp rate 2 °C/min, hold for 5 mins.

GCMS was carried out using a Thermo electron Corporaton DSQ II for the GC, and Trace GC ULTRA Thermo Electron Corporation mass spectrometer for the MS with a Supelco SPB-35 (Poly(35% diphenyl/65% dimethyl siloxane)) column. Method: 50-300 °C, ramp rate 15 °C/min, hold for 10 mins.

2. Experimental Procedures

2.1. Synthesis of various diesters

General procedure 1: To a solution of carboxylic acid (20 g, 1.0 equiv.) in alcohol (50 mL) was slowly added concentrated sulfuric acid (0.3 equiv.). The resulting mixture was stirred at reflux for 2 h until TLC analysis indicated complete consumption of the starting material. The excess alcohol was removed under vacuum to give the crude product, which was poured into crushed ice and then extracted with dichloromethane (DCM, 5x50 mL). The organic layers were washed with 5 % aq. $NaHCO_3$ solution (50 mL), dried over anhydrous $MgSO_4$, and concentrated under reduced pressure.

2.2. Cyclisation of difunctional esters with an amine source

General procedure 2: Ruthenium(III) acetylacetonate ([Ru(acac)$_3$], 0.010-0.020 g, 0.025-0.05 mmol, 1-2 mol%), 1,1,1-tris(diphenylphosphinomethyl)ethane (triphos, 0.031-0.062 g, 0.05-0.1 mmol, 2-4 mol%) and substrate (2.5 mmol) were weighed in air and introduced into a 250 mL Hastolloy autoclave fitted with a stirrer bar. The autoclave was sealed and purged by three vacuum/Ar cycles. Methanesulfonic acid

(1.62-3.24 μL, 0.025-0.05 mmol, 1-2 mol%) in degassed 1,4-dioxane (15 mL) was introduced into the autoclave through a septum using a syringe. Amine (eg. aqueous ammonia or aniline) (1-5 equiv.) was also introduced into the autoclave. The autoclave was sealed again, connected to the high pressure system, and purged six times with 10 bar of H_2. The autoclave was then charged with 10 bar of H_2, and heated to 220 °C (p ~ 25 bar) for the required amount of time. The autoclave was then cooled, vented and opened. The crude mixtures were analysed using GC-MS, GC-FID, NMR spectroscopy, and mass spectrometry, examples of spectra are shown below. Quantitative calculations were based on the analysis of ^{1}H NMR spectra with 1,4-dinitrobenzene as an external standard, calibrated GC using dodecane as internal standard or calculated GC response factor using dodecane as internal standard. All reactions were carried out in duplicate unless indicated otherwise in Table S1 (Section 5)

Comment [d]: 30

Comment [d]: 3

2.3. Experimental results

Dimethyl adipate 1

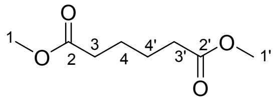

General procedure 1 was applied using adipic acid (20 g) and methanol. Dimethyl adipate was obtained as a colourless oil (20.5 g, 86 % yield). δ_H (400 MHz, $CDCl_3$) 1.61-1.69 (4H, m, $H_{4,4'}$), 2.28-2.37 (4H, m, $H_{3,3'}$), 3.66 (6H, s, $H_{1,1'}$); δ_C (101 MHz, $CDCl_3$) 24.5 ($C_{4,4'}$), 33.8 ($C_{3,3'}$), 51.7 ($C_{1,1'}$), 173.9 ($C_{2,2'}$). *The spectroscopic properties of this compound were consistent with literature data.*[1]

Di-*n*-propyl adipate

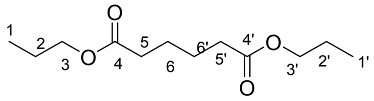

General procedure 1 was applied using adipic acid (20 g) and 1-propanol. Di-*n*-propyl adipate was obtained as a colourless oil (25 g, 80 % yield). δ_H (400 MHz, $CDCl_3$) 0.94 (6H, t, *J* = 7.2 Hz, $H_{1,1'}$), 1.54-1.73 (8H, m, $H_{2,2',6,6'}$), 2.28-2.38 (4H, m, $H_{5,5'}$), 4.03 (4H, t, *J* = 6.8 Hz, $H_{3,3'}$); δ_C (126 MHz, $CDCl_3$) 10.5 ($C_{1,1'}$), 22.1 ($C_{2,2'}$), 24.6 ($C_{6,6'}$), 34.1 ($C_{5,5'}$), 66.1 ($C_{3,3'}$), 173.6 ($C_{4,4'}$). *The spectroscopic properties of this compound were consistent with literature data.*[1]

Di-*tert*-butyl adipate

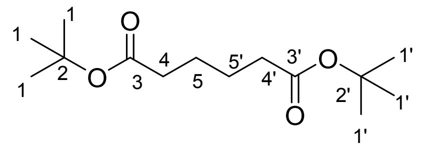

A mixture of adipic acid (4.76 g, 33 mmol, 1 equiv.), thionyl chloride (10 mL, 138 mmol, 4 equiv.) in 2:1 (v/v) benzene-cyclohexane (15 mL) was heated under reflux for 2.5 h. The reaction mixture was concentrated under vacuum to removed thionyl chloride, benzene and cyclohexane. The resulting yellow oil was dissolved in anhydrous ether (5 mL) and added dropwise to a solution of dimethylaniline (13 mL, 102 mmol, 3 equiv.), *tert*-butanol (10 mL, 105 mmol, 3 equiv.) in anhydrous ether (5 mL). The reaction mixture was stirred at room temperature for 20 hours. The reaction mixture was diluted with 10 % (w/v) aqueous sodium chloride (100 mL) and extracted 3 times with Et_2O (50 mL). The organic layer was washed with 3:1 (v/v) 2 M aqueous HCl/ sat. brine (100 mL), then with 3:1 (v/v) 1 M aqueous NaOH/ brine (2 x100 mL), then brine (100 mL). The organic layer was dried over $MgSO_4$ and concentrated under reduced pressure to afford the crude product. The orange crude product was purified by flash column chromatography (10 % EtOAc/ Petroleum ether) to afford the product as a colourless solid (6 g, 71 % yield). δ_H (400 MHz, $CDCl_3$) 1.42 (18H, s, $H_{1,1'}$), 1.48-1.66 (4H, m, $H_{5,5'}$), 2.14-2.28 (4H, m, $H_{4,4'}$); δ_C (101 MHz, $CDCl_3$) 24.7 ($C_{5,5'}$), 28.2 ($C_{1,1'}$), 35.4 ($C_{4,4'}$), 80.2 ($C_{2,2'}$), 173.0 ($C_{3,3'}$). *The spectroscopic properties of this compound were consistent with literature data.*[2] mp: 28-29 °C.

Diphenyl adipate

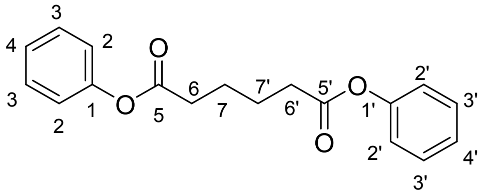

Reaction condition adopted from literature.[3] Adipic acid (15 g, 103 mmol, 1 equiv.), diphenyl carbonate (44 g, 205.4 mmol, 2 equiv.), 1,8-Diazabicyclo[5.4.0]undec-7-ene (DBU) (1.56 g, 10.3 mmol, 10 mol%) were added into a flask and heated at 160 °C for 24 h. The by-product, phenol, was removed under vacuum. The crude product was recrystalised from ethyl acetate/ hexane (1:3) to afford diphenyl adipate as a white solid (25 g, 61 %). δ_H (400 MHz, $CDCl_3$) 1.82-1.97 (4H, m, $H_{7,7'}$), 2.57-2.71 (4H, m, $H_{6,6'}$), 7.04-7.13 (4H, m, $H_{2,2'}$), 7.20-7.25 (2H, m, $H_{4,4'}$), 7.33-7.43 (4H, m, $H_{3,3'}$); δ_C (101 MHz, $CDCl_3$) 24.5 ($C_{7,7'}$), 34.1 ($C_{6,6'}$), 121.7 ($C_{2,2'}$), 126.0 ($C_{4,4'}$), 129.6 ($C_{3,3'}$), 150.8 ($C_{1,1'}$), 171.9 ($C_{5,5'}$). *The spectroscopic properties of this compound were consistent with literature data.* [3] mp: 96-98 °C.

Dibenzyl adipate

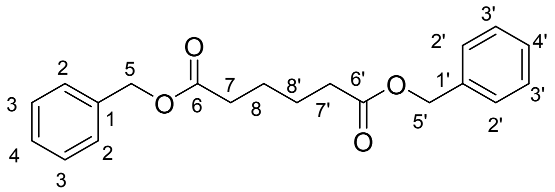

Reaction condition adopted from literature[4]. Adipic acid (20 g, 137 mmol, 1 equiv.), benzyl alcohol (32.5 g, 300 mmol, 2.2 equiv.), *p*-toluenesulfuric acid monohydrate (0.3 g, 1.6 mmol, 1.2 mol%) in toluene (20 mL) were added into a 250 mL round bottom flask. The round bottom flask was fitted to a Dean Stark condenser and heated under reflux for 16 h. The reaction was cooled and neutralized with sodium carbonate (0.4 g), then washed with water (100 g). The toluene was removed under vacuum. The crude product was washed with petroleum ether, and dibenzyl adipate was obtained as a white solid (32 g, 72 % yield). δ_H (400 MHz, $CDCl_3$) 1.63-1.76 (4H, m, $H_{8,8'}$), 2.31-2.44 (4H, m, $H_{7,7'}$), 5.11 (4H, s, $H_{5,5'}$), 7.29-7.40 (10H, m, $H_{2-4;2'-4'}$); δ_C (101 MHz, $CDCl_3$) 24.5 ($C_{8,8'}$), 34.0 ($C_{7,7'}$), 66.3 ($C_{5,5'}$), 128.3, 128.7 ($C_{2-4;2'-4'}$), 136.1 ($C_{1,1'}$), 173.2 ($C_{6,6'}$). mp: 35-37 °C.

N-phenylazepane 6

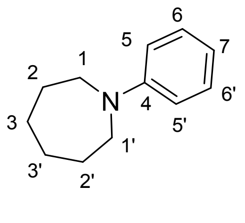

General prodecure 2 was applied using dimethyl adipate. A sample for anaylsis was purified by preparative TLC (pre-coated polyester sheets of silica (60 F254 nm)) (10 % ethyl acetate/ petroleum ether). δ_H (400 MHz, $CDCl_3$) 1.50-1.59 (4H, m, $H_{3,3'}$), 1.70-1.87 (4H, m, $H_{2,2'}$), 3.45 (4H, t, *J* = 6.0 Hz, $H_{1,1,}$), 6.62 (1H, t, *J* = 7.2 Hz, H_7), 6.69 (2H, d, *J* = 8.0 Hz, $H_{5,5'}$), 7.14-7.24 (2H, m, $H_{6,6'}$); δ_C (101 MHz, $CDCl_3$) 27.3 ($C_{3,3'}$), 27.9 ($C_{2,2'}$), 49.2 ($C_{1,1'}$), 111.3 ($C_{5,5'}$), 115.3 (C_7), 129.4 ($C_{6,6'}$), 149.0 (C_4). Micro Anal. Found: C, 82.09; H, 9.70; N, 8.15. Calc'd for $C_{12}H_{17}N$: C, 82.23; H, 9.78; N, 7.99. HRMS: (NSI^+) Found: $[M+H]^+$ 176.1430, $C_{12}H_{18}N$ requires 176.1434. *The spectroscopic properties of this compound were consistent with literature data.*[5]

N-phenyl caprolactam 14

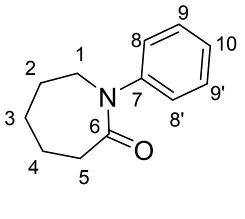

Reaction condition adopted from literature.[6] ε-Caprolactam (4.53 g, 40 mmol), $[Pd_2(dba)_3]$ (405 mg, 0.4 mmol, 1 mol%), 4,5-bis(diphenylphosphino)-9,9-dimethylxanthene (767 mg, 1.3 mmol, 3 mol%), cesium carbonate (17 g, 51 mmol) were dissolved in dioxane (40 mL) in a flask, bromobenzene (3.8 mL, 36 mmol) was added slowly into the flask. The reaction mixture was heated under reflux for 16 h. The reaction mixture was then cooled and filtered to removed palladium catalyst, and concentrated under reduced pressure. The crude product was purified by flash column chromatography (40 % ethyl acetate/ petroleum ether) to afford the product as a yellow solid (6.5 g, 86 % yield). δ_H (400 MHz, $CDCl_3$) 1.83 (6H, app s, $H_{2,3,4}$), 2.67-2.76 (2H,

m, H_5), 3.69-3.80 (2H, m, H_1), 7.15-7.25 (3H, m, $H_{8,10}$), 7.32-7.42 (2H, m, H_9); δ_H (500 MHz, d_8-toluene, 295 K) 1.24-1.34 (4H, m, $H_{2,4}$), 1.41-1.47 (2H, m, H_3) 2.34-2.40 (2H, m, H_5), 3.16 (2H, t, J = 5 Hz, H_1), 6.94-6.98 (3H, m, H_{10}), 7.12-7.17 (4H, m, $H_{8,8',9,9'}$); δ_C (101 MHz, $CDCl_3$) 23.7 (C_4), 29.1 (C_2), 30.0 (C_3), 37.8 (C_5), 53.2 (C_1), 126.4 ($C_{8,8'}$), 126.6 (C_{10}), 129.2 ($C_{9,9'}$), 144.7 (C_7), 175.7 (C_6). *The spectroscopic properties of this compound were consistent with literature data.*[6]

3-Methyl-1-phenylpyrrolidine

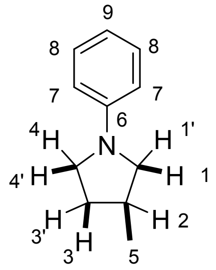

General procedure 2 was applied using dimethyl methyl succinate. Purification was by preparative TLC ((pre-coated polyester sheets of silica (60 F254 nm))) (10 % ethyl acetate/ petroleum ether). δ_H (400 MHz, $CDCl_3$) 1.15 (3H, d, J = 6.8 Hz, H_5), 1.57-1.68 (1H, m, H_3), 2.09-2.19 (1H, m, $H_{3'}$), 2.33-2.48 (1H, m, H_2), 2.84-2.92 (1H, m, $H_{1'}$), 3.24-3.42 (2H, m, $H_{4,4'}$), 3.43-3.51 (1H, m, H_1), 6.56 (2H, d, J = 7.2 Hz, H_7), 6.67 (1H, t, J = 7.2 Hz, H_9), 7.22-7.28 (2H, m, H_8); δ_C (101 MHz, $CDCl_3$) 18.6 (C_5), 33.4, 33.7 ($C_{2,3}$), 47.6 (C_4), 55.0 (C_1), 111.5 (C_7), 115.3 (C_9), 129.2 (C_8), 148.0 (C_6). *The spectroscopic properties of this compound were consistent with literature data.*[7]

***N*-phenylpyrrolidine**

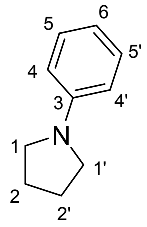

General prodecure 2 was applied using diethyl succinate. A sample for anaylsis was purified by preparative TLC (pre-coated polyester sheets of silica (60 F254 nm)) (1 % ethyl acetate/ petroleum ether). δ_H (400 MHz, $CDCl_3$) 1.97-2.11 (4H, m, $H_{2,2'}$), 3.25-3.39 (4H, m, $H_{1,1'}$), 6.57-6.66 (2H, m, $H_{4,4'}$), 6.66-6.75 (1H, m, H_6), 7.22-7.32 (2H, m, $H_{5,5'}$); δ_C (101 MHz, $CDCl_3$) 25.6 (C_2), 47.7 ($C_{1,1'}$), 111.7 ($C_{4,4'}$), 115.5 (C_6), 129.3 ($C_{5,5'}$), 149.1 (C_3). HRMS: (NSI^+) Found: $[M+H]^+$ 148.1117, $C_{10}H_{14}N$ requires 148.1121.*The spectroscopic properties of this compound were consistent with literature data.*[8]

***N*-phenylpiperidine**

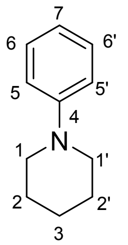

General prodecure 2 was applied using diethyl glutarate. A sample for anaylsis was purified by preparative TLC (pre-coated polyester sheets of silica (60 F254 nm)) (1 % ethyl acetate/ petroleum ether). δ_H (400 MHz, $CDCl_3$) 1.55-1.68 (2H, m, H_3), 1.70-1.84 (4H, m, $H_{2,2'}$), 3.21 (4H, t, J = 5.5 Hz, $H_{1,1'}$), 6.88 (1H, tt, J = 7.2, 1.0 Hz, H_7), 6.95-7.04 (2H, m, $H_{5,5'}$), 7.26-7.35 (2H, m, $H_{6,6'}$); δ_C (101 MHz, $CDCl_3$) 24.5 (C_3), 26.0 ($C_{2,2'}$), 50.8 ($C_{1,1'}$), 116.7 ($C_{5,5'}$), 119.3 (C_7), 129.1 ($C_{6,6'}$), 152.4 (C_4). *The spectroscopic properties of this compound were consistent with literature data.*[9]

***N*-phenylazocane**

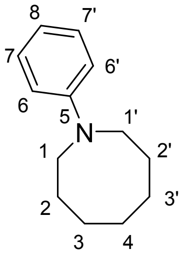

General procedure 2 applied using diethyl heptanedioate. A sample for analysis was purified by preparative TLC (pre-coated polyester sheets of silica (60 F254 nm)) (10 % ethyl acetate/ petroleum ether). δ_H (400 MHz, $CDCl_3$) 1.51-1.62 (6H, m, $H_{3,3',4}$), 1.71-1.81 (4H, m, $H_{2,2'}$), 3.45 (4H, t, J = 5.6 Hz, $H_{1,1'}$), 6.64 (1H, t, J = 7.2 Hz, H_8), 6.68 (2H, d, J = 8.0 Hz, $H_{6,6'}$), 7.17-7.26 (2H, m, $H_{7,7'}$); δ_C (101 MHz, $CDCl_3$) 27.0, 27.2, 27.4 ($C_{2,2'-4,4'}$), 50.7 ($C_{1,1'}$), 111.2 ($C_{6,6'}$), 115.1 (C_8), 129.3 ($C_{7,7'}$), 148.4 (C_5). HRMS: (ESI^+) Found: $[M]^+$ 189.1518, $C_{13}H_{19}N$ requires 189.1517. *The spectroscopic properties of this compound were consistent with literature data.*[10]

***N*-(2,3-dihydrobenzo[1,4]dioxin-5-yl)azepane**

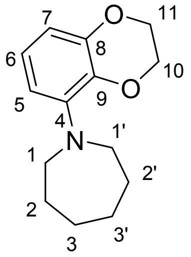

General prodecure 2 was applied using diisobutyl adipate and1,4-dibenzodioxan-6-amine. A sample for analysis was purified by preparative TLC (pre-coated polyester sheets of silica (60 F254 nm)) (10 % ethyl acetate/ petroleum ether). δ_H (500 MHz, $CDCl_3$) 1.49-1.59 (4H, m, $H_{3,3'}$), 1.70-1.83 (4H, m, $H_{2,2'}$), 3.45 (4H, t, J = 6.0 Hz, $H_{1,1'}$), 4.17-4.21 (2H, m), 4.22-4.27 (2H, m) ($H_{10,11}$), 6.19-6.25 (2H, m), 6.72-6.76 (1H, m) ($H_{5,6,7}$); δ_C (126 MHz, $CDCl_3$) 27.3 (C_3), 28.0 (C_2), 49.6 (C_1), 64.5, 65.0 ($C_{10,11}$), 100.0, 104.8, 117.6 ($C_{5,6,7}$), 134.0, 144.1, 144.6 ($C_{4,8,9}$). HRMS: (ESI^+) Found: $[M]^+$ 233.1412, $C_{14}H_{19}NO_2$ requires 233.1416.

***N*-(4-fluorophenyl)azepane**

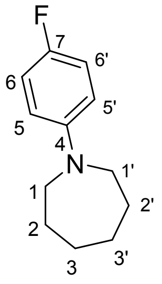

General prodecure 2 was applied using diisobutyl adipate and 4-fluoroaniline. A sample for anaylsis was purified by preparative TLC (pre-coated polyester sheets of silica (60 F254 nm)) (10 % ethyl acetate/ petroleum ether). δ_H (500 MHz, $CDCl_3$) 1.56 (4H, m, $H_{3,3'}$), 1.79 (4H, m, $H_{2,2'}$), 3.44 (4H, t, J = 6.0 Hz, $H_{1,1'}$), 6.61 (2H, m, $H_{5,5'}$), 6.93 (2H, t, J = 9.0 Hz, $H_{6,6'}$); δ_C (126 MHz, $CDCl_3$) 27.2 ($C_{3,3'}$), 27.9 ($C_{2,2'}$), 49.6 ($C_{1,1'}$), 111.8 (d, J = 7.1 Hz, $C_{5,5'}$), 115.6 (d, J = 21.8 Hz, $C_{6,6'}$), 145.7 (C_4), 154.6 (d, J = 233.4 Hz, C_7). δ_F (376 MHz, $CDCl_3$) -131.3. HRMS: (ESI^+) Found: $[M]^+$ 193.1262, $C_{12}H_{16}NF$ requires 193.1267.

***N*-(2-fluorophenyl)azepane**

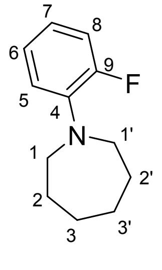

General prodecure 2 was applied using diisobutyl adipate and 2-fluoroaniline. A sample for anaylsis was purified by preparative TLC (pre-coated polyester sheets of silica (60 F254 nm)) (1 % ethyl acetate/ petroleum ether). δ_H (400 MHz, $CDCl_3$) 1.58-1.68 (4H, m, $H_{3,3'}$), 1.78-1.87 (4H, m, $H_{2,2'}$), 3.36 (4H, td, J = 1.5 Hz, 5.8 Hz, $H_{1,1'}$), 6.70 (1H, ttd, J = 1.6, 4.3, 7.5 Hz, H_7), 6.85 (1H, ddd, J = 1.6, 8.4, 9.6 Hz, H_5), 6.92-7.03 (2H, m, $H_{6,8}$); δ_C (101 MHz, $CDCl_3$) 27.5 ($C_{3,3'}$), 29.3 ($C_{2,2'}$), 52.2 (d, J = 4.3 Hz, $C_{1,1'}$), 116.5 (d, J = 22.0 Hz, C_8), 117.2 (d, J = 4.3 Hz, C_5), 118.4 (d, J = 7.6 Hz, C_7), 124.4 (d, J = 3.3 Hz, C_6), 140.3 (d, J = 8.0 Hz, C_4), 153.7 (d, J = 242.4 Hz, C_9). δ_F (377 MHz, $CDCl_3$) -123.4. HRMS: (NSI^+) Found: $[M+H]^+$ 194.1338, $C_{12}H_{17}NF$ requires 194.1340.

***N*-(2,6-dimethylphenyl)azepane**

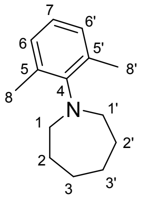

General prodecure 2 was applied using diisobutyl adipate and 2,6-dimethylaniline. A sample for anaylsis was purified by preparative TLC (pre-coated polyester sheets of silica (60 F254 nm)) (1 % ethyl acetate/ petroleum ether). δ_H (400 MHz, $CDCl_3$) 1.75 (8H, app s, $H_{2,2',3,3'}$), 2.35 (6H, s, $H_{8,8'}$), 3.12 (4H, t, J = 6.0 Hz, $H_{1,1'}$), 6.98 (1H, app dd (B part of A_2B spin system), J = 6.3 Hz, 8.4 Hz, H_7), 7.05 (2H, d, J = 7.2 Hz, $H_{6,6'}$); δ_C (101 MHz, $CDCl_3$) 19.3 ($C_{8,8'}$), 28.3, 31.6 ($C_{2,2',3,3'}$), 53.3 ($C_{1,1'}$), 124.9 (C_7), 128.8 ($C_{6,6'}$), 137.5 ($C_{5,5'}$), 151.5 (C_4). HRMS: (NSI^+) Found: $[M+H]^+$ 204.1747, $C_{14}H_{22}N$ requires 204.1746.

Comment [d]: 27

Methyl 6-(phenylamino)hexanoate 12

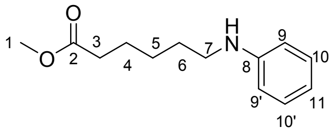

Reaction condition modified from literature.[11] Aniline (5.5 mL, 60 mmol, 1 equiv.), methyl 6-bromohexanoate (12.54 g, 60 mmol, 1 equiv.) and sodium acetate trihydrated (24.48 g, 180 mmol) in ethanol (30 mL) were heated under reflux for 16 h. The reaction mixture was then cooled and ethanol removed under reduced pressure. The crude material was redissolved in DCM, washed with water and purified by flash column chromatography (20 % ethyl acetate/ petroleum ether) to a mixture of monomer and dimer. The desired monomer was further purified by vacuum distillation (0.13 mmbar, 100 °C). The product was obtained as white crystals (5 g, 38 %). δ_H (400 MHz, $CDCl_3$) 1.41-1.48 (2H, m, H_5), 1.61-1.72 (4H, m, $H_{4,6}$), 2.34 (2H, t, J = 7.6 Hz, H_3), 3.12 (2H, t, J= 7.2 Hz, H_7), 3.61 (1H, br s, NH), 3.68 (3H, s, H_1), 6.60 (2H, d, J = 8.4 Hz, $H_{9,9'}$), 6.69 (1H, t, J = 7.6 Hz, H_{11}), 7.17 (2H, t, J = 8.0 Hz, $H_{10,10'}$); δ_C (101 MHz, $CDCl_3$) 24.8 (C_4), 26.8 (C_5), 29.3 (C_6), 34.1(C_3), 43.8 (C_7), 51.7 (C_1), 112.8 ($C_{9,9'}$), 117.3 (C_{11}), 129.4 ($C_{10,10'}$), 148.5 (C_8), 174.2 (C_2). HRMS: (NSI^+) Found: $[M+H]^+$ 222.1487, $C_{13}H_{20}NO_2$

requires 222.1489. Micro Anal. Found: C, 70.66; H, 8.62; N, 6.41. Calc'd for $C_{12}H_{17}N$: C, 70.56; H, 8.65; N, 6.33. mp: 39-41 °C.

3. NMR spectra of pure samples

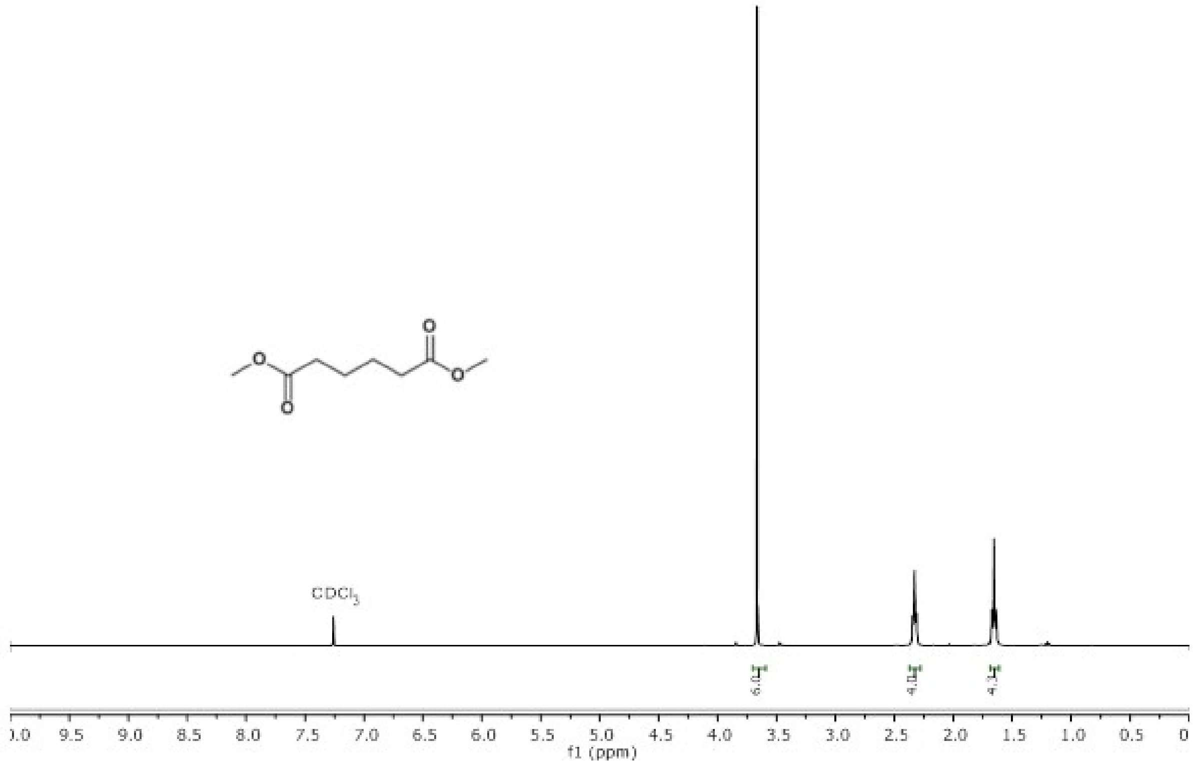

Figure 1. ^1H NMR (400 MHz, $CDCl_3$) of dimethyl adipate **1**.

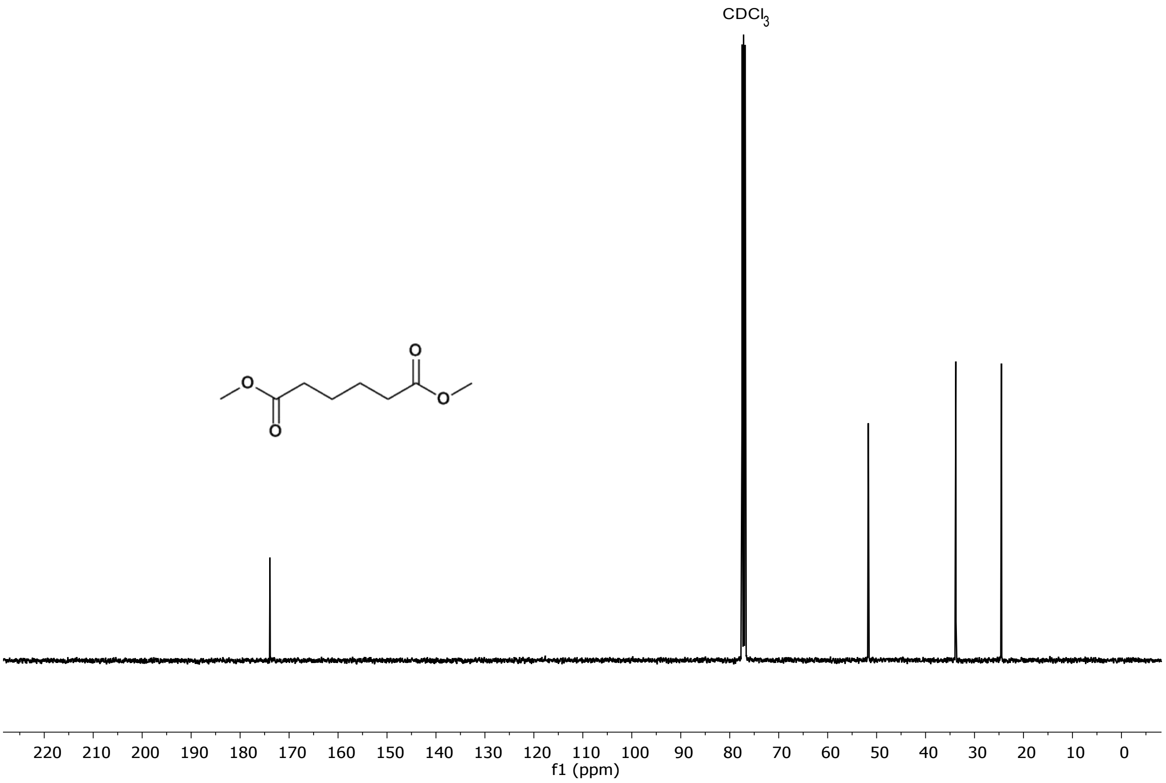

Figure 2. ^{13}C NMR (101 MHz, $CDCl_3$) of dimethyl adipate **1**.

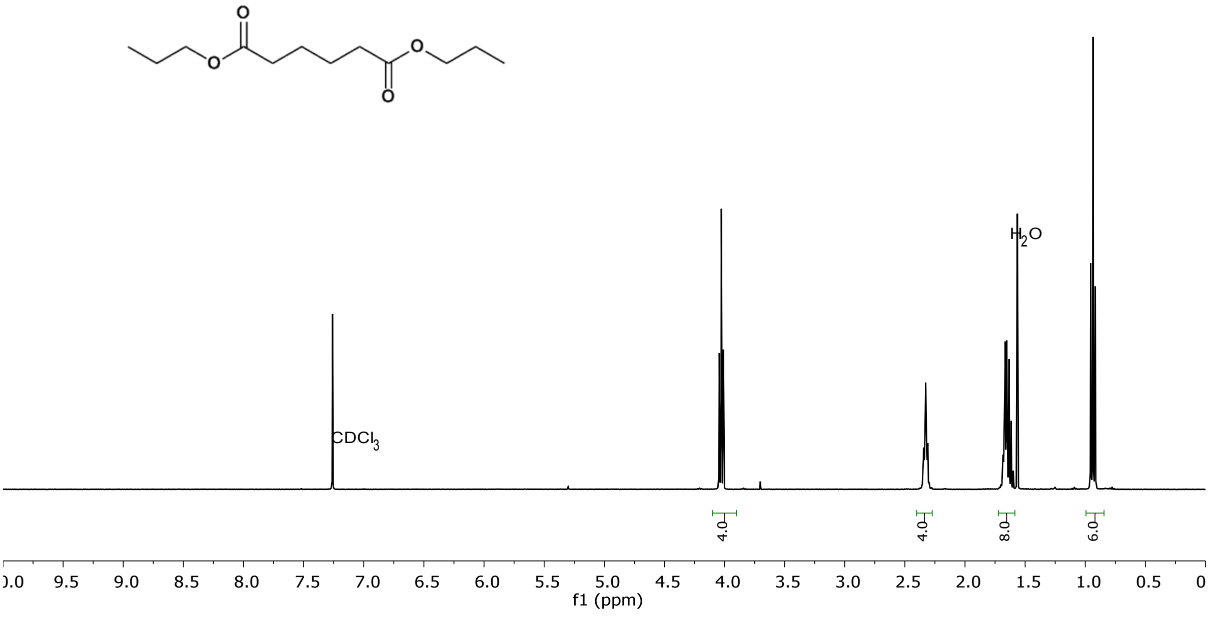

Figure 3. ^{1}H NMR (400 MHz, $CDCl_3$) of di-*n*-propyl adipate.

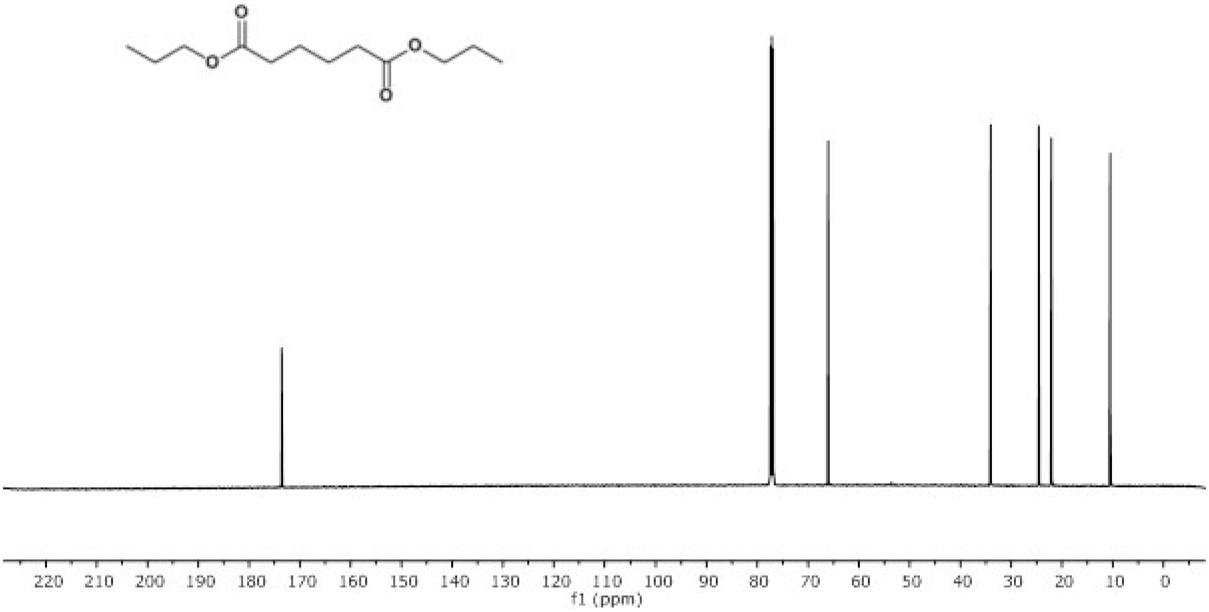

Figure 4. ^{13}C NMR (126 MHz, $CDCl_3$) of di-*n*-propyl adipate.

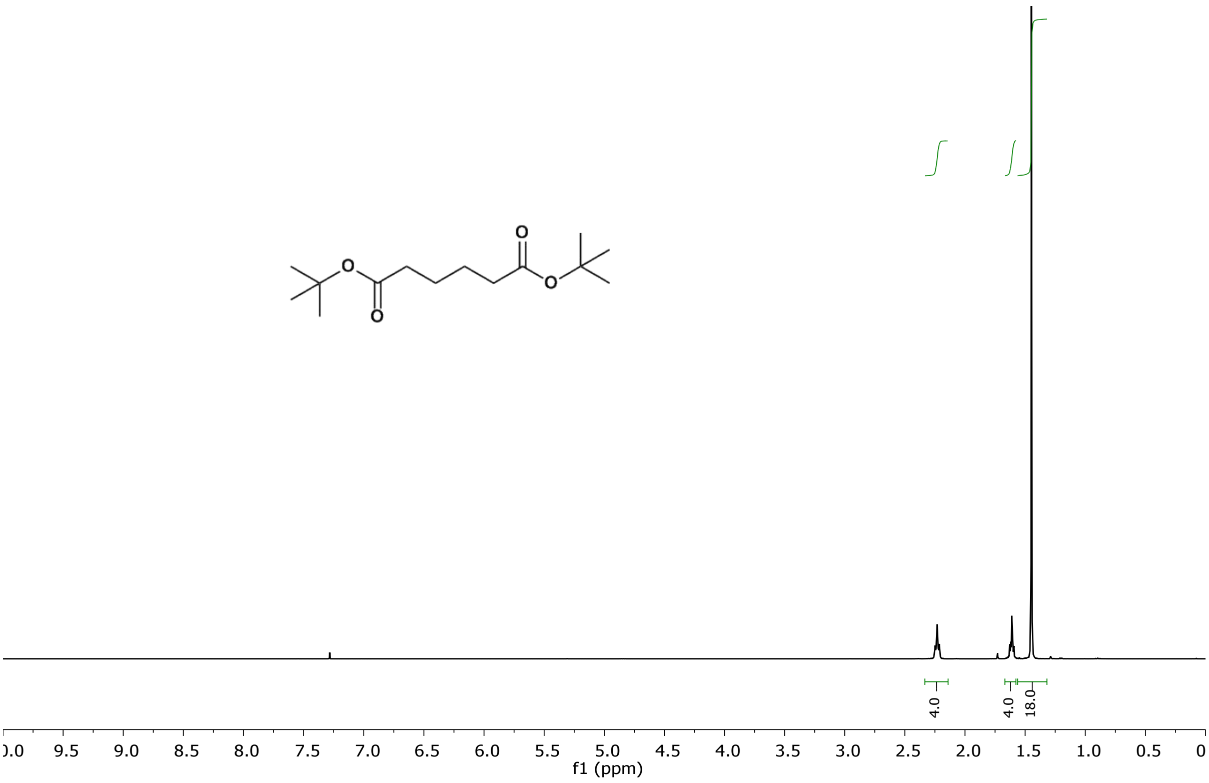

Figure 5. ^{1}H NMR (400 MHz, $CDCl_3$) of di-*tert*-butyl adipate.

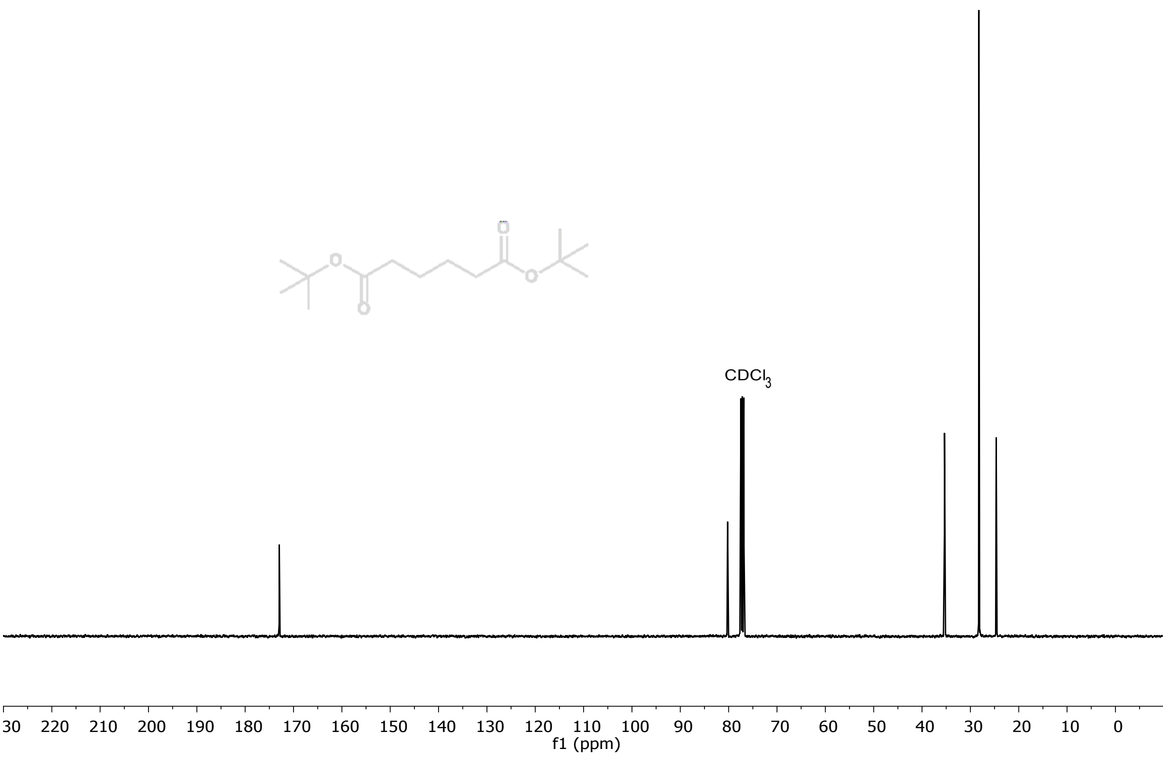

Figure 6. ^{13}C NMR (101 MHz, $CDCl_3$) of di-*tert*-butyl adipate.

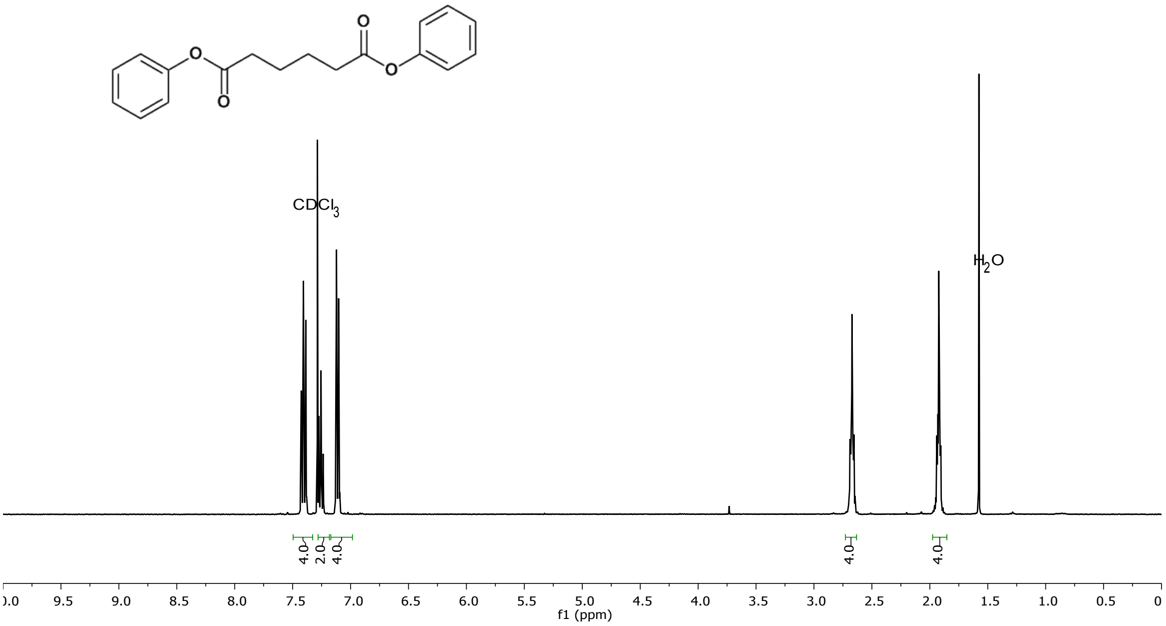

Figure 7. ^{1}H NMR (400 MHz, $CDCl_3$) of diphenyl adipate.

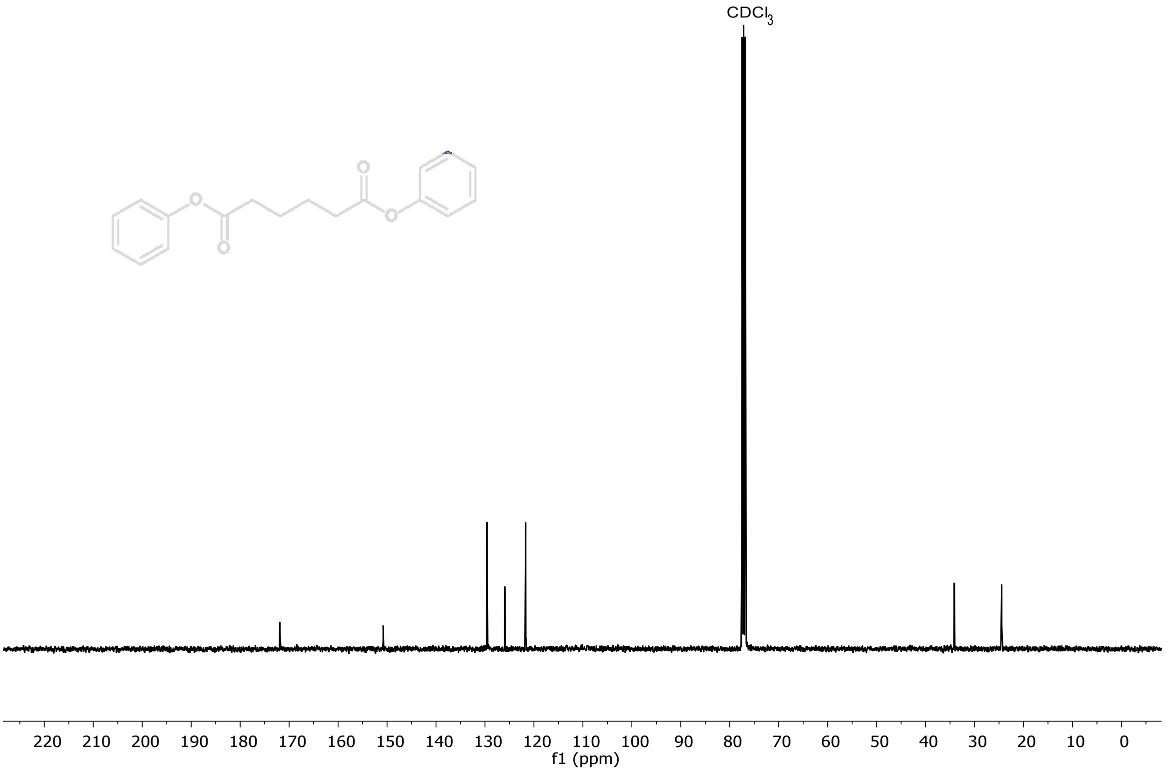

Figure 8. ^{13}C NMR (101 MHz, $CDCl_3$) of diphenyl adipate.

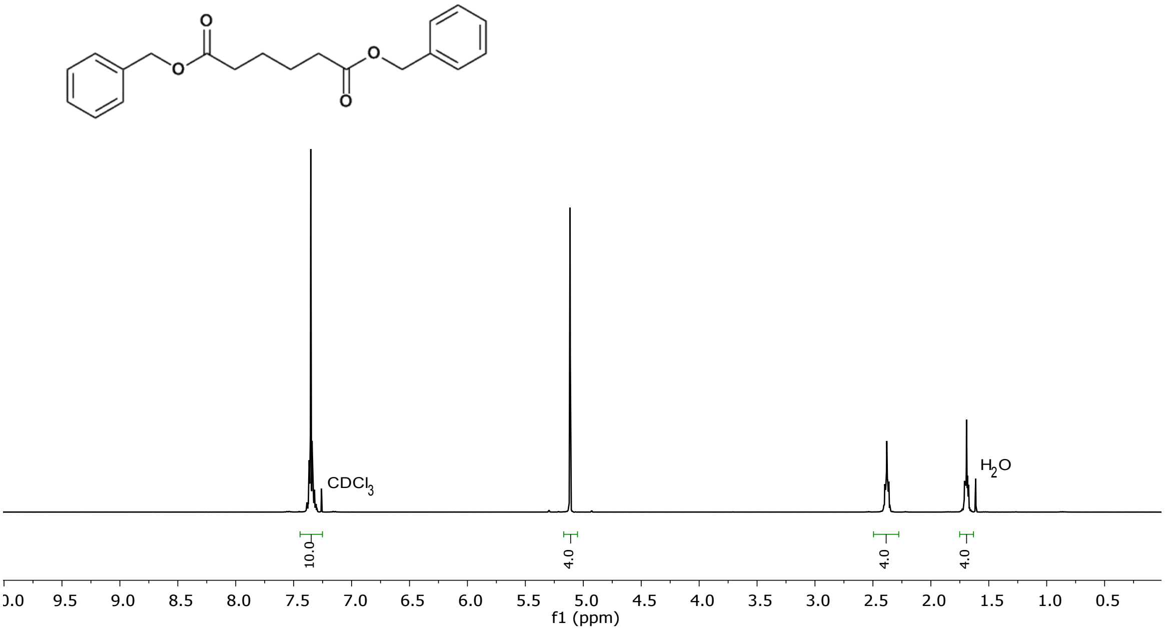

Figure 9. ^{1}H NMR (400 MHz, $CDCl_3$) of dibenzyl adipate.

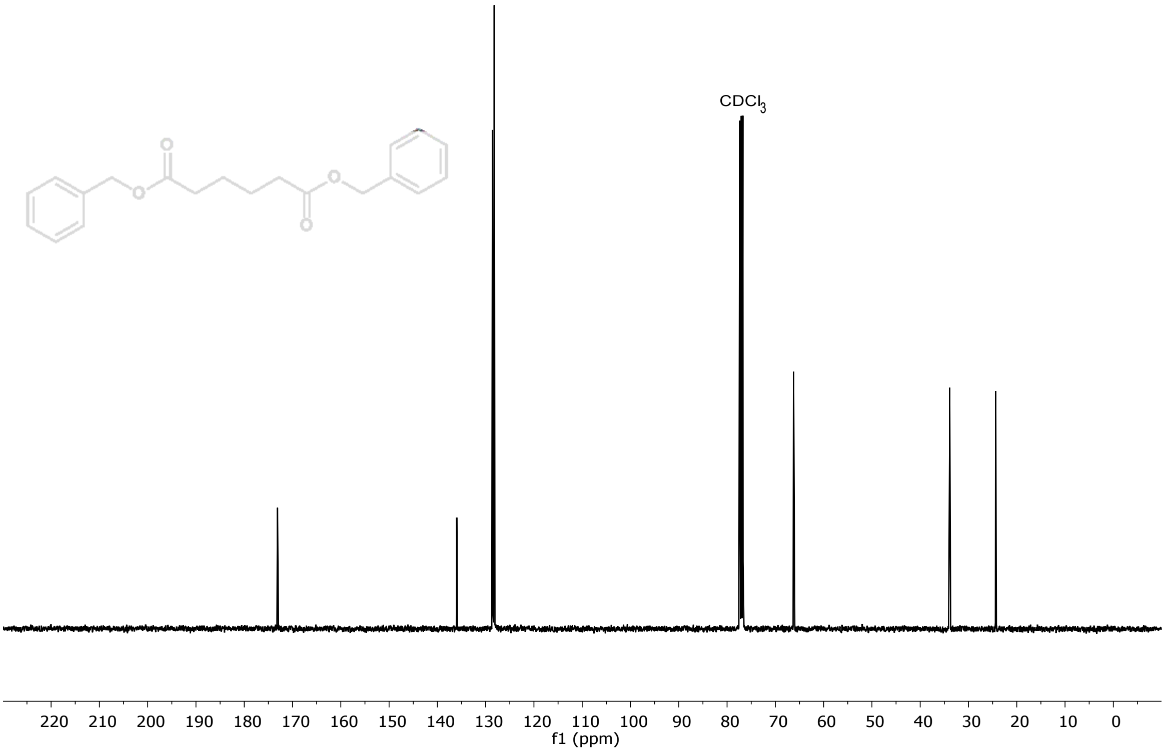

Figure 10. ^{13}C NMR (101 MHz, $CDCl_3$) of dibenzyl adipate.

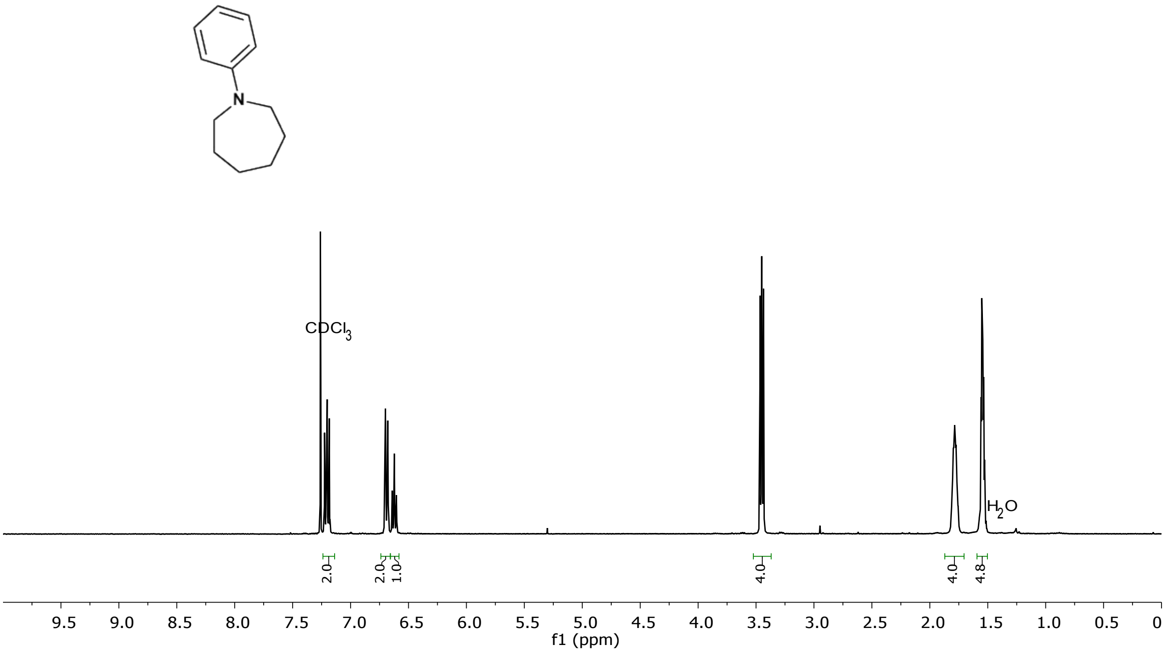

Figure 11. ^{1}H NMR (400 MHz, $CDCl_3$) of *N*-phenylazepane **6**.

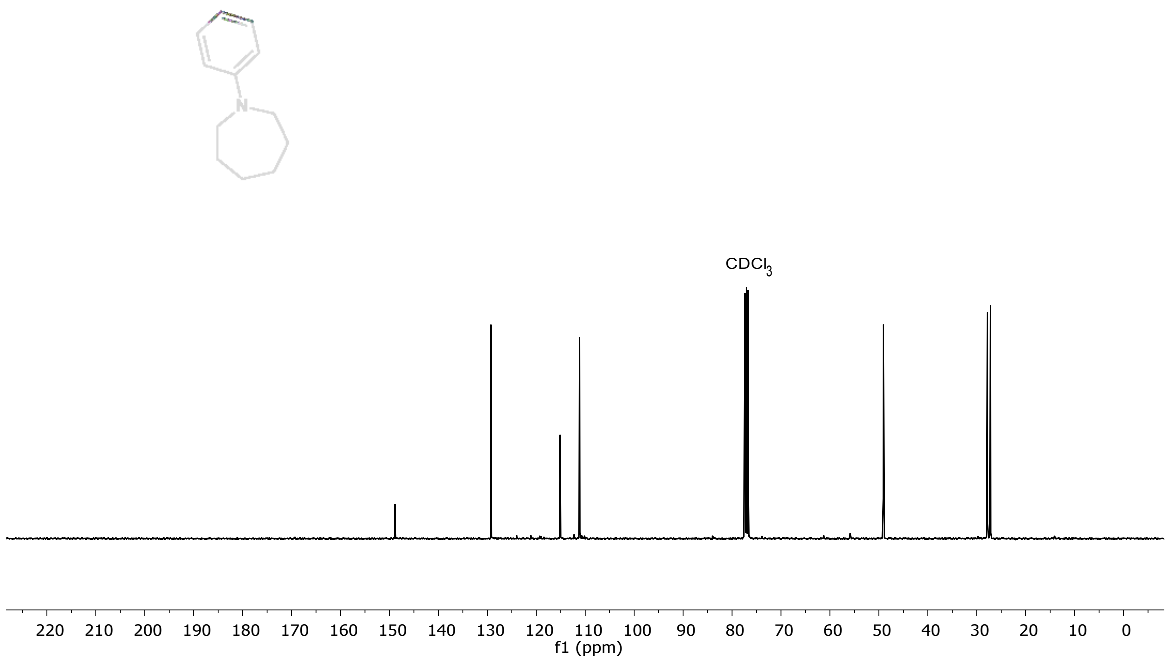

Figure 12. ^{13}C NMR (101 MHz, $CDCl_3$) of *N*-phenylazepane **6**.

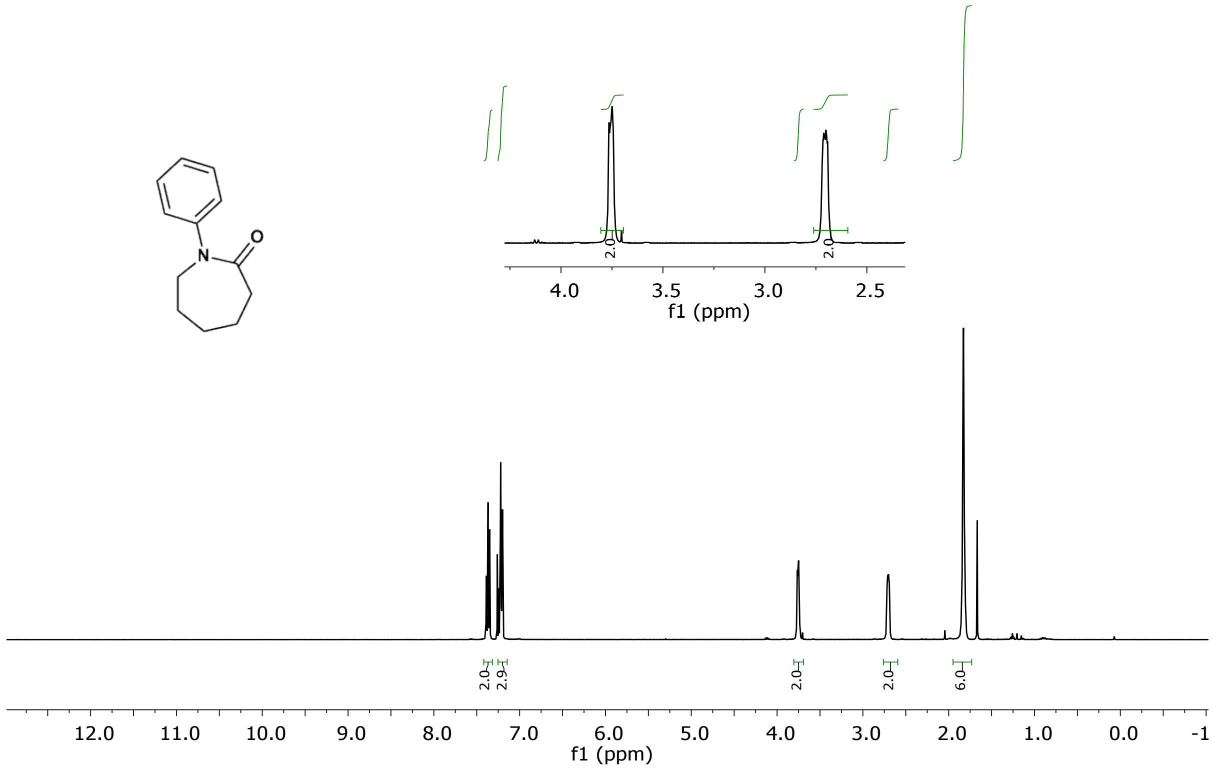

Figure 13. ^{1}H NMR (400 MHz, $CDCl_3$) of *N*-phenyl caprolactam **14**.

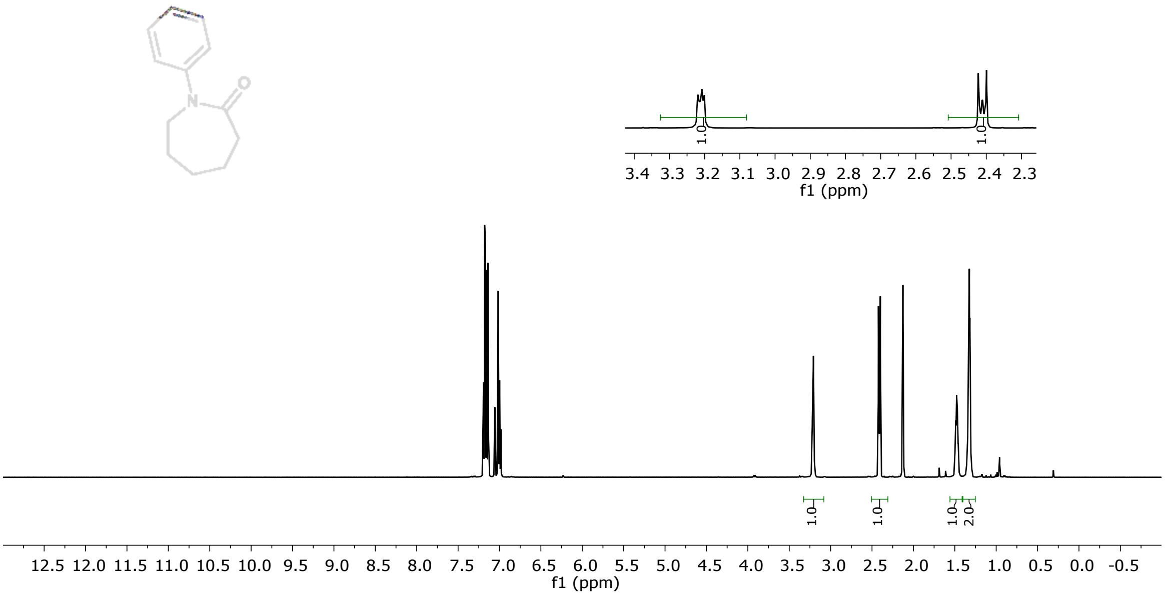

Figure 14. ^{1}H NMR (500 MHz, d_8-toluene, 295 K) of *N*-phenyl caprolactam **14**.

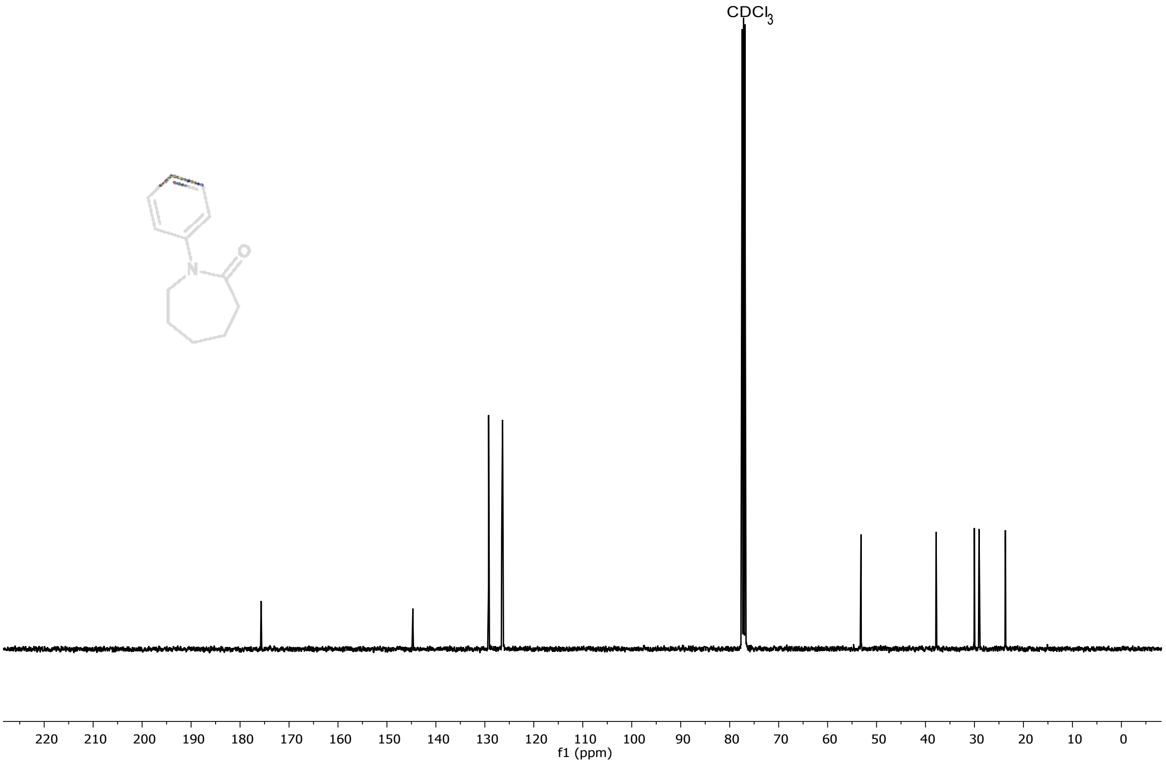

Figure 15. ^{13}C NMR (101 MHz, $CDCl_3$) of *N*-phenyl caprolactam **14**.

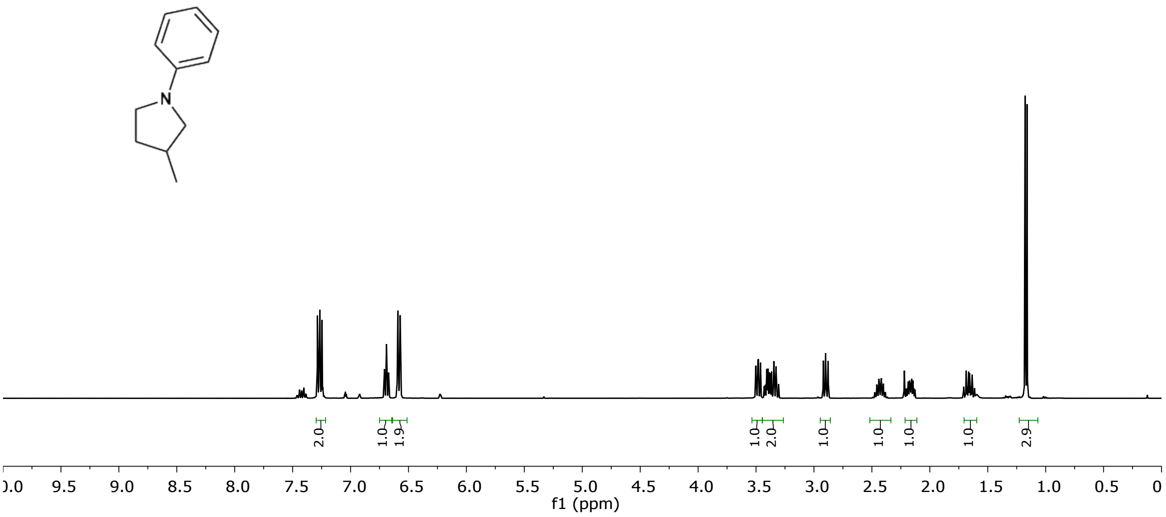

Figure 16. ^{1}H NMR (400 MHz, $CDCl_3$) of *3*-methyl-1-phenylpyrrolidine.

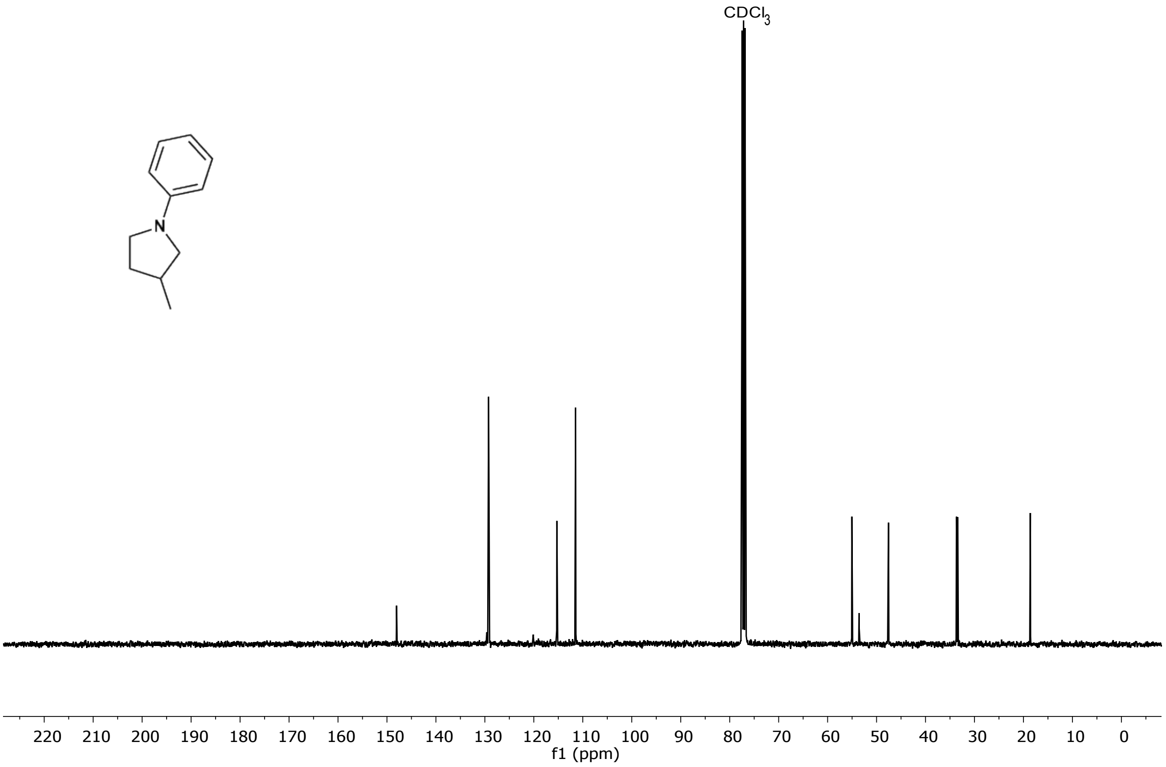

Figure 17. ^{13}C NMR (101 MHz, $CDCl_3$) of *3*-methyl-1-phenylpyrrolidine.

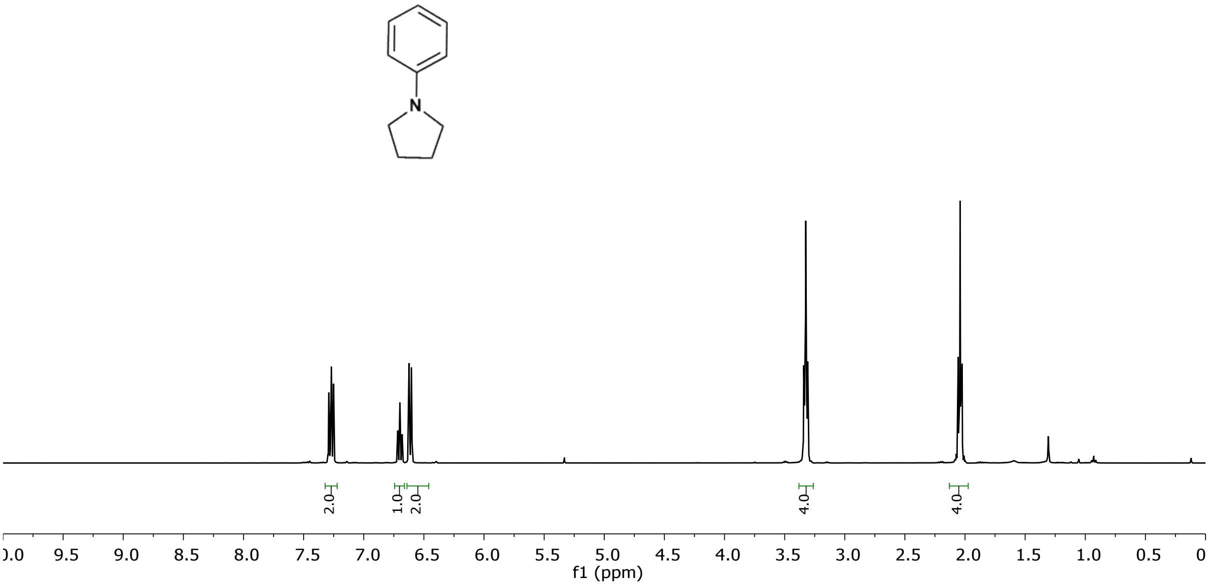

Figure 18. ^{1}H NMR (400 MHz, $CDCl_3$) of *N*-phenylpyrrolidine.

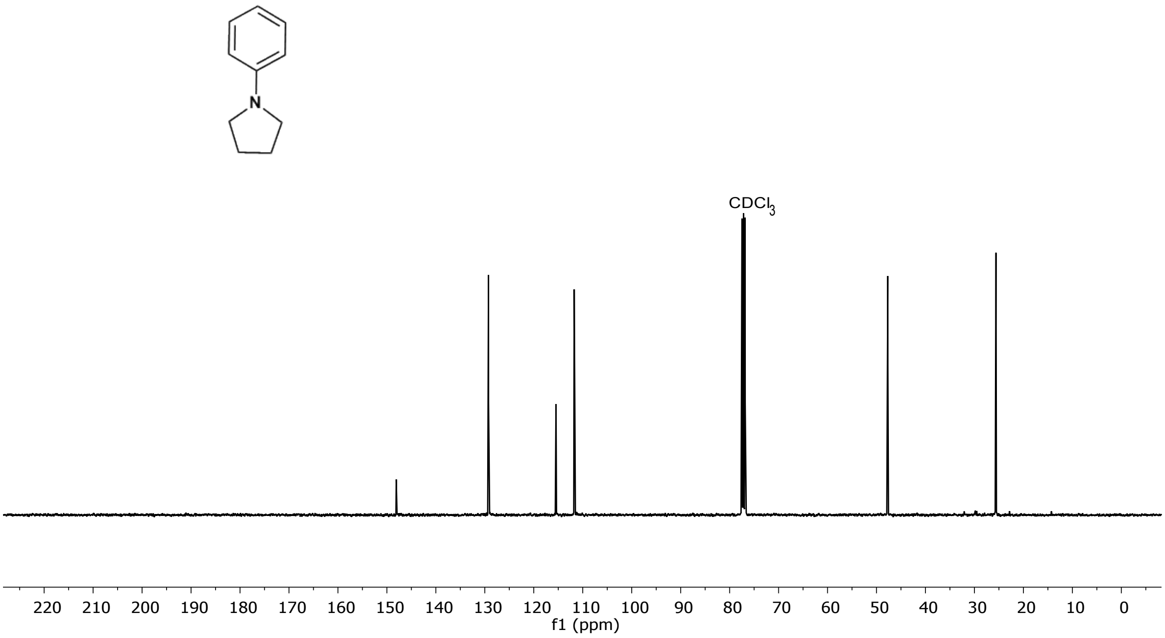

Figure 19. ^{13}C NMR (101 MHz, $CDCl_3$) of *N*-phenylpyrrolidine.

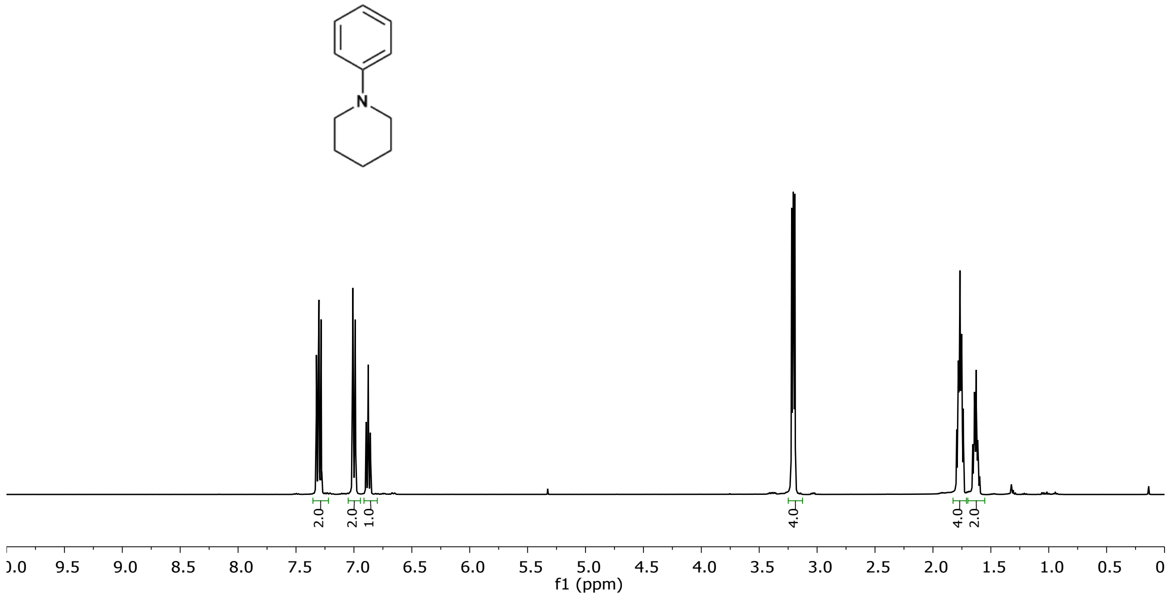

Figure 20. ^{1}H NMR (400 MHz, $CDCl_3$) of *N*-phenylpiperidine.

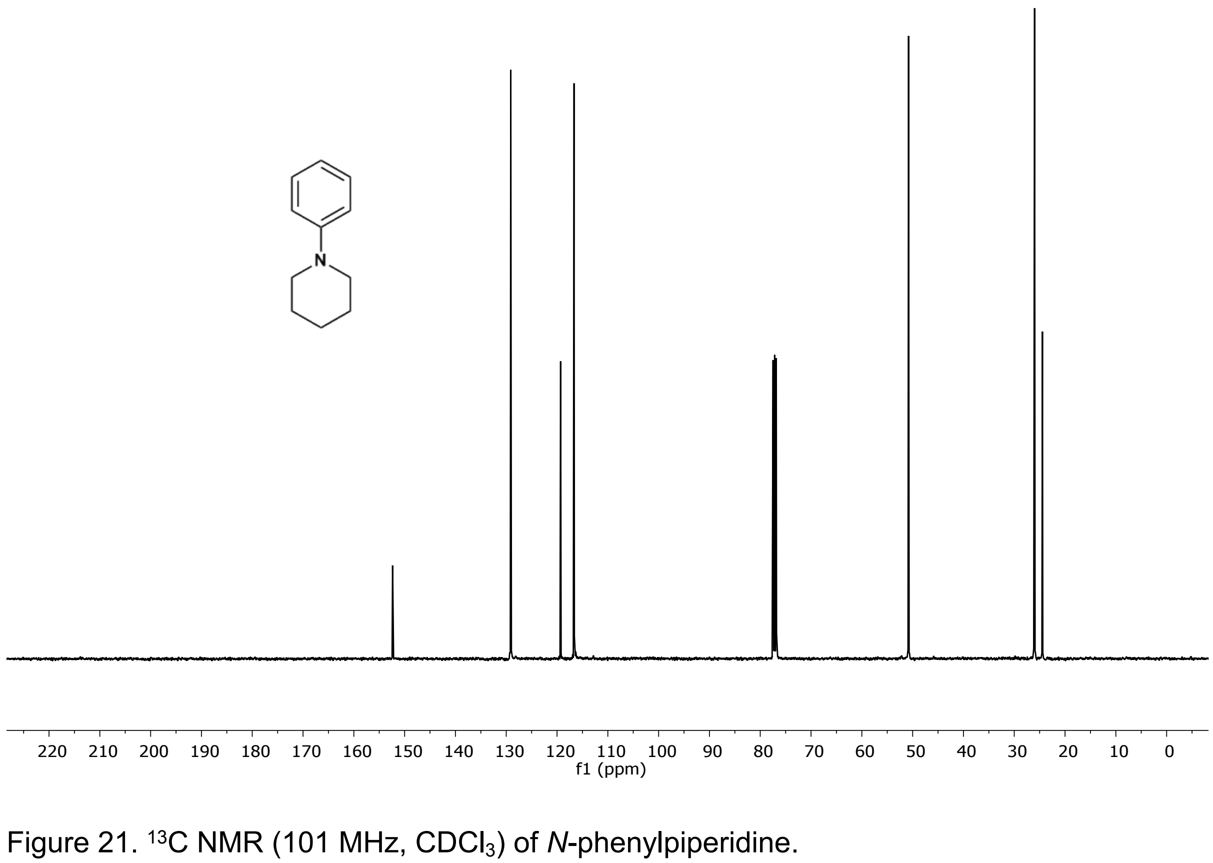

Figure 21. ^{13}C NMR (101 MHz, $CDCl_3$) of *N*-phenylpiperidine.

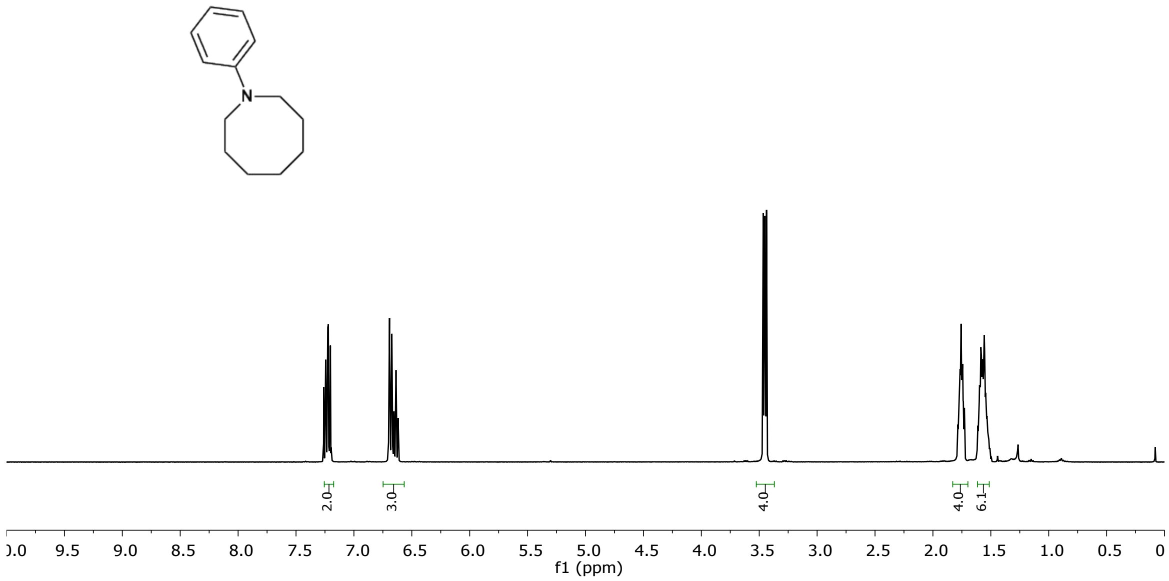

Figure 22. ^{1}H NMR (400 MHz, $CDCl_3$) of *N*-phenylazocane.

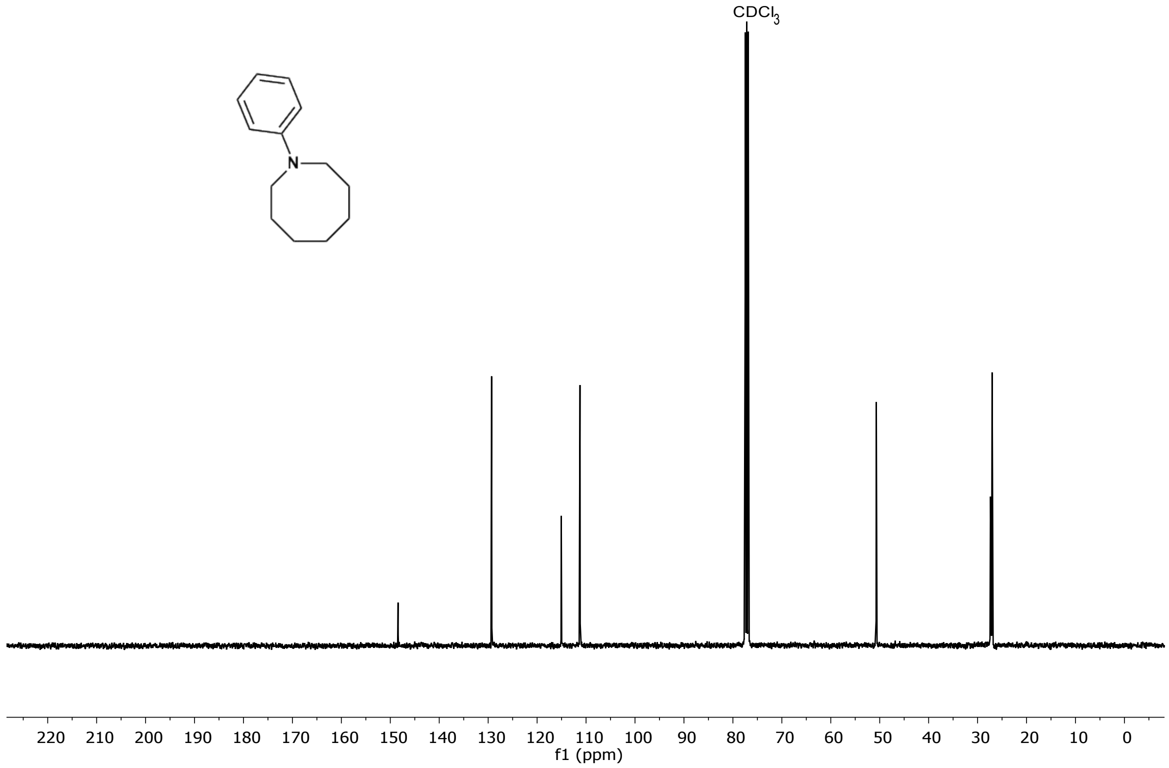

Figure 23. ^{13}C NMR (101 MHz, $CDCl_3$) of *N*-phenylazocane.

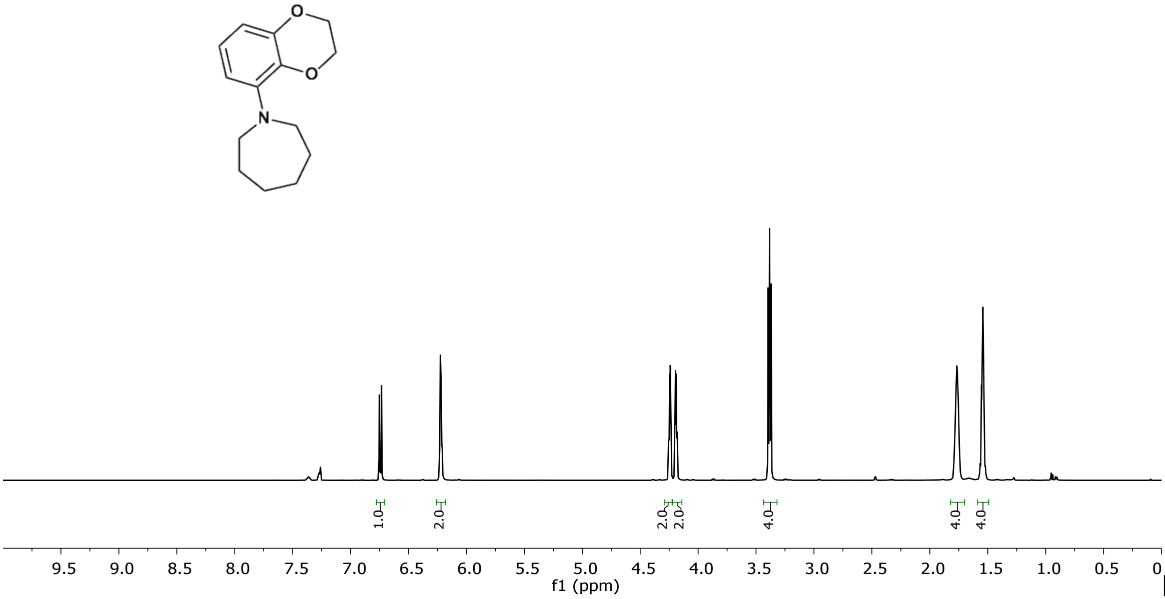

Figure 24. ^{1}H NMR (400 MHz, $CDCl_3$) of *N*-(2,3-dihydrobenzo[1,4]dioxin-5-yl)azepane.

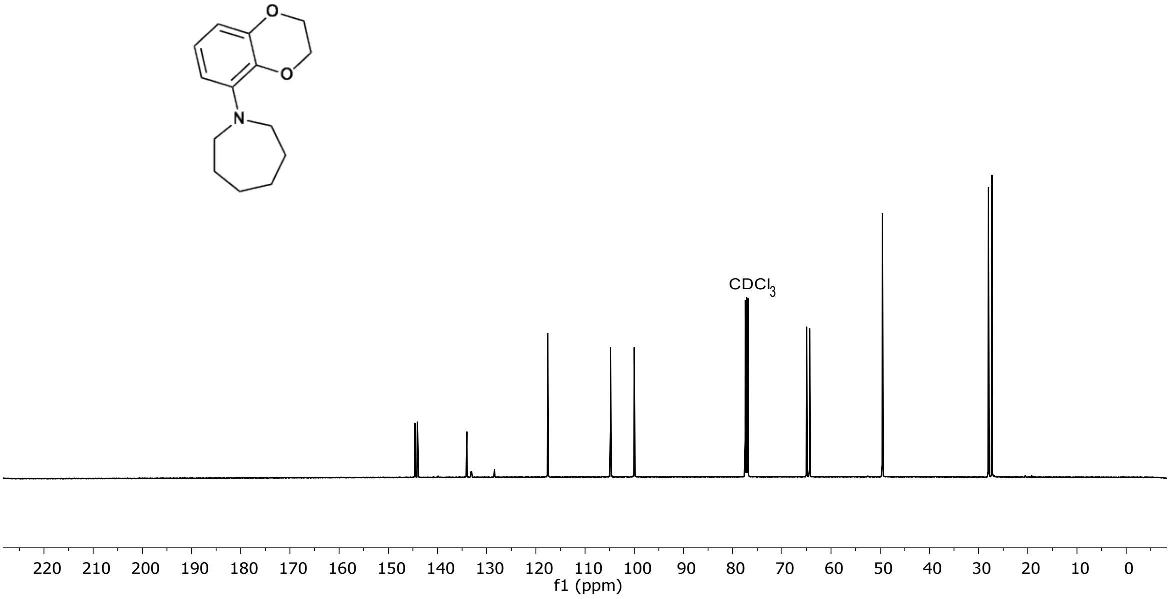

Figure 25. ^{13}C NMR (126 MHz, $CDCl_3$) of *N*-(2,3-dihydrobenzo[1,4]dioxin-5-yl)azepane.

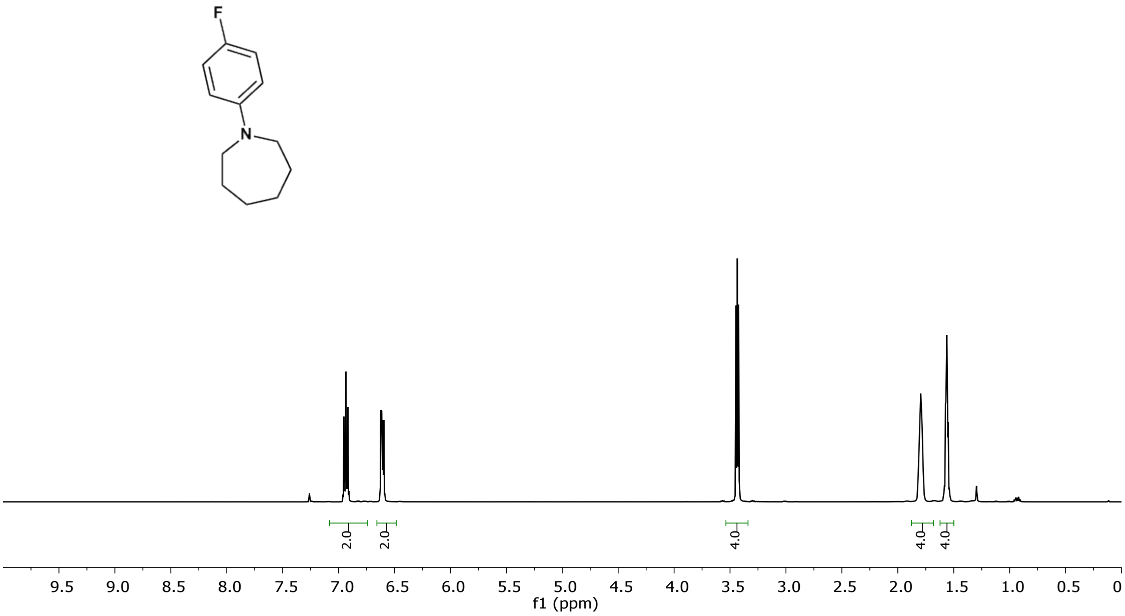

Figure 26. ^{1}H NMR (500 MHz, $CDCl_3$) of *N*-(4-fluorophenyl)azepane.

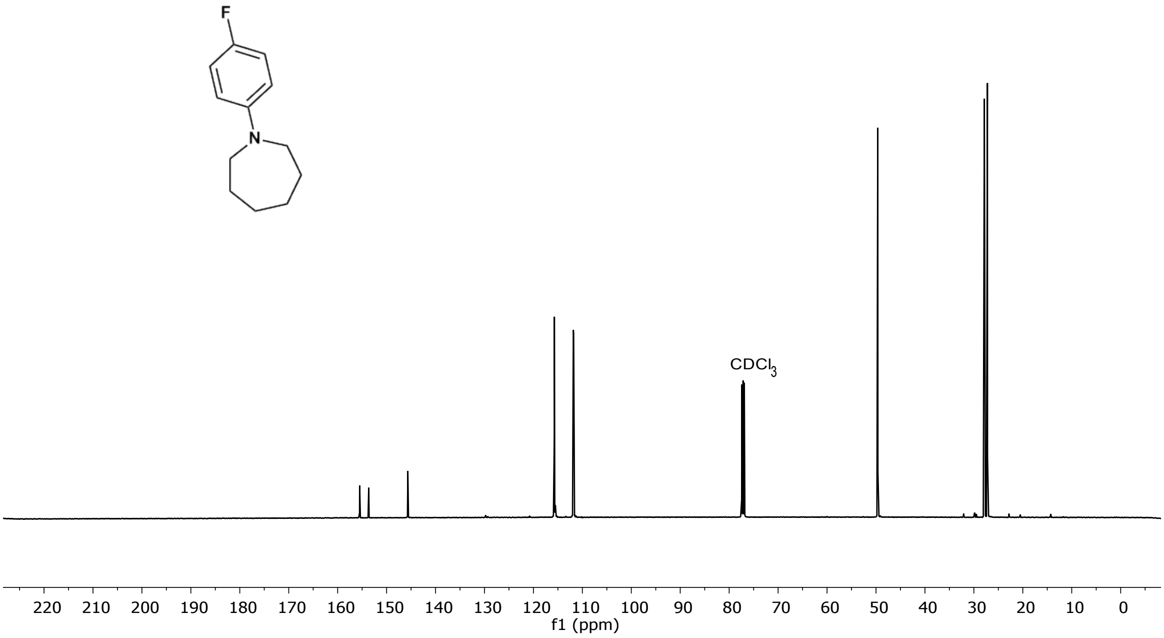

Figure 27. ^{13}C NMR (126 MHz, $CDCl_3$) of *N*-(4-fluorophenyl)azepane.

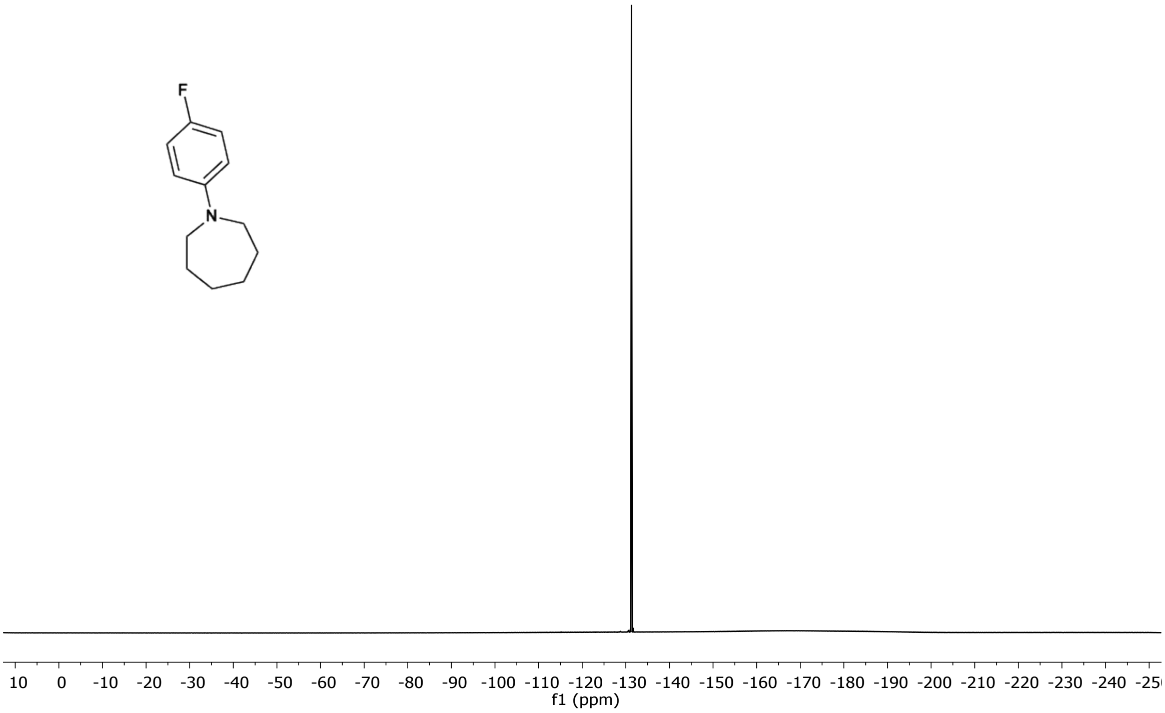

Figure 28. ^{19}F NMR (376 MHz, $CDCl_3$) of *N*-(4-fluorophenyl)azepane.

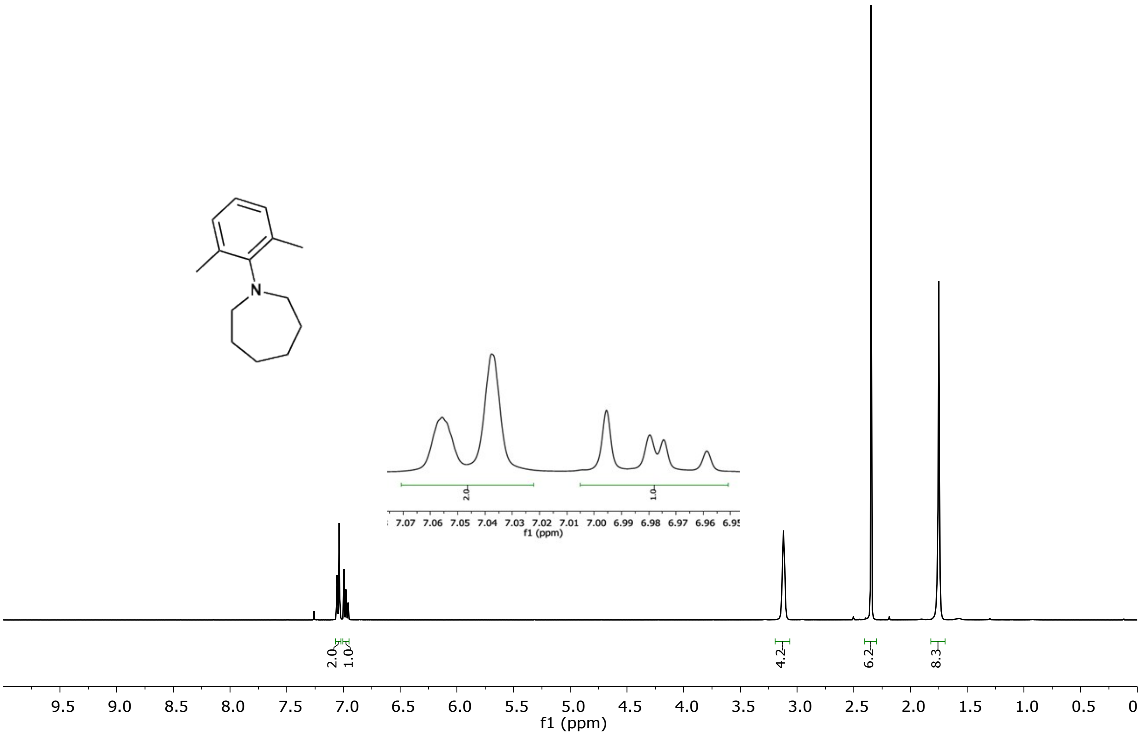

Figure 29. ^{1}H NMR (400 MHz, $CDCl_3$) of *N*-(2,6-dimethylphenyl)azepane. Expandod aromatic region inset

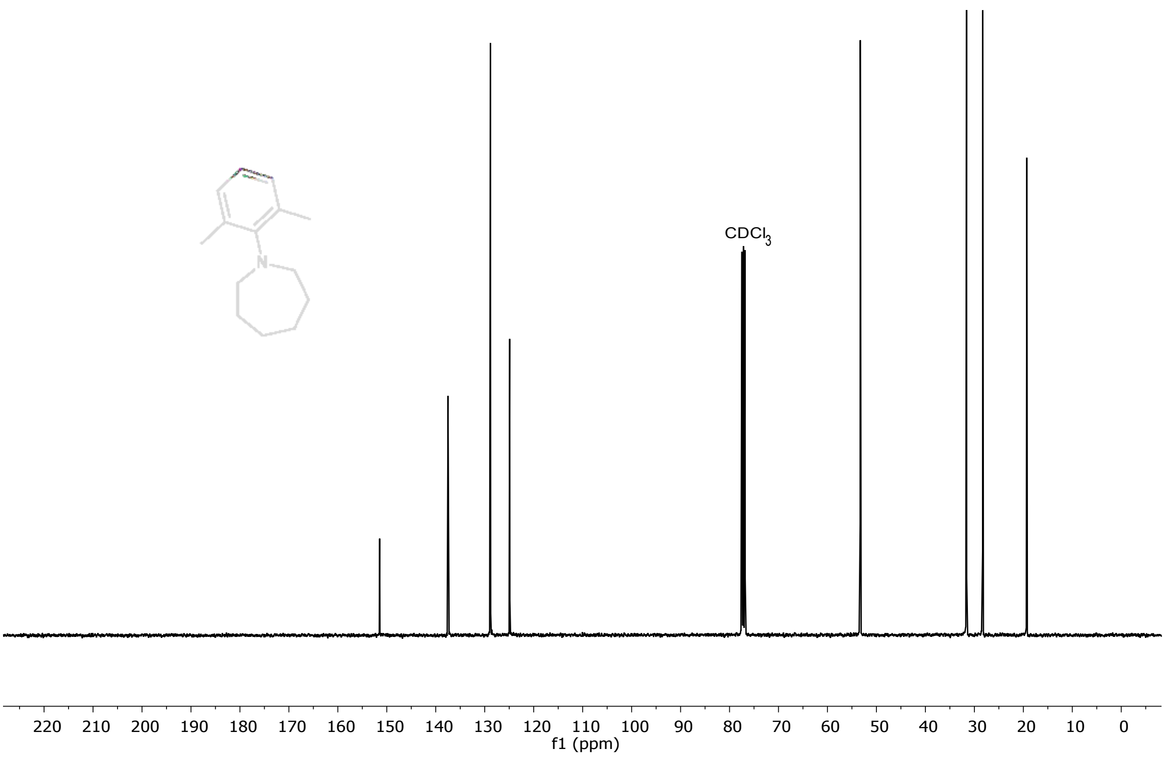

Figure 30. ${}^{13}C$ NMR (101 MHz, $CDCl_3$) of *N*-(2,6-dimethylphenyl)azepane.

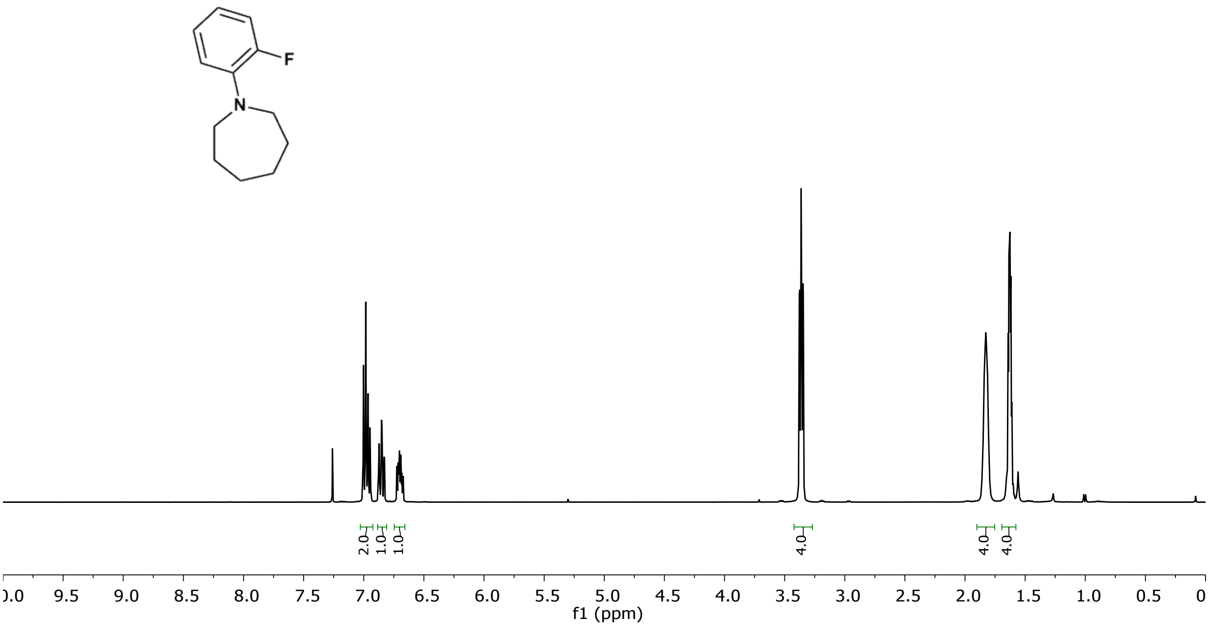

Figure 31. ${}^{1}H$ NMR (400 MHz, $CDCl_3$) of *N*-(2-fluorophenyl)azepane.

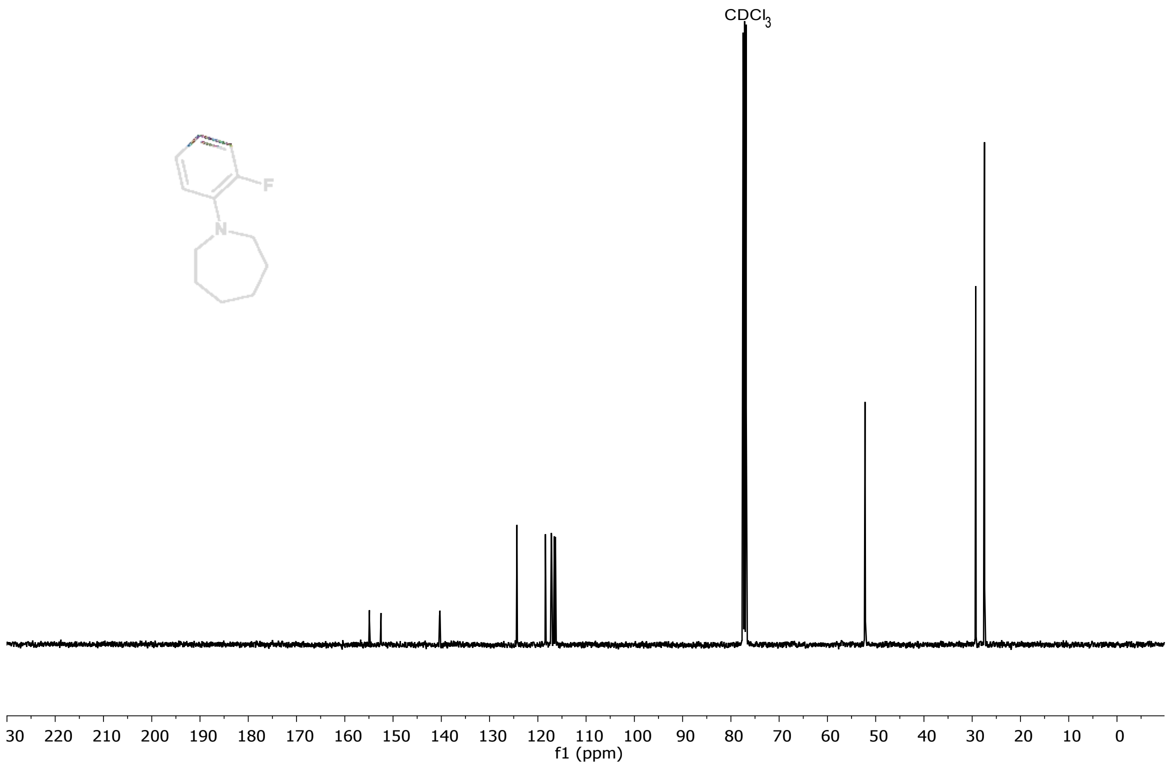

Figure 32. ^{13}C NMR (101 MHz, $CDCl_3$) of *N*-(2-fluorophenyl)azepane.

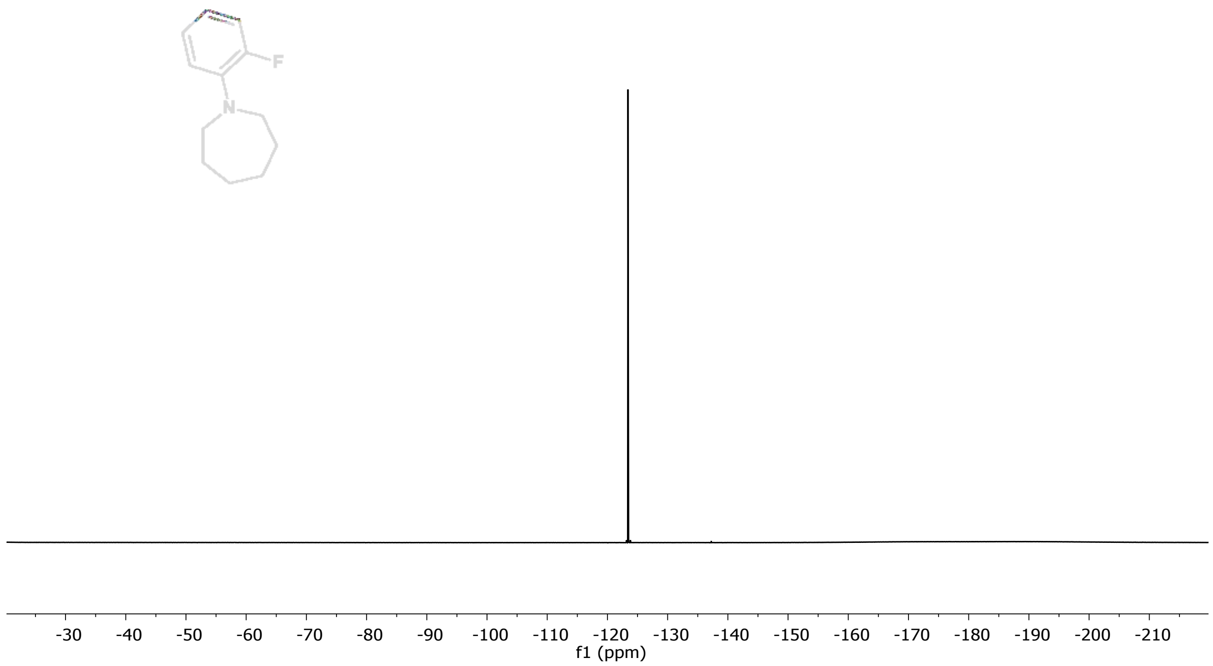

Figure 33. ^{19}F NMR (377 MHz, $CDCl_3$) of *N*-(2-fluorophenyl)azepane.

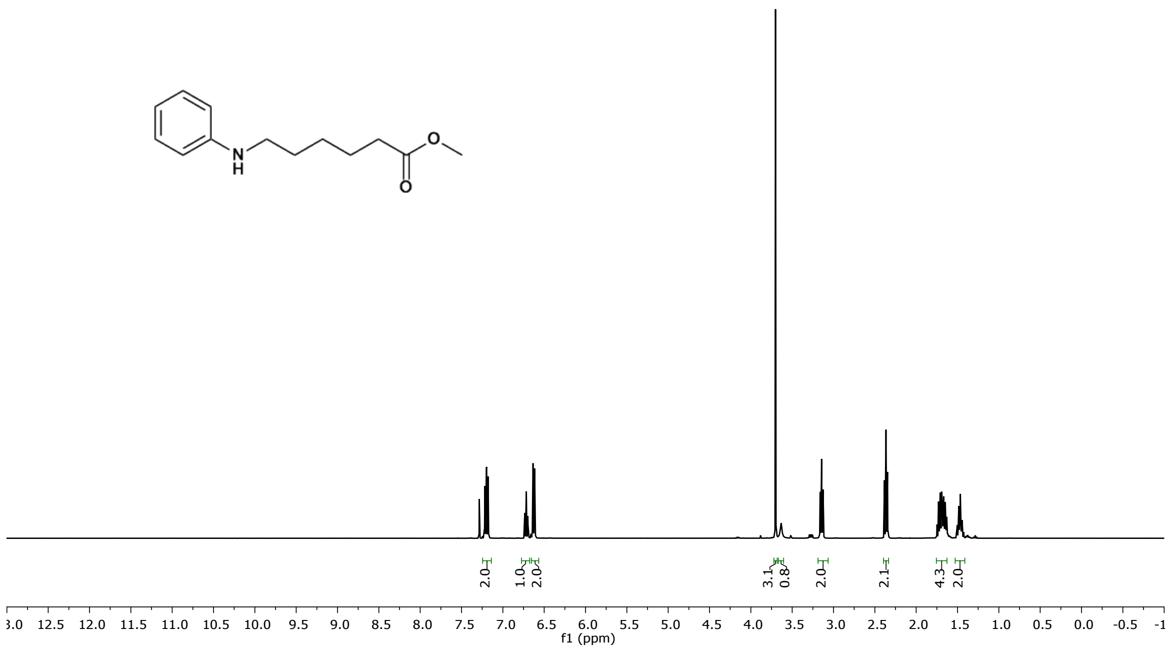

Figure 34. ^{1}H NMR (400 MHz, $CDCl_3$) of methyl 6-(phenylamino)hexanoate **12**.

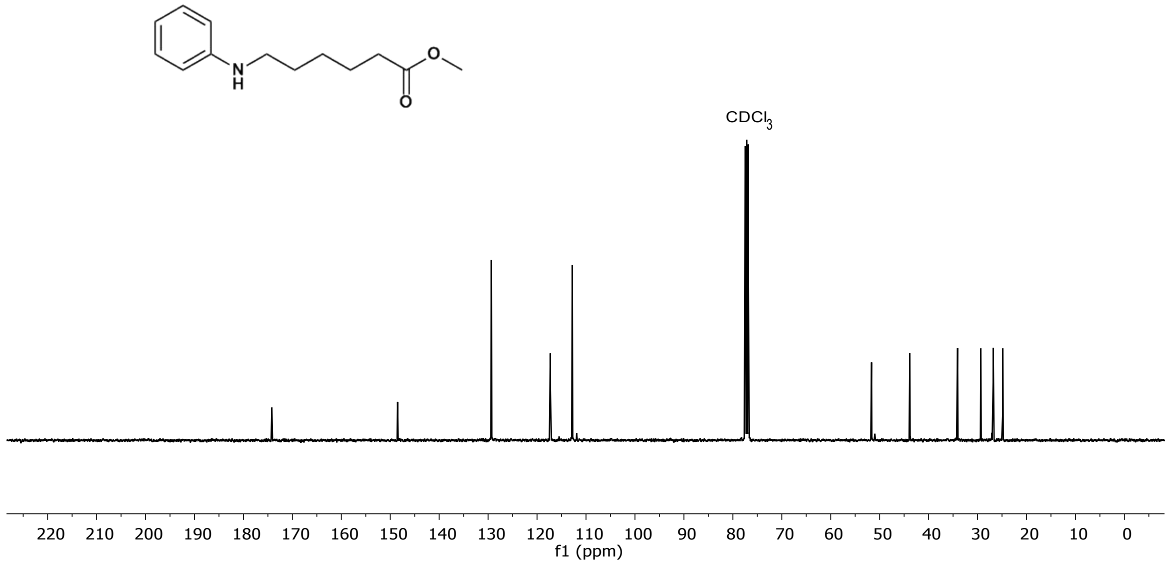

Figure 35. ^{13}C NMR (101 MHz, $CDCl_3$) of methyl 6-(phenylamino)hexanoate **12**.

4. Analysis of reaction mixture:

4.1. Representative NMR spectra of reaction mixtures

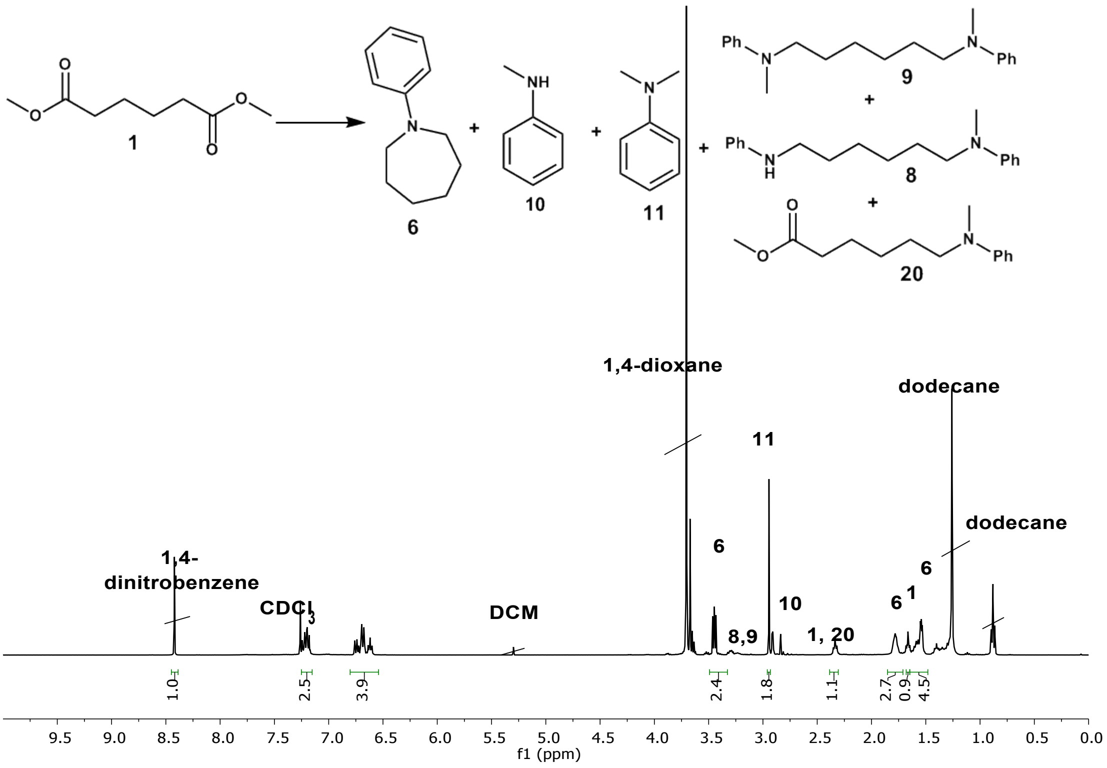

Figure 36. ^{1}H NMR of reaction mixture using dimethyl adipate. Dodecane was used as the internal standard for quantitative GC analysis.1,4-Dinitrobenzene (0.5 mmol) was used as the internal standard for quantitative NMR analysis.

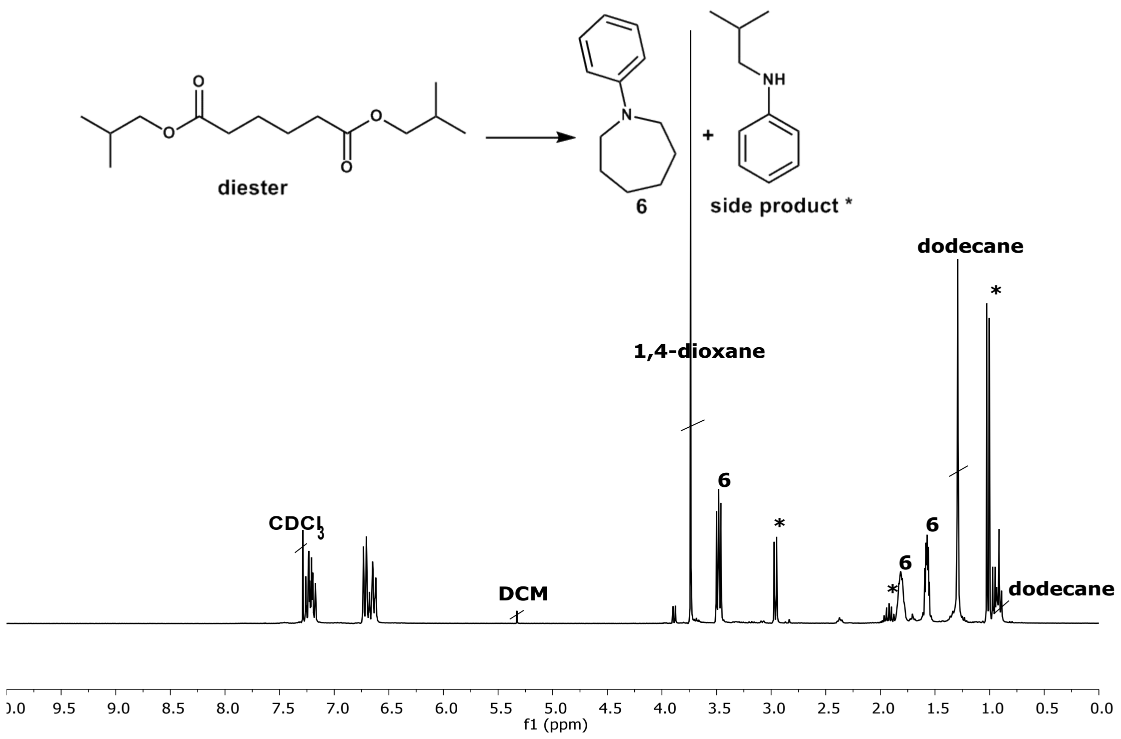

Figure 37. ^{1}H NMR of reaction mixture using diisobutyl adipate. Dodecane was used as the internal standard for quantitative GC analysis.

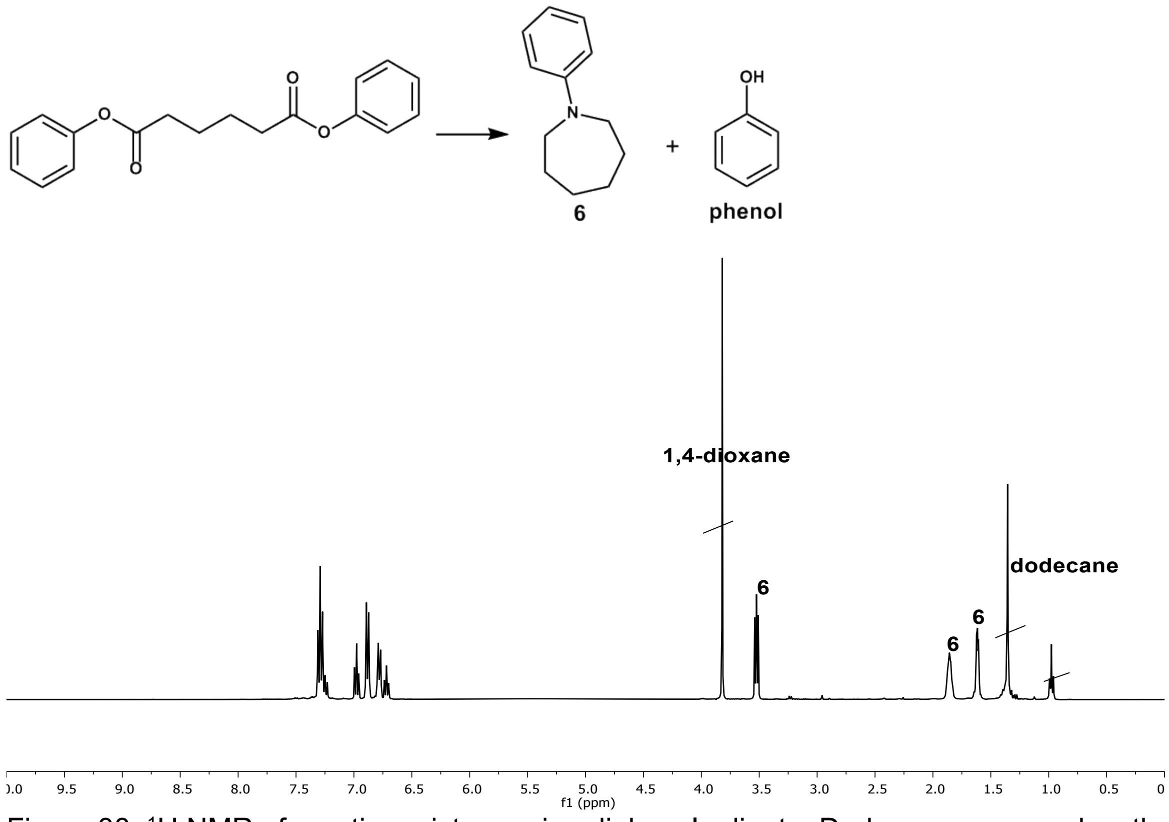

Figure 38. ^{1}H NMR of reaction mixture using diphenyl adipate. Dodecane was used as the internal standard for quantitative GC analysis.

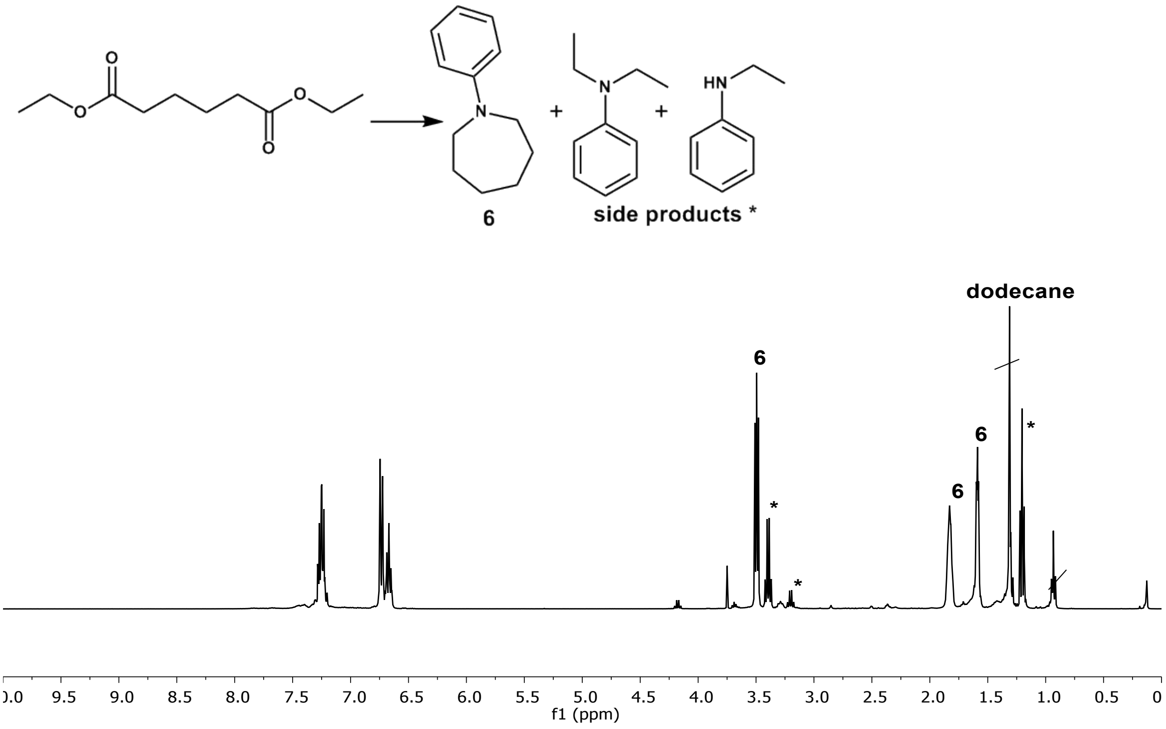

Figure 39. ^{1}H NMR of reaction mixture using diethyl adipate. Dodecane was used as the internal standard for quantitative GC analysis.

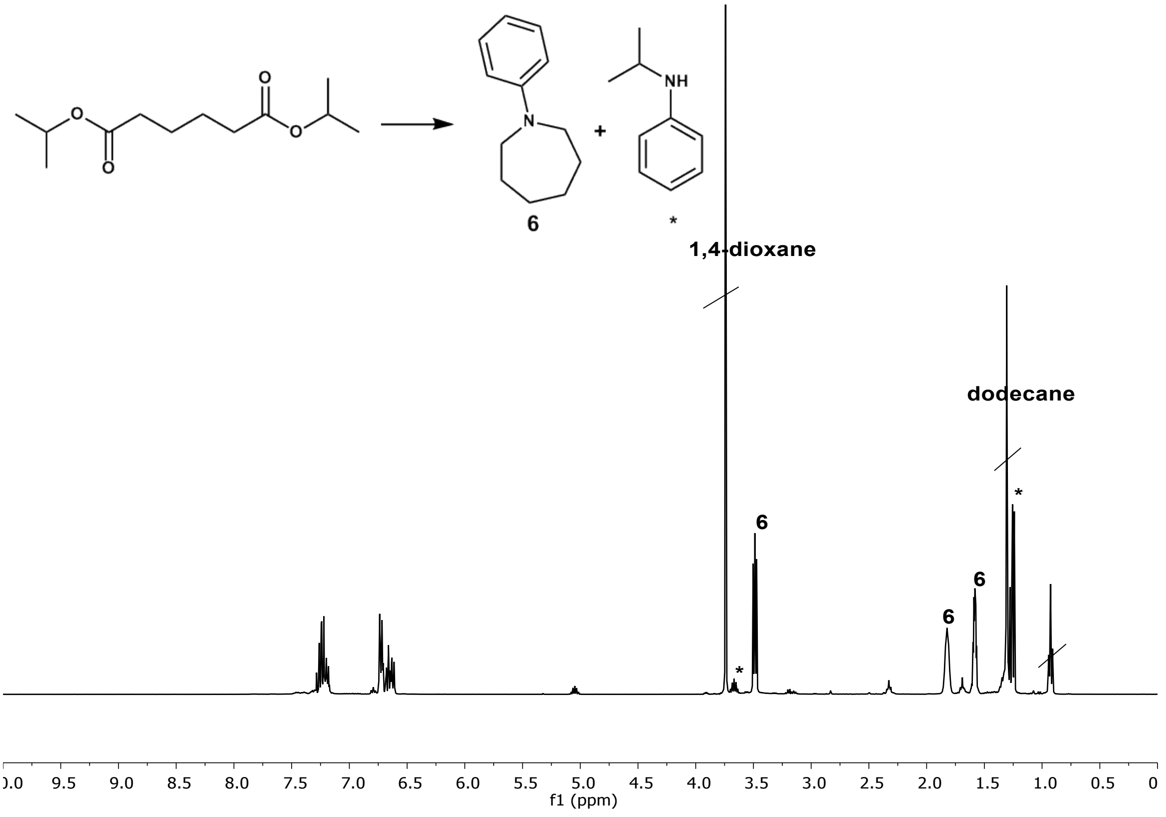

Figure 40. ^{1}H NMR of reaction mixture using diisopropyl adipate. Dodecane was used as the internal standard for quantitative GC analysis.

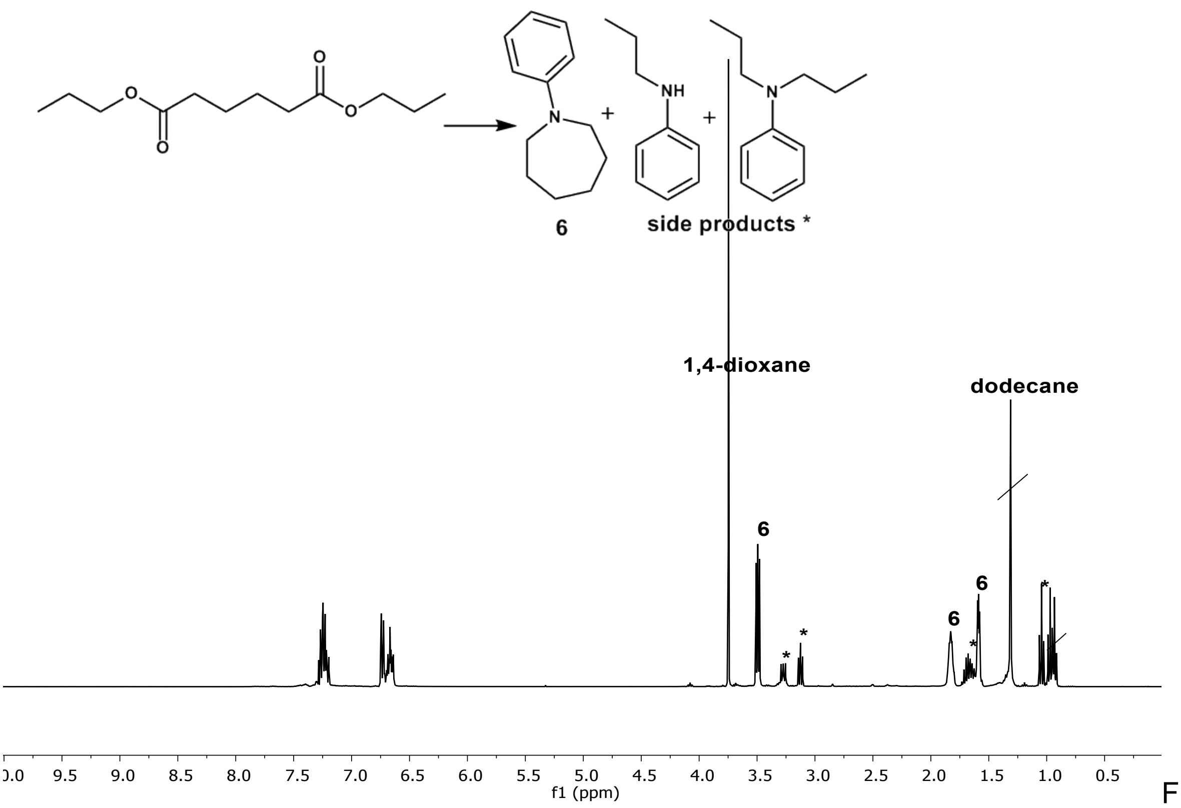

Figure 41. ^{1}H NMR of reaction mixture using di-*n*-propyl adipate. Dodecane was used as the internal standard for quantitative GC analysis.

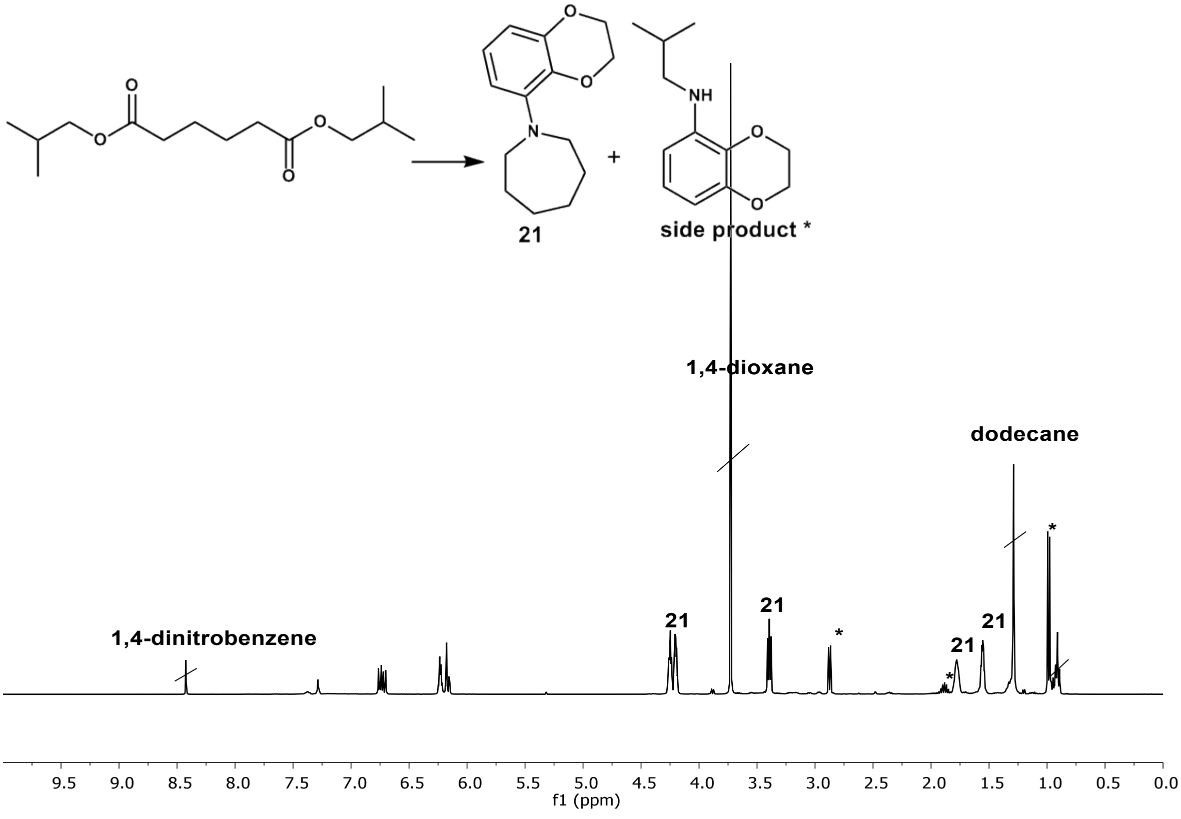

Figure 42. ^{1}H NMR of reaction mixture using diisobutyl adipate and 1,4-benzodioxan-6-amine. Dodecane was used as the internal standard for quantitative GC analysis. 1,4-Dinitrobenzene was used as the internal standard for quantitative NMR analysis.

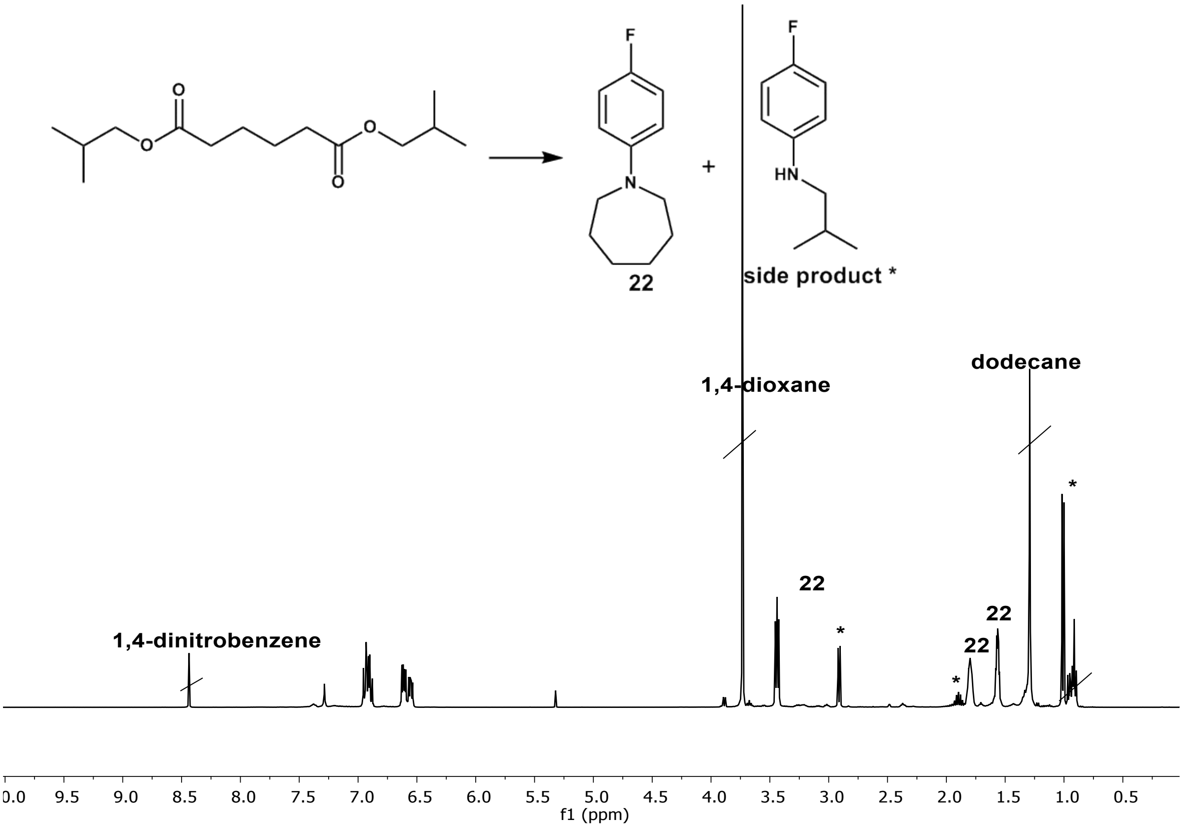

Figure 43. ^{1}H NMR of reaction mixture using diisobutyl adipate and 4-fluoroaniline. Dodecane was used as the internal standard for quantitative GC analysis. 1,4-Dinitrobenzene was used as the internal standard for quantitative NMR analysis.

4.2. Representative GC spectra of reaction mixture

Injection mode	Split
Split ratio	79
Carrier gas	He
Flow control	Flow rate
Flow rate	1.4 mL min^{-1}
Oven temperature programme	50°C(4 min), 50 - 130°C at 20 °C min^{-1}, then hold 2 min, then 130 – 220°C at 20 °C min^{-1}, then hold 15.5 min
Column type	RXi®– 35ms
Column dimensions	30 m x 0.25 mm x 0. 5 µm
Detector type	Flame Ionisation Detector
Detector temp	220°C

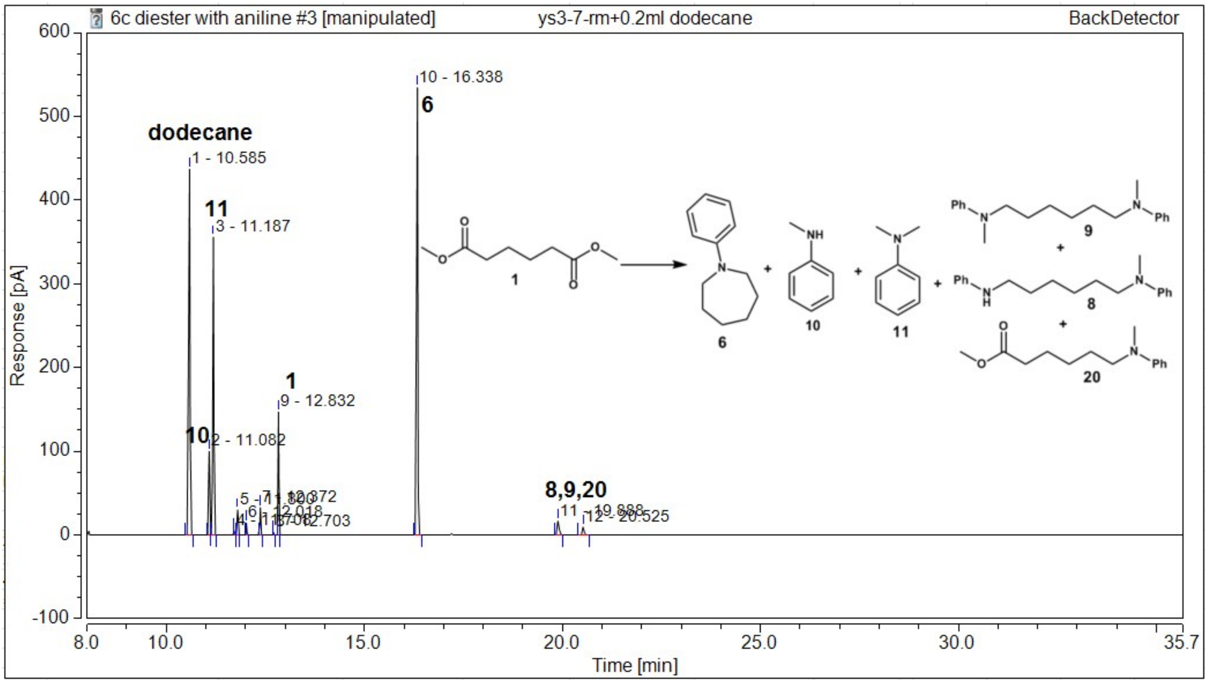

Figure 44. GC spectrum of reaction mixture using dimethyl adipate. Dodecane was used as the internal standard for quantitative GC analysis.

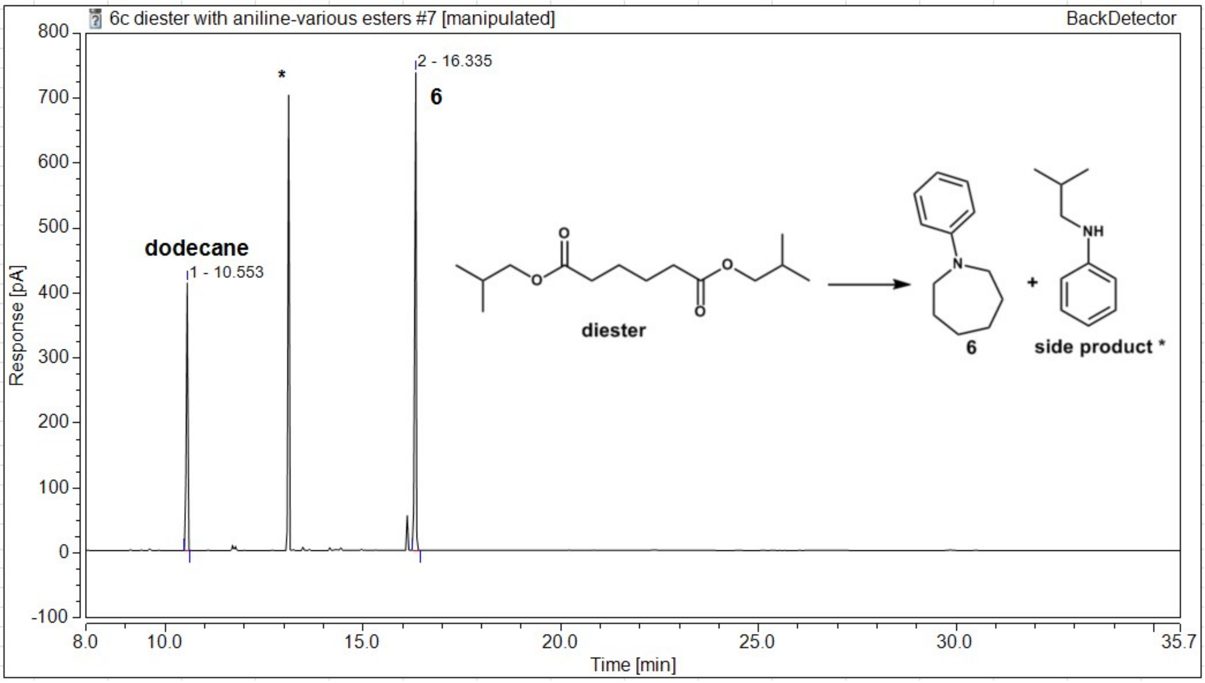

Figure 45. GC spectrum of reaction mixture using diisobutyl adipate. Dodecane was used as the internal standard for quantitative GC analysis.

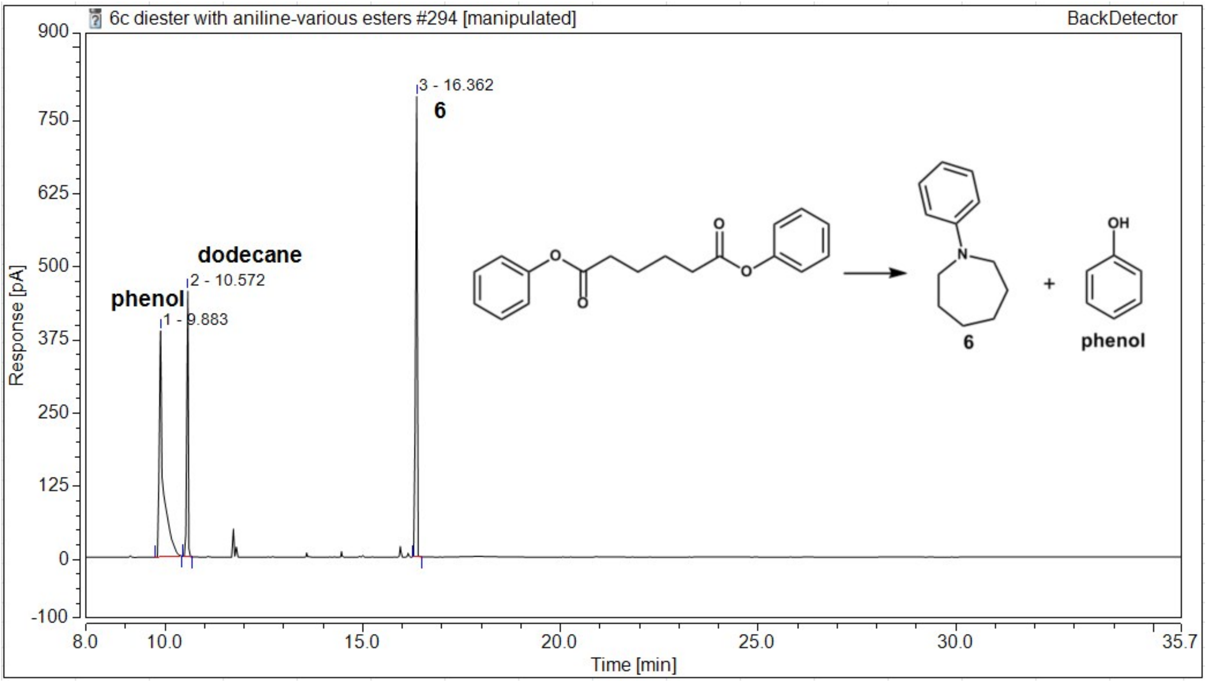

Figure 46. GC spectrum of reaction mixture using diphenyl adipate. Dodecane was used as the internal standard for quantitative GC analysis.

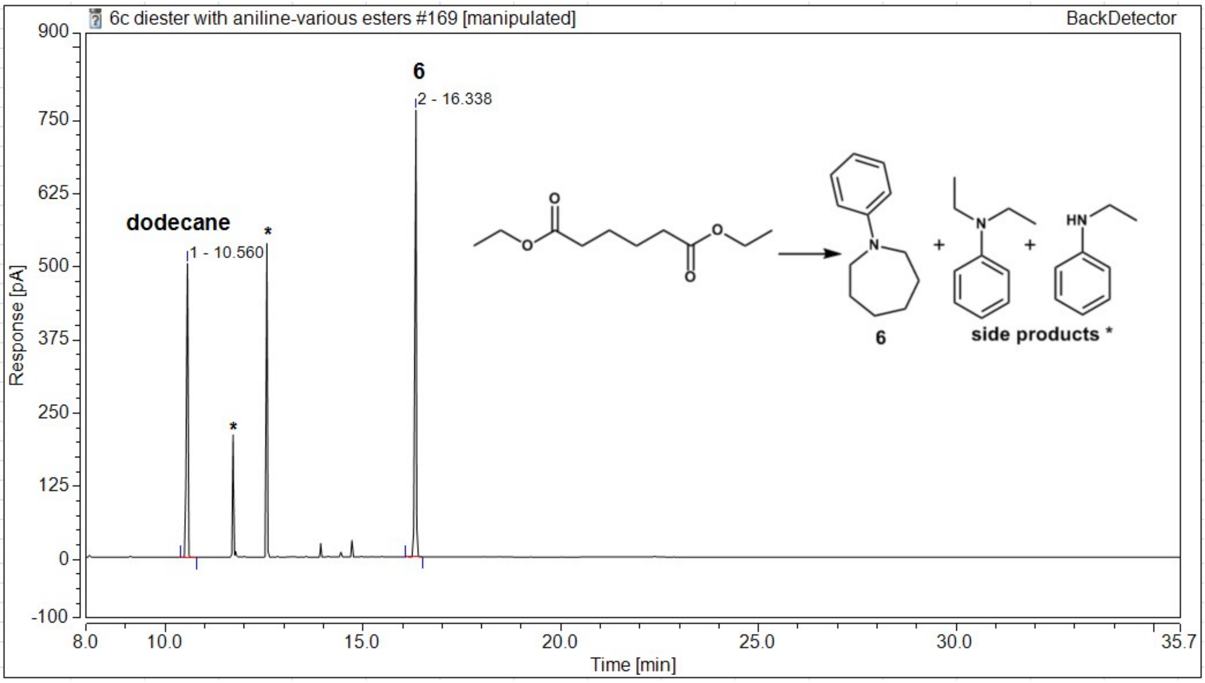

Figure 47. GC spectrum of reaction mixture using diethyl adipate. Dodecane was used as the internal standard for quantitative GC analysis.

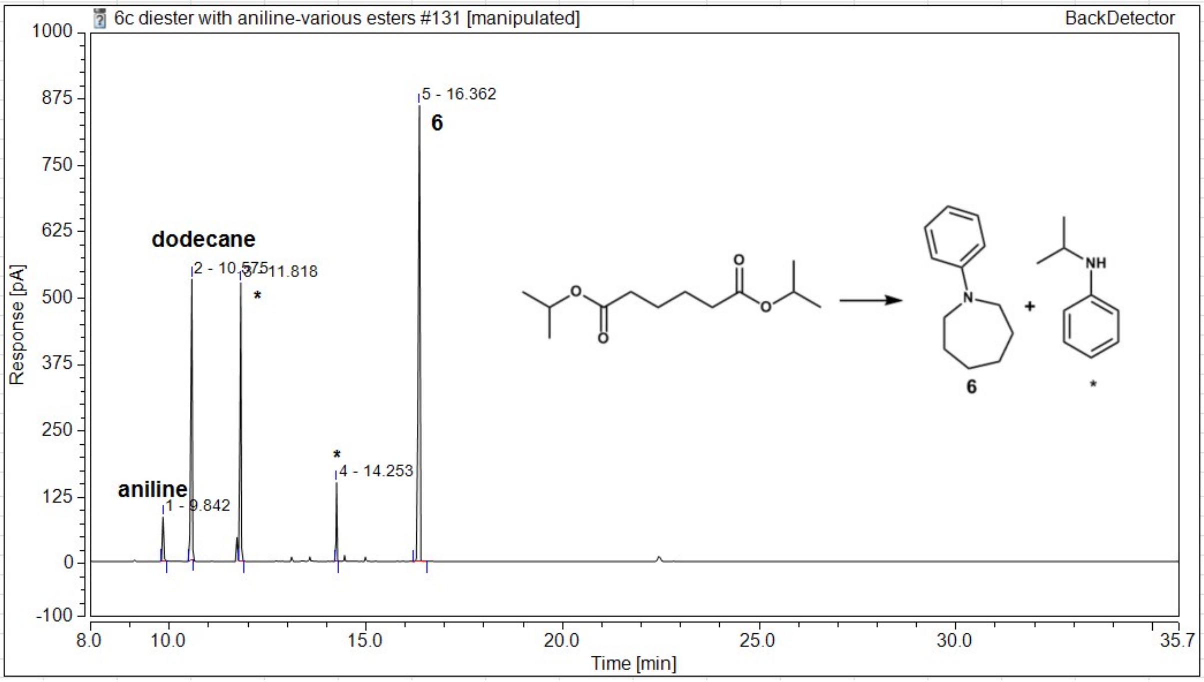

Figure 48. GC spectrum of reaction mixture using diisopropyl adipate. Dodecane was used as the internal standard for quantitative GC analysis.

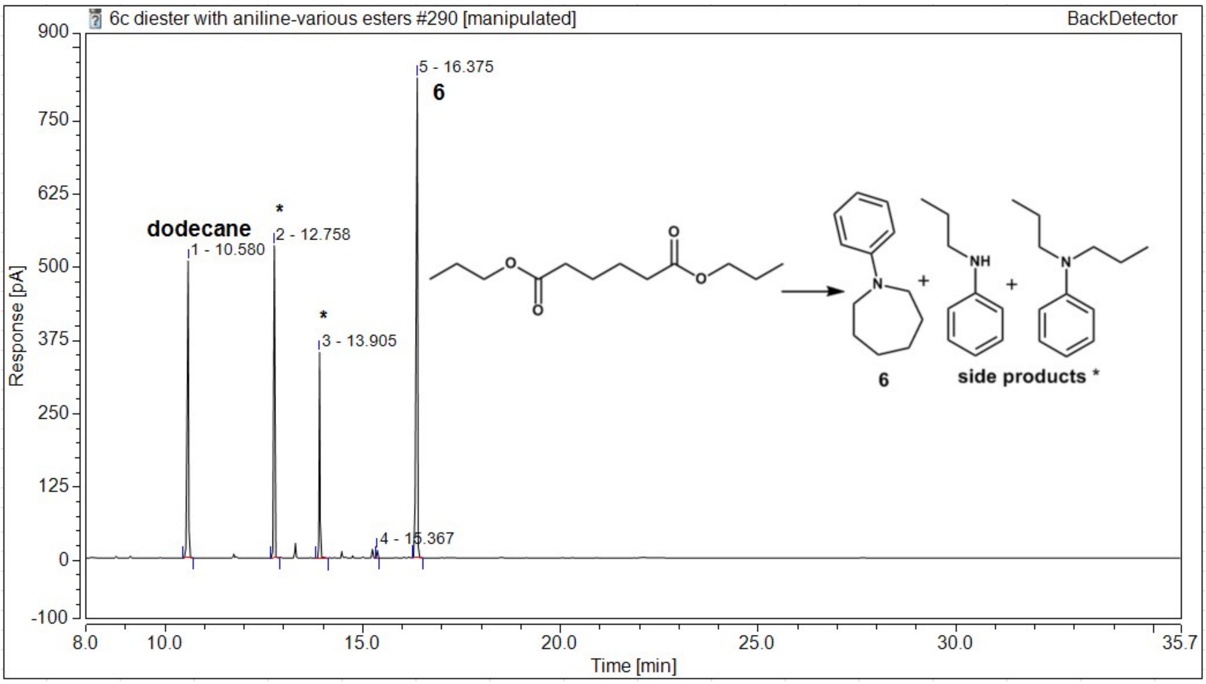

Figure 49. GC spectrum of reaction mixture using di-*n*-propyl adipate. Dodecane was used as the internal standard for quantitative GC analysis.

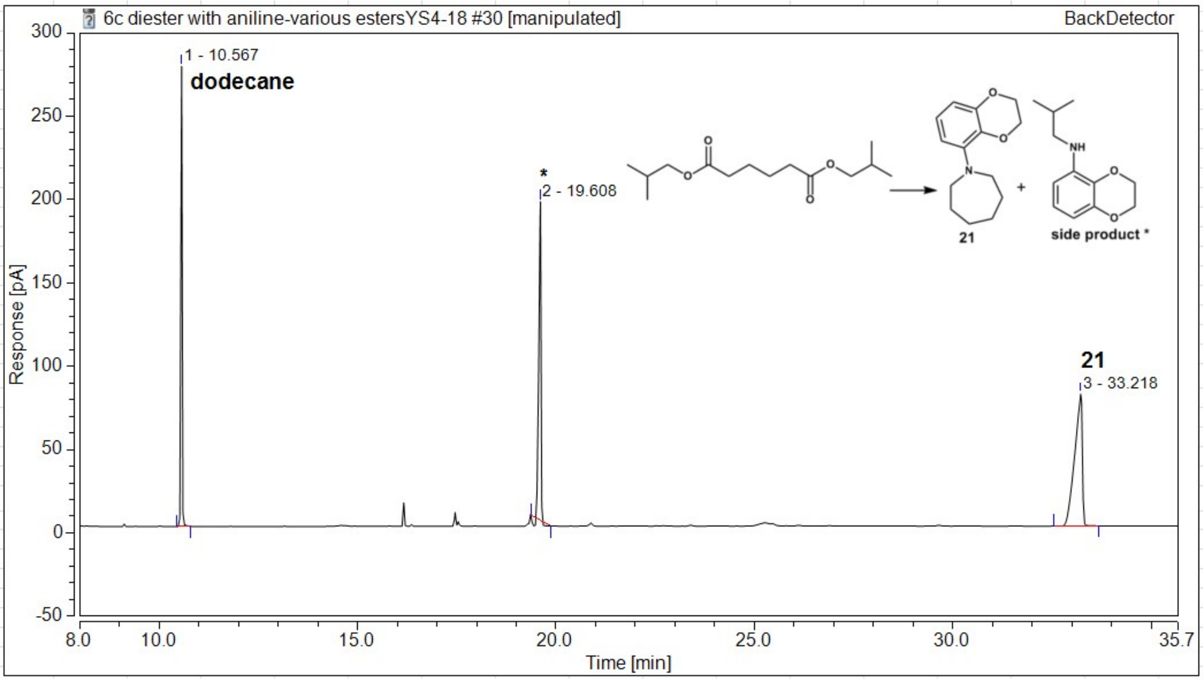

Figure 50. GC spectrum of reaction mixture using diisobutyl adipate and 1,4-benzodioxan-6-amine. Dodecane was used as the internal standard for quantitative GC analysis.

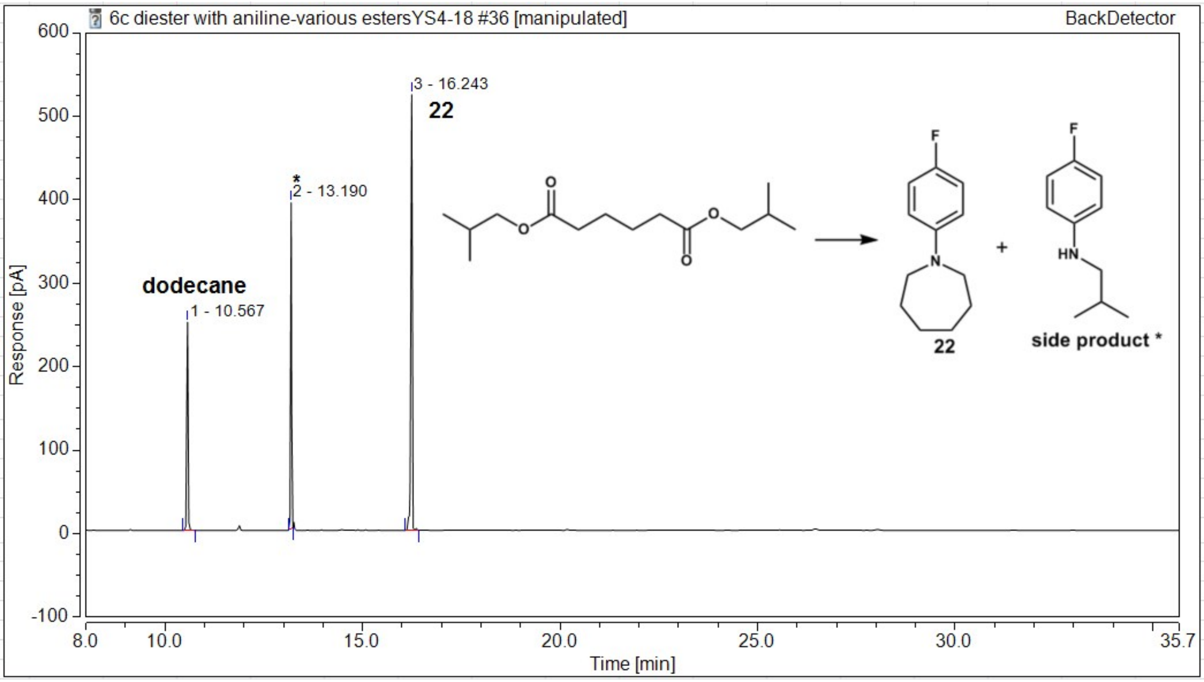

Figure 51. GC spectrum of reaction mixture using diisobutyl adipate and 4-fluoroaniline. Dodecane was used as the internal standard for quantitative GC analysis.

4.3. GC spectra of reaction mixtures starting from enantiopure substrates using a chiral column

Injection mode	Split
Split ratio	200
Carrier gas	H_2
Flow control	Flow rate
Flow rate	2 mL min^{-1}
Oven temperature programme	90 – 130°C at 2 °C min^{-1}, hold 5 min
Column type	Beta DEX™ 225
Column dimensions	30 m x 0.25 mm x 0. 25 µm
Detector type	Flame Ionisation Detector
Detector temp	220°C

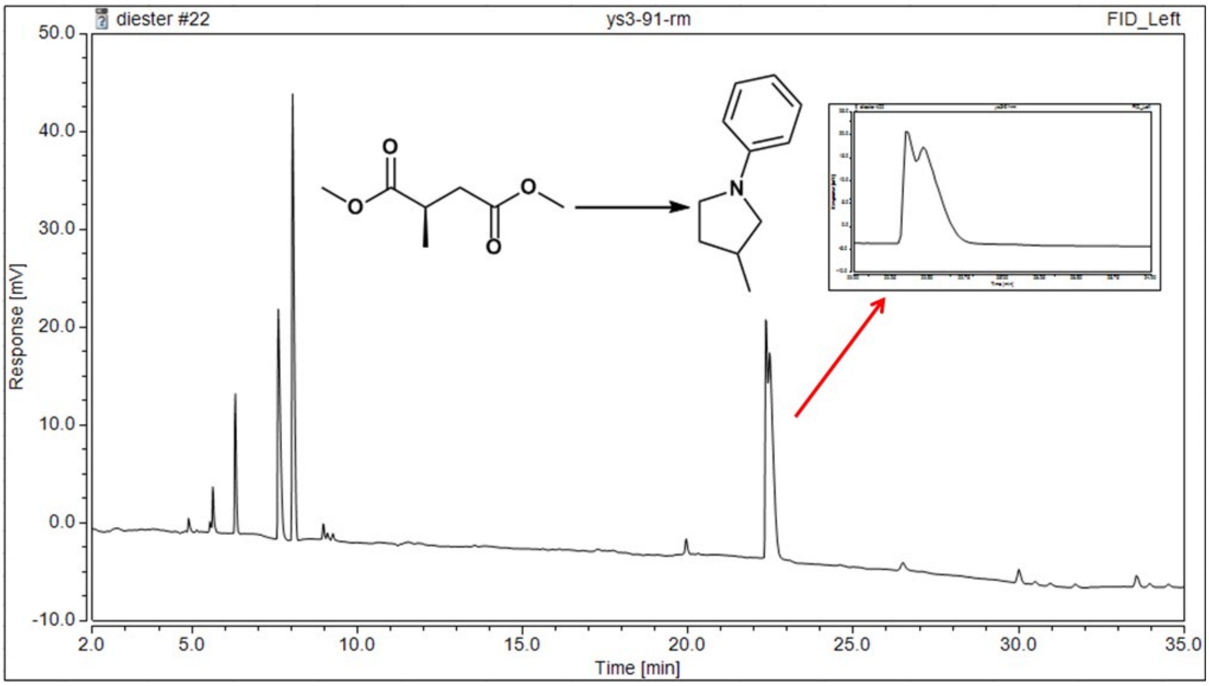

Figure 52. GC spectrum using chiral column for the reaction mixture with dimethyl (*R*)-2-methylsuccinate as substrate.

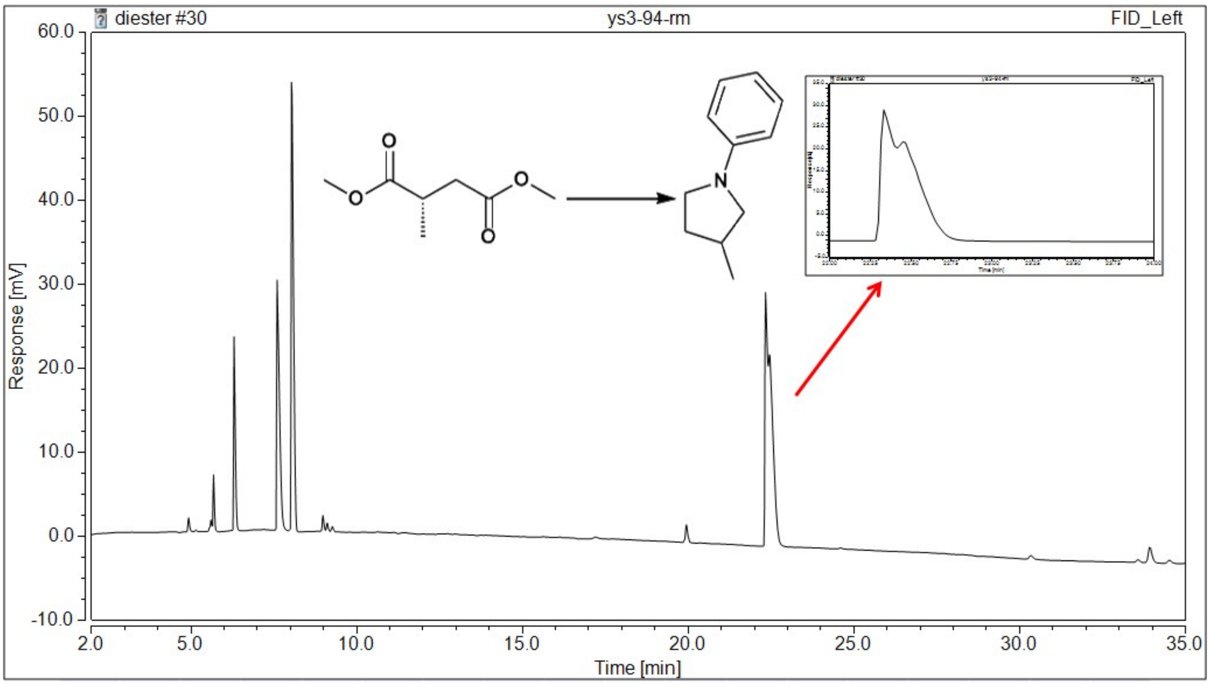

Figure 53. GC spectrum using chiral column for the reaction mixture with dimethyl (*S*)-2-methylsuccinate as substrate.

5. Cyclisation of diesters with various amines

Table S1. Cyclisation of diesters in the presence of various amines.[a]

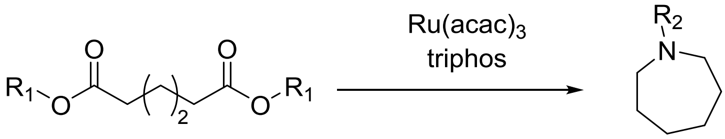

Entry	R_1	Amine	Conversion (%)	Product	Yield (%)
**1**[b]	Me	benzylamine	99	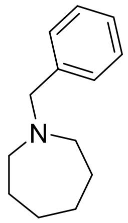	38*

**2**	isobutyl	benzylamine	92	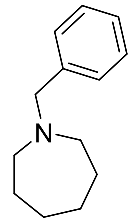	53*
**3**	isobutyl	*n*-propylamine	65	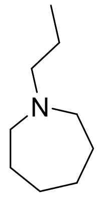	15*
**4**	isobutyl	*n*-butylamine	70	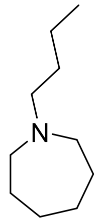	21*
**5**	isobutyl	isopropylamine	90	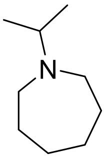	16*
**6**	isobutyl	allyl amine	40	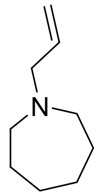	0*
**7**	isobutyl	*tert*-butyl carbamate	50	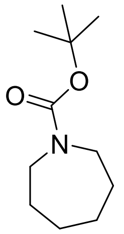	0*
**8**	isobutyl	1,4-dibenzodioxan-6-amine	100	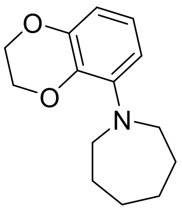	96*
**9**	isobutyl	4-fluoroaniline	100	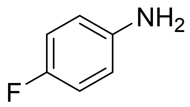	94
**10**	isobutyl	2-fluoroaniline	94	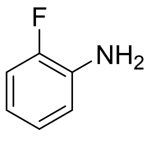	78

**11**	isobutyl	2,6-dimethylaniline	100	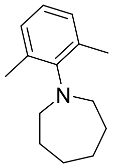	54
**12**	isobutyl	4-nitroaniline	69	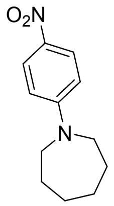	7*
**13**	isobutyl	2,6-dinitroaniline	16	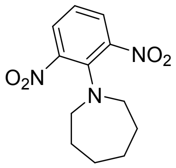	0*

a. *Reagents and conditions*: $Ru(acac)_3$ (2 mol%), triphos(4 mol%), MSA (2 mol%), amine (1.5 equiv.), 1,4-dioxane (15 mL), H_2 (10 bar), 42 h, 220 °C. Conversions and yields are calculated using 1,4-dinitrobenzene as internal standard.

b. *Reagents and conditions*: $Ru(acac)_3$ (1 mol%), triphos (2 mol%), MSA (1 mol%), amine (1.5 equiv.), 1,4-dioxane (15 mL), H_2 (10 bar), 70 h, 220 °C. Conversions and yields are calculated using 1,4-dinitrobenzene as internal standard.

*Reactions are not done in duplicate.

Comment [d]: 3

(1) Britton, J.; Dalziel, S. B.; Raston, C. L. *Green Chem.* **2016**, 2193.

(2) Gu, X.; Zhang, Y.; Xu, Z.-J.; Che, C.-M. *Chem. Commun.,* **2014**, *50*, 7870.

(3) Kreye, O.; Meier, M. A. R. Base catalyzed sustainable synthesis of phenyl esters from carboxylic acids using diphenyl carbonate, 2015.

(4) F. Osamu, Y. Sawa, 2004, EP1491523A1.

(5) Joe, C. L.; Doyle, A. G. *Angew. Chem. Int. Ed.,* **2016**, *55*, 4040.

(6) Yin, J.; Buchwald, S. L. *Org. Lett.,* **2000**, *2*, 1101.

(7) Xue, W.; Xu, H.; Liang, Z.; Qian, Q.; Gong, H. *Org. Lett.,* **2014**, *16*, 4984.

(8) Iranpoor, N.; Panahi, F. *Org. Lett.,* **2015**, *17*, 214.

(9) Al-Amin, M.; Honma, T.; Hoshiya, N.; Shuto, S.; Arisawa, M. *Adv. Synth. Catal.* **2012**, *354*, 1061.

(10) Crawford, S. M.; Lavery, C. B.; Stradiotto, M. *Chem. Eur. J.,* **2013**, *19*, 16760.

(11) H. B. Renfroe, 1984, US4478842A1.
